# Comprehensive analysis of Dof transcription factors in *Dendrobium* species and functional characterization of *DoDof4* in the accumulation of water-soluble polysaccharides

**DOI:** 10.3389/fpls.2025.1617856

**Published:** 2025-08-07

**Authors:** Zhangting Xu, Guihua Zhang, Feixiong Zheng, Xiaoji Deng, Yiming Sun, Jaime A. Teixeira da Silva, Xiaoxia Shen, Zhenming Yu

**Affiliations:** ^1^ School of Pharmaceutical Sciences, Academy of Chinese Medical Sciences, Zhejiang Chinese Medical University, Hangzhou, China; ^2^ Zhejiang Academy of Forestry, Hangzhou, China; ^3^ Independent Researcher, Miki, Kagawa, Japan; ^4^ Songyang Institute, Zhejiang Chinese Medical University, Lishui, China

**Keywords:** Dendrobium orchid, DOF, expression analysis, water-soluble polysaccharides, MeJA

## Abstract

**Introduction:**

*Dendrobium* is a multi-purpose medicinal orchid that grows on the edge of high­altitude cliffs. The content of water-soluble polysaccharides (WSPs), which primarily play a pharmacological role, is the main criterion for evaluating the quality of *Dendrobium* orchid. Therefore, it is necessary to study the regulatory manner involved in the accumulation of WSPs.

**Methods:**

*D. officinale* were treated with methyl jasmonate (MeJA), and WSPs content was measured at different time points to assess the dynamic accumulation pattern. To clarify the role of one finger (Dof) family genes in the MeJA-mediated WSP metabolic pathway, a bioinformatics analysis identified Dof members in *D. huoshanense*, *D. nobile* and *D. officinale*. Based on expression patterns and co-expression analysis, a regulatory factor, *DoDof4* was identified.

**Results:**

In this study, the elicitation of *D. officinale* by methyl jasmonate (MeJA) increased WSP production, which was further amplified by extending the treatment period. Analysis of transcriptomic data revealed that members of the DNA-binding with Dof gene family members accounted for 4% of all differentially expressed genes coding for transcription factors, following MeJA induction. To clarify the role of Dof family genes in the MeJA-mediated WSP metabolic pathway, a bioinformatics analysis identified 29, 29 and 22 Dof members in *D. huoshanense*, *D. nobile* and *D. officinale*, respectively and these were divided into four groups. *DoDof4* was encoded a 31.16 kDa protein composed of 292 amino acids, and was targeted on chromosome 3. Furthermore, *DoDof4* was a typical transcription factor that localized in the nucleus, displayed transcriptional activity and increased the WSPs accumulation. *DoDof4* was co-expressed with 15 genes involved in the WSP metabolic pathway, eight of which displayed a positive Pearson's correlation coefficient. Additionally, correlation analysis revealed the possible downstream targets (KM980199 and KP203853) of *DoDof4*.

**Discussion:**

The results of the present study suggest that *DoDof4* acts as an important regulator in the WSPs metabolic pathway, exhibiting potential values for the improvement of WSPs in *Dendrobium* species.

## 1 Introduction

The orchid, comprising over 29,000 species, is one of the largest and most diverse families of angiosperms (Pérez-Escobar et al., 2024). Among these, *Dendrobium* (almost 1,800 species) was the largest genera in the Orchidaceae. In natural conditions, *Dendrobium* usually grows epiphytically on cliffs at an altitude of 1000–3000 m above sea level, on warm and humid branches of trees or rocks. The stems of *Dendrobium* in both vegetative and reproductive stages are rich in active secondary metabolites, such as alkaloids, flavonoids, and others (Li et al., 2024). The pharmacologically most active components of *Dendrobium* are the water-soluble polysaccharides (WSPs) (Teixeira da Silva and Ng, 2017). WSPs displayed antioxidant, anti-degenerative, anticancer, and immune-regulating effects (Tang et al., 2017). *Dendrobium* WSPs can facilitate an increase in water uptake by plants growing in abiotic stress environments (drought or salt stress), thus improving their abiotic stress tolerance (He et al., 2017; Yu et al., 2017, 2019). Previous research (Yu et al., 2021b; Si et al., 2022) revealed that there are two biosynthetic pathways for the production of WSPs. Initially, sucrose is converted into glucose and fructose, followed by the production of fructose-6P. Phosphomannomutase (PMM) converts fructose-6P to mannose-1P, which is catalyzed by GDP-mannose-pyrophosphorylase (GMP) to generate GDP-mannose. Sucrose is then converted into UDP-glucose, and then into GDP-glucose by Uridine diphosphate glucose pyrophosphorylase (UGP). In addition, UDP-glucose can also generate UDP-galactose in response to UDP glucose 4-epimerase (UGE). Finally, GDP-mannose, GDP-glucose, and UDP-galactose can be transported from the cytoplasm to the Golgi apparatus with the help of GDP-mannose transporter (GMT), where the *D. officinale* WSPs are generated by CELLULOSE SYNTHASE-LIKE A (CSLA) (He et al., 2017). Although there are some inducible methods to increase WSP content in *Dendrobium*, such as moderate stress treatments or the addition of bioinducers (Li et al., 2023), the intrinsic molecular mechanisms underlying the metabolic pathway of WSPs are unknown. Therefore, there is practical value in studying how genes regulate the metabolic pathway of WSPs in *Dendrobium*.

Transcription factors (TFs), also known as *trans*-acting factors, are important regulatory proteins that can affect gene expression and participate in multiple critical biological processes such as signal transduction, stress response, and the synthesis of bioactive compounds (Strader et al., 2021). Based on their protein structure, TFs are generally composed of four structural domains: the N-terminal DNA-binding domain, the C-terminal transcriptional regulatory domain, the nuclear localization signaling domain (NLS), and the oligomerization site (Gupta et al., 2015). The DNA-binding domain can bind to specific bases of the target gene’s promoter, and in doing so, promote or inhibit the expression level of the target gene (Udvardi et al., 2007). Most TFs have only one DNA-binding domain, but some TFs, such as GT2 and APETALA2 of *Arabidopsis thaliana*, contain two DNA-binding domains (Guo et al., 2005). The DNA-binding domain of the same type of TF is a relatively conserved amino acid sequence, so TFs can be categorized into different TF gene families based on their specific and conserved DNA-binding domains. A wide range of TFs have been identified in plants, such as the B-box (BBX), basic leucine zipper (bZIP), WRKYGOK (WRKY), myeloblastosis (MYB), basic helix-loop-helix (bHLH), and others (Tian et al., 2020).

The DNA-binding with one finger (Dof) proteins, including 2,589 members of the ZF superfamily, according to the plant TF database, are widely distributed throughout the plant kingdom, ranging from green unicellular algae to vascular plants, so they are a class of plant-specific TFs (Gupta et al., 2015). Dof TFs were first identified in maize (*Zea mays*) (Yanagisawa and Izui, 1993), and have since been identified in a large number of plant species, such as *A. thaliana* (Xu et al., 2020), *Spinacia oleracea* (Yu et al., 2021a), *Durio zibethinus* (Khaksar et al., 2019), *Maninot esculenta* (Zou et al., 2019), *Vitis vinifera* (Wang et al., 2021) and others. Dof proteins, which contain 100–400 amino acids with a variable sequence of C-terminal transcriptional regulatory domains, as well as 50–52 highly conserved amino acids at the N-terminus that form a specific DNA-binding domain with a characteristic motif CX_2_CX_21_CX_2_C, have the ability to form a single zinc finger structural domain (Kang et al., 2016). The DNA-binding domains of Dof TFs can activate or repress the expression of target genes by specifically binding to bases containing [T/A]AAAG or its complementary sequence CTTT[T/A] (Cominelli et al., 2011). Moreover, when there are two binding sites, the binding capacity of the structural domains of Dof is two-fold higher than that of a single binding site (Zou and Sun, 2023). However, the *Cucurbita moschata* Dof protein recognizes and binds to AAGT of the downstream target gene promoter (Kisu et al., 1998) whereas the *Tamarix chinensis* Dof protein recognizes and binds to TGCG (Wang et al., 2024). The Dof structural domain can bind to specific bases alone to mediate DNA-protein binding. *Sorghum bicolor* Dof21 binds to the P-box (CCTTTTG) element of the promoter of *GRANULE-BOUND STARCH SYNTHASE I* (*SbGBSSI*), a key gene for starch synthesis, and activates its expression (Xiao et al., 2022). The Dof structural domain can also interact with other TFs to mediate protein-protein binding (Wu et al., 2018). *Musa acuminata ETHYLENE RESPONSE FACTOR 9* (MaERF9) activates the expression of fruit ripening-related genes, although MaDof23, a transcriptional repressor that interacts with MaERF9, blocks the binding sites in the promoters of fruit ripening-related genes, thereby reducing the activation effect brought about by MaERF9 in order to delay fruit ripening (Feng et al., 2016). *Prunus avium* (Pav) Dof6 binds directly to the promoters of genes related to cell wall modification to regulate their expression and accelerate fruit ripening, while PavDof2/15 directly regulate fruit softening, by delaying it (Zhai et al., 2022). The PavDof2/15*-*mediated abscisic acid (ABA) signal positively regulates the expression of *9-CIS-EPOXYCAROTENOID DIOXYGENASE 1* (*PavNCED1*), and also interacts with the auxin response element (AuxRE) AUXIN RESPONSE FACTOR 8 (PavARF8), forming the ABA-PavARF8-PavDofs module to indirectly regulate fruit softening (Zhai et al., 2022). These studies indicate that the Dof binding domain is a protein structural domain with dual functions, reflecting the functional diversity of Dof TFs.

In order to understand the roles of Dof in *Dendrobium*, we identified Dof from *D. huoshanense*, *D. nobile* and *D. officinale* genomes, presenting the first systematic comparison of three *Dendrobium* species. Furthermore, we employed *D. officinale* as a model system to elucidate how MeJA regulate the synthesis of WSPs. Our study provides valuable information that would allow WSP content to be amplified in the future by artificially inducing *Dof* gene expression in transgenic plants.

## 2 Materials and methods

### 2.1 Plant materials and MeJA treatment

Protocorm-like bodies (PLBs) were generated using a previously described *in vitro* regeneration protocol (Zeng et al., 2023), and cultivated in Murashige and Skoog (MS) medium (Murashige and Skoog, 1962) containing 0.5 mg·L^-1^ 6-benzyladenine (6-BA; Aladdin, Shanghai, China), 0.2 mg·L^-1^ 1-naphthaleneacetic acid (NAA; Aladdin), 20% banana puree (prepared fresh), 2% (w/v) sucrose (Aladdin) and 0.7% agar to induce proliferation (Zeng et al., 2023). Cultures were placed under controlled environmental conditions (25°C; 12-h photoperiod; 50 µmol m^-2^ s^-1^ cold fluorescent white light). Following the acclimatization of *in vitro*-regenerated plants (Zeng et al., 2023), the leaves of 36-month-old plants with uniform growth were sprayed with 0.1 mM methyl jasmonate (MeJA) (98% purity, Sigma-Aldrich, St. Louis, MO, USA), which was dissolved in trace ethanol (99.5% purity, Merck, Darmstadt, Germany). Leaves were sprayed every 30 d for a total of 90 d to investigate the effect of MeJA on WSP content and transcript levels of *D. officinale* genes. The treated stems were collected at three time points (30, 60, and 90 d), with three replications per time point. Plants sprayed with distilled water supplemented with trace ethanol served as the control (CK). Each treatment group included three replications. Samples were stored immediately at -80°C. Frozen samples of plants that had been treated with 0.1 mM MeJA for 30 d were sent to Biomarker Technologies (Beijing, China) to determine the transcript levels of *D. officinale* genes.

### 2.2 RNA-seq and transcriptome analysis

RNA-seq libraries were carried out as reported (Si et al., 2022), and the cDNA products accord with quality assessment were sequenced through the BMKCloud platform (www.biocloud.net). High-quality clean reads were cherry-picked by removing adapters and low-quality sequences, compared with the chromosome-level *D. officinale* genome, and normalized to fragments per kilobase per million fragment-mapped reads (FPKM). Differentially expressed genes (DEGs) were identified using DESeq2 version 1.38.3 based on a false discovery rate (FDR) < 0.05, and |log_2_FoldChange| > 1. Gene Ontology (GO), Eukaryotic Orthologous Groups (KOG), and Kyoto Encyclopedia of Genes and Genomes (KEGG) enrichment analyses were performed using the BMKCloud server with the *P*-value less than 0.05.

### 2.3 Identification and physicochemical properties analysis of Dof genes

The Hidden Markov Model (HMM) of Dof proteins’ structural domain (PF02701) was downloaded from password-encrypted Interpro (https://www.ebi.ac.uk/interpro/). The whole genome sequences of *D. nobile* (accession no. 94219), *D. huoshanense* (accession no. 154293) and *D. officinale* (accession no. 142615) which were downloaded from IMP (https://www.bic.ac.cn/IMP) were analyzed by HMM Search of TBtools v2.118 (Chen et al., 2023), using an E-value > 0.05 as the filtering criterion to remove redundancy. After removing incomplete sequences in the conserved structural domains, using Conserved Domains Database (https://www.ncbi.nlm.nih.gov/Structure/bwrpsb/bwrpsb.cgi), 22 DoDof proteins, 29 DhDof proteins and 29 DnDof proteins (**Supplementary Tables S1**-**S3**) were finally identified. In addition, the 80 Dof proteins were analyzed at the ExPASy online website (https://www.expasy.org/) to determine the size, molecular weight (MW), isoelectric point (pI), instability index, aliphatic index, and grand average of hydropathicity (GRAVY) for each protein. Finally, the subcellular localization of the Dof proteins were predicted in WoLF PSORT (https://wolfpsort.hgc.jp/).

### 2.4 Phylogenetic analysis and multiple sequence alignment of Dof proteins

To explore the evolutionary relationships between the 80 Dof proteins, they were compared with 36 A*. thaliana* Dof proteins and 30 *Oryza sativa* Dof proteins that were downloaded from PlantTFDB (**Supplementary Table S4**). They were subjected to multiple sequence alignment using ClustalX v2.1 (http://www.clustal.org/). A phylogenetic tree of these 80 proteins was constructed with MEGA v11.0 (https://megasoftware.net/) using the neighbor-joining method (Saitou and Nei, 1987) with a bootstrap value of 1000 and was visualized using Chiplot (https://www.chiplot.online/).

### 2.5 Chromosome localization and collinearity analysis of Dof proteins

Collinearity analysis of the Dof proteins was performed and visualized in TBtools using the One Step MCScanX Super Fast modulation. The genome files and annotation files of *A. thaliana* and *O. sativa*, as representative dicot and monocot model plant, respectively, were downloaded from CNCD-NGDC (https://download.cncb.ac.cn/gwh/Genome/Plants/). Collinearity analyses were performed in TBtools with the Dual Synteny Plot.

### 2.6 Validation of transcriptomic data and investigation of the expression profiles of DoDof genes in different organs by qRT-PCR

Total RNA was isolated from different organs of *D. officinale* (roots, leaves, flowers, and stems), MeJA-treated leaves (0.1 mM, 30 d), and control leaves using the Quick RNA Isolation Kit (0416–50 GK, Huayueyang, Beijing, China). After ensuring the purity and concentration of RNA by 1% agarose gel electrophoresis (Bio-Rad Laboratories, Hercules, CA, USA) and a NanoDrop2000 spectrophotometer (Thermo Fisher Scientific, Waltham, MA, USA), the Evo M-MLV RT Kit II (Accurate Biology, Hunan, China) was used to obtain first-strand cDNA. The quantitative real-time polymerase chain reaction (qRT-PCR) reaction, which contained 5 μL 2× iTaq™ universal SYBR^®^ Green (Bio-Rad Laboratories), 1 μL cDNA, 0.4 μL of each primer (10 μM), and RNase-free water supplemented to 10 μL, was run in an Applied Biosystems 7500 (Applied Biosystems, Foster City, CA, USA). *ELONGATION FACTOR 1 ALPHA* (*EF-1α*) was used as the reference gene (Yu et al., 2021b). Relative gene expression of the 22 *DoDof* genes was calculated by the 2^-ΔΔCt^ method (Livak and Schmittgen, 2001). The quantitative PCR primers, listed in **Supplementary Table S5**, were generated by Integrated DNA Technologies (https://sg.idtdna.com/).

### 2.7 Co-expression analysis between the enzyme-encoding genes and DoDof genes

Multiple enzyme-encoding genes involved in the WSP metabolic pathway were previously identified (Yu et al., 2021b; Si et al., 2022). The correlations between these enzyme-encoding genes and the *DoDof* genes was assessed as Pearson’s correlation coefficients with SPSS v. 27.0 (IBM, Armonk, NY, USA), and a co-expression network was generated in Cytoscape v3.10.0 (https://cytoscape.org/).

### 2.8 Molecular cloning, subcellular localization and transcriptional activation assay of *DoDof4*

The coding sequence of *DoDof4* (without the stop codon TGA) was cloned using the PrimeSTAR Max premix (Takara, Dalian, China). To determine the subcellular localization of DoDof4, *DoDof4* was inserted into the *Spe*I and *Bam*HI sites of a 35S promoter-driven pHB vector (Haq et al., 2021) containing yellow fluorescent protein (YFP) with In-Fusion Cloning Kit (Takara). After verification by sequencing (Zhejiang Sunya Co., Hangzhou, China), recombinant plasmid pHB-*DoDof4*-YFP and the negative control pHB-YFP were transformed into *Agrobacterium tumefaciens* GV3101 (Weidi Biotechnology Co., Shanghai, China) through a previously published freeze-thaw protocol (Yu et al., 2021b), and used to infect the one-month-old *Nicotiana benthamiana* leaves. After 48 h, subcellular localization of YFP in *N. benthamiana* leaves, and the staining leaves with 4’,6-diamidino-2-phenylindole (DAPI; Sigma-Aldrich), were observed on a confocal microscope (Zeiss, Oberkochen, Germany) under a 488 nm excitation filter. To evaluate the transcriptional activation or repression of DoDof4, coding sequence of *DoDof4* without the termination codon TGA was cloned into pGBKT7 (Takara) to construct the pGBKT7-DoDof4 as previously reported (Yu et al., 2021b). Yeast AH109 (Weidi) transformed with pGBKT7-Empty and pGBKT7-DoDof4 were grown on a synthetic dropout (SD) without tryptophan (SD/-Trp) medium and SD without adenine, tryptophan, and histidine (SD/-Ade-Trp-His) medium respectively at 29°C. X-α-Gal (Coolaber, China) was additionally dropped to displayed color reaction. pGBKT7-Lam and pGBKT7-P53 were selected as negative and positive controls, respectively.

### 2.9 Transient transformation of *DoDof4* in *D. officinale* PLBs

The *DoDof4* coding sequence without the termination codon TGA was inserted into pCAMBIA1301 vector (CAMBIA, Canberra, Australia) to overexpress the *DoDof4* gene. *DoDof4* gene into pCAMBIA2300 (CAMBIA) using sg*DoDof4*-F and sg*DoDof4*-R primers, which were designed on the CRISPRdirect website (https://crispr.dbcls.jp/) to construct pCAMBIA2300-*DoDof4*-CRISPR/Cas9 (*DoDof4*CRISPR). The verified plasmids, pCAMBIA1301 (overexpression-empty), pCAMBIA2300-CRISPR/Cas9 (knockout-empty), pCAMBIA1301-*DoDof4* (*DoDof4*OE), and *DoDof4*CRISPR were transferred into 25 mL of A. tumefaciens GV3101 following the method described in *2.8*. A single colony was selected and sub-cultured in yeast mannitol medium containing 0.1 mg·mL^-1^ rifampicin and 0.1 mg·mL^-1^ kanamycin (Macklin, Shanghai, China) for 2 d until OD_600_ was 0.8, then centrifuged at 5000 rpm·min^-1^. MS, which was used as infiltration solution (1 mL) that contained 1 mol·L^-1^ acetosyringone (Sigma-Aldrich), 1.95 g·mL^-1^ 2-morpholinoethanesulfonic acid (MES; Sigma-Aldrich), and 0.1% Tween-20 (Sigma-Aldrich), was injected to a depth of 0.5 cm into the center of each *D. officinale* PLB. PLBs were subsequently soaked in the infiltration solution for 4 d (25°C, 24h darkness) to generate pCAMBIA1301, *DoDof4*OE, pCAMBIA2300-CRISPR/Cas9 and *DoDof4*CRISPR lines, respectively. pCAMBIA1301 and *DoDof4*OE transgenic lines were selected on PLB proliferation medium containing 30 mg·L^-1^ hygromycin B (Yeasen). pCAMBIA2300-CRISPR/Cas9 and *DoDof4*CRISPR transgenic lines were selected on PLB proliferation medium containing 25 mg·L^-1^ kanamycin. After 30 d of transient transfection, PLBs were collected and stored immediately at -80°C. Using the technique outlined in 2.7., total RNA was extracted from transgenic PLBs. *DoDof4* expression was examined by qRT-PCR (see *2.6.*). The primers used for qRT-PCR are listed in **Supplementary Table S6**.

### 2.10 Extraction and determination of WSPs in *D. officinale*

WSPs in *D. officinale* samples (100 mg of leaves or PLBs) were ultrasonically extracted using an established method (Yu et al., 2018). WSP content was determined on an UV-3600 multi-directional spectrophotometer (Shimadzu, Kyoto, Japan) at 488 nm using the phenol-concentrated sulfuric acid method (Ogura et al., 2023) and was expressed as mg·g^-1^. To investigate the composition of WSPs, rapid hydrolysis and pre-column derivatization were executed using 0.5 mol·L^-1^ 1-phenyl-3-methyl-5-pyrazolopyrimidone (PMP; Sigma-Aldrich), as described by Yu et al. (2019). Thereafter, 5 μL of filtered supernatant was injected into an Agilent 1260 (Santa Clara, CA, USA) equipped with an Ultimate XB-C18 column (Welch Materials, Shanghai, China) to detect monosaccharide content. Glucose (Sigma-Aldrich) and mannose (Sigma-Aldrich) were used as internal standards, and their content was expressed as mg·g^-1^.

### 2.11 Statistical analysis

All experiments in this paper were performed as three or more independent replicates. The mean ± standard deviation (SD) of the data were calculated and graphs were created in GraphPad Prism v. 8.0.2 (GraphPad, Boston, MA, USA). Statistical analyses were performed in SPSS Statistics v. 27.0. In all graphs, *** represents a very highly significant difference (*P* < 0.001), ** represents a highly significant difference (*P* < 0.01), and * represents a significant difference (*P* < 0.05). RNA-Seq analysis (including transcriptome sequencing, library preparation, transcriptome sequencing, and transcriptome analysis) was conducted at BMKCloud (https://www.biocloud.net/).

## 3 Results

### 3.1 MeJA stimulated the accumulation of WSPs in *D. officinale*

**Figure 1:**
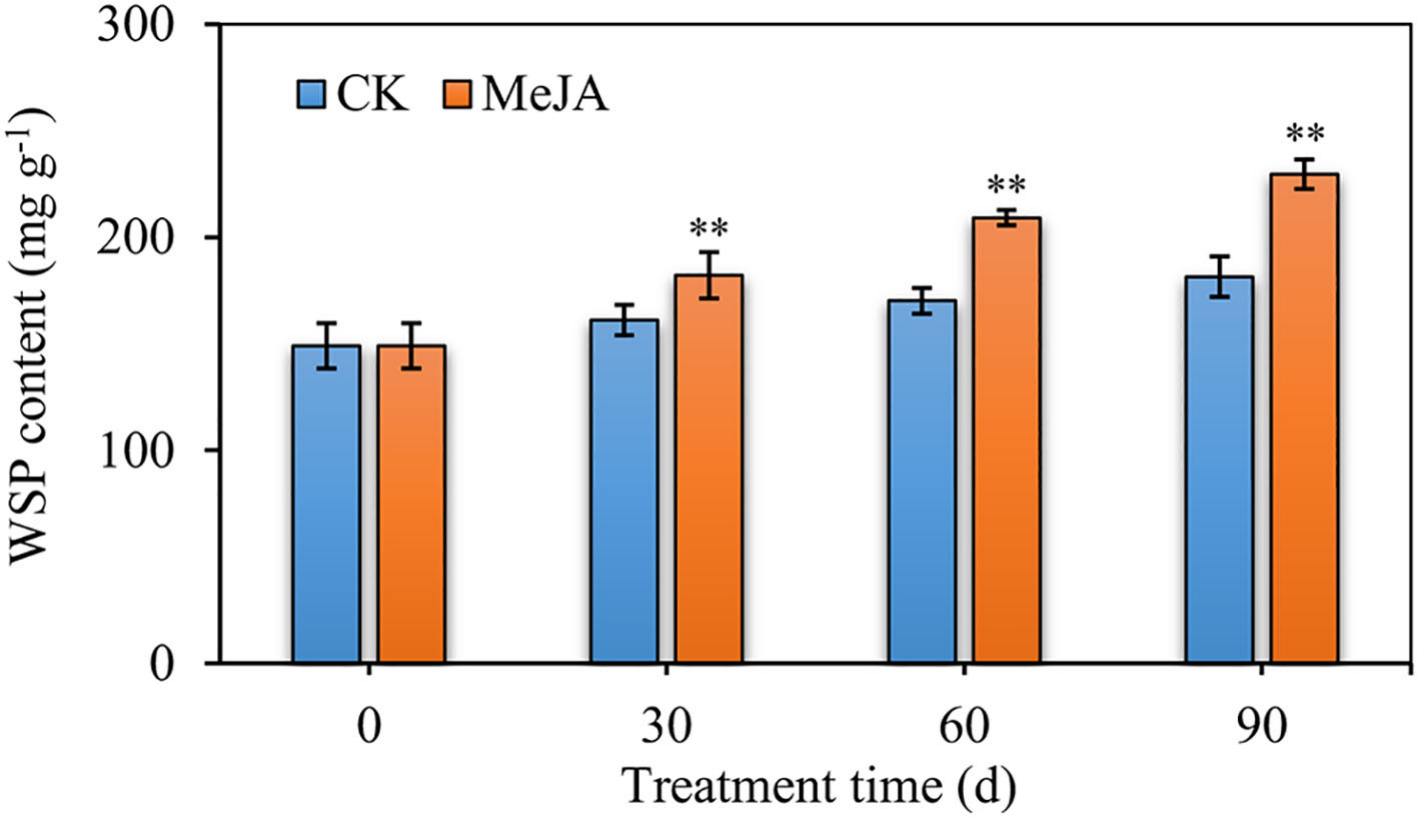
The water-soluble polysaccharide (WSPs) content of 36-month-old *D. officinale* plants after MeJA treatment for 30, 60, and 90 d. CK represents the control group and MeJA represents the experimental group induced by 0.1 mM MeJA. Mean values and standard deviations (SDs) indicated by error bars. Significant differences: **(*P* < 0.01).

Various hormones, as well as biotic and abiotic stresses affect plant health and growth, and ultimately influence the regulation of a number of related genes. MeJA is a signaling molecule that stimulates stress responses and induces the synthesis of a wide range of metabolites. We took *D. officinale* as a model system to explore the effect of MeJA on *Dendrobium* species. WSP content increased in MeJA-treated plants, when compared to the control, even more so when treatment period was extended from 30 to 90 d. These findings confirmed that MeJA promotes the accumulation of WSPs in *D. officinale* (**Figure 1**).

### 3.2 Transcriptomic analysis of *D. officinale* after MeJA treat

**Figure 2:**
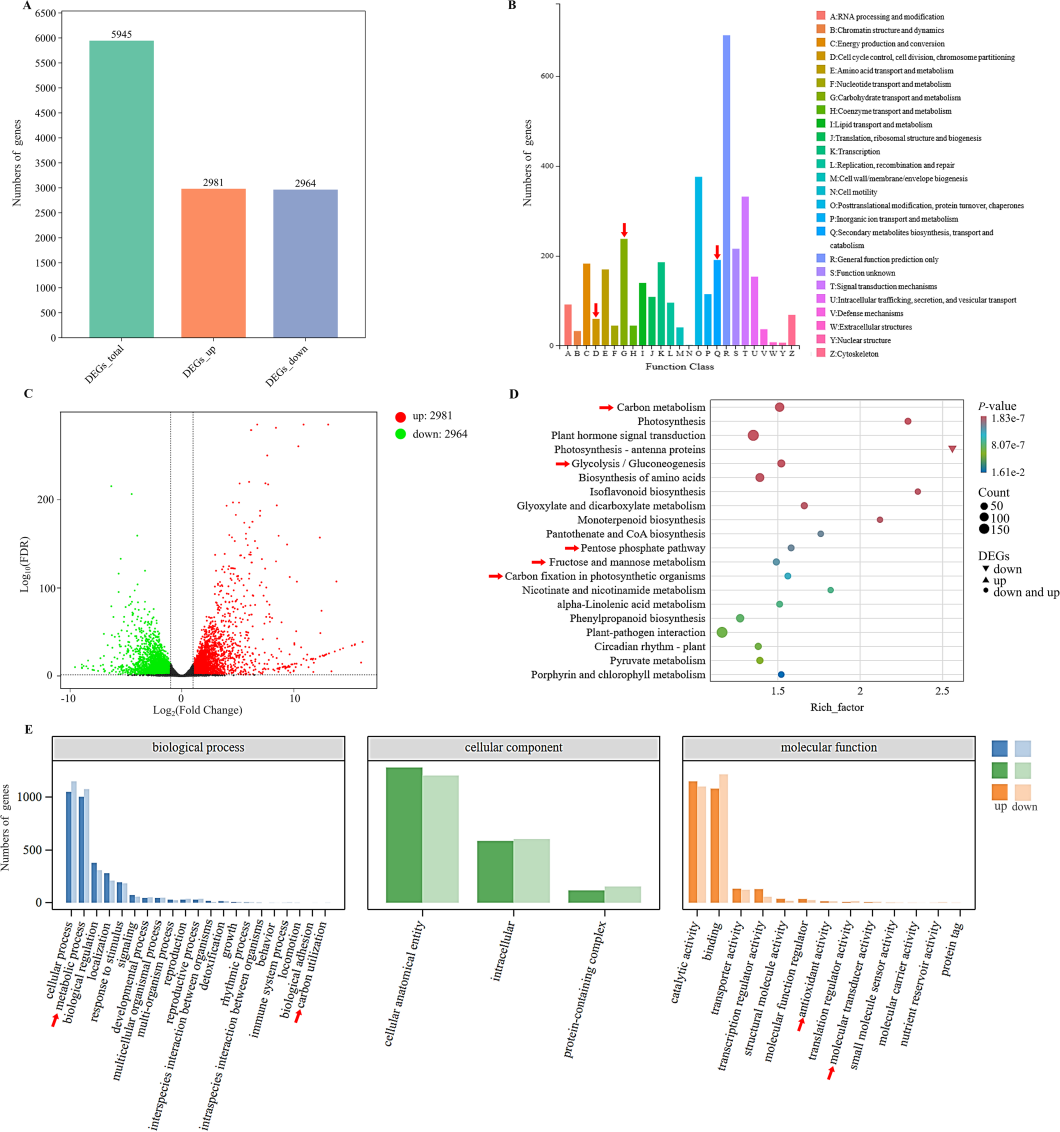
Differential transcriptomic analysis between the control and MeJA-treated samples. **(A)** Distribution of total differentially expressed genes (DEGs), up-regulated DEGs and down-regulated DEGs with relative expression levels. **(B)** KOG function annotation analysis. A to Z represent the 24 distinct KOG functional categories. **(C)** Volcano plot of DEGs. Red, green, and black represent up-regulated DEGs, down-regulated DEGs, and DEGs that did not display significant changes, respectively. **(D)** KEGG functional annotation of DEGs. The colors of circles or triangles represent different *P*-values and the size corresponds to the number of enriched genes. **(E)** GO functional annotation of DEGs. Red arrows highlight functional modules involved in the biosynthesis of water-soluble polysaccharides.

**Figure 3:**
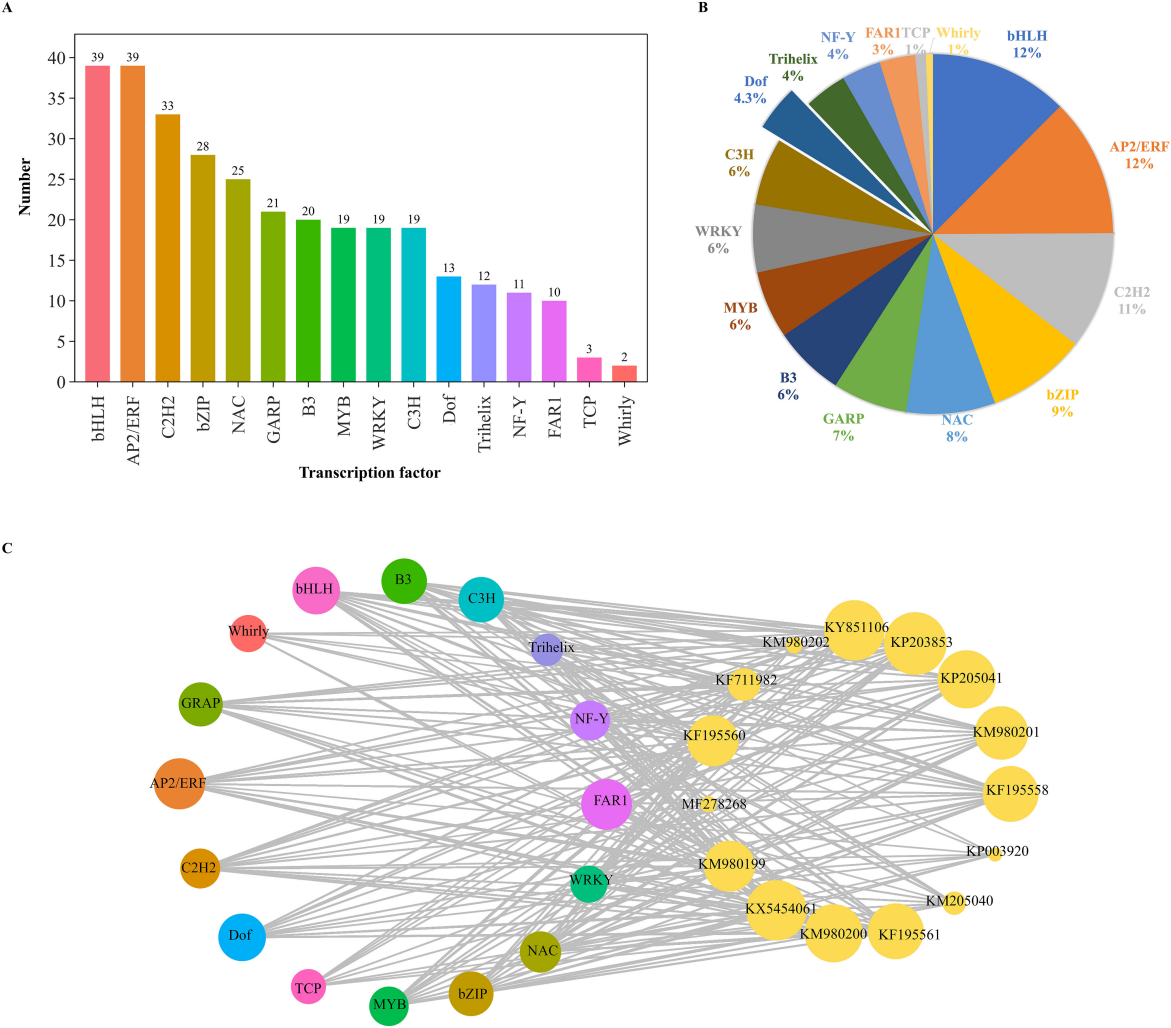
Analysis of differentially expressed transcription factors. **(A)** Number of DEGs encoding transcription factors. **(B)** Relative proportion of DEGs encoding transcription factors. **(C)** Correlation of the expression between polysaccharide biosynthetic genes and *DoDof* genes. *DoGMT* (KY851106, MF278268): GDP-mannose transporter, *DoGMP* (KF195560, KP203853): GDP-mannose-pyrophosphorylase, *DoCSLA* (KM980199, KM980200, KP003920, KM980201, KM980202, KF195561, KP205040, KP205041): CELLULOSE SYNTHASE-LIKE A, *DoPMM* (KF195558): Phosphomannomutase, *DoUGP* (KF711982): Uridine diphosphate glucose pyrophosphorylase, DoUGE (KX54540): UDP glucose 4-epimerase.

To examine the transcriptomic changes in plants following MeJA treatment, PE150 sequencing was performed on six *D. officinale* samples (MeJA-treated and distilled water-treated with trace ethanol, each in three replicates) using the Illumina NovaSeq6000 sequencing platform. After sequencing quality control, a total of 37.35 Gb of clean data was obtained, including 5.79 Gb of clean data in each sample, and the Q30 base percentage was at least 92.28%. The number of clean reads in each sample ranged from 19,347,994 to 22,825,395. Compared to the high-quality *D. officinale* genome, the alignment efficiency of clean reads was between 87.66 and 89.77%. Based on the transcriptomic data, a total of 5945 differentially expressed genes (DEGs) were screened, including 2981 up-regulated DEGs and 2954 down-regulated DEGs (**Figure 2A**), accounting for about half of all DEGs. The functions of these DEGs were analyzed by clusters of eukaryotic orthologous groups (KOG) annotation, revealing a total of 24 categories that were annotated based on the orthologous and evolutionary relationships between eukaryotes (**Figure 2B**). Based on KOG, only ‘general function prediction’ was most annotated functional classification, followed by ‘post translation modification, protein turnover, chaperones’, and ‘signal transduction mechanisms’, while ‘nuclear structure and cytoskeleton’ had fewest annotations. Intuitively, ‘energy production and conversion’, ‘carbohydrate transport and metabolism’, and ‘secondary metabolites biosynthesis, transport and catabolism’ were closely related to WSP biosynthesis. Volcano plots were used to show differential changes in the expression of DEGs, indicating large and significant differences in up-regulated DEGs (**Figure 2C**). KEGG enrichment analysis showed a total of 5945 DEGs that were annotated in KEGG pathways, and 20 pathways had highest *P-*values (**Figure 2D**). Among them, up-regulated DEGs were mainly clustered into the ‘plants hormone signal transduction pathway’, followed by ‘carbon metabolism’. For carbon metabolism, ‘glycolysis/gluconeogenesis’, ‘pentose phosphate pathway’, ‘fructose and mannose metabolism’, and ‘carbon fixation in photosynthetic organisms’ were associated with WSP biosynthesis. For the down-regulated DEGs, the only pathway was ‘photosynthesis-antenna protein’. In the biological pathways from GO enrichment analysis, the largest group was biological processes (BP), followed by molecular functions (MF) (**Figure 2E**). The number of up-regulated and down-regulated DEGs differed for each function. In general, the DEGs were concentrated in ‘cellular and metabolic processes’, ‘cellular anatomical entity’, and ‘catalytic activity and binding’. In addition, 16 TFs were predicted among the DEGs (**Figure 3A**), and seven TFs (bHLH, AP2/ERF, C2H2, bZIP, NAM/ATAF1/2/CUC2 (NAC), Golden2, ARR-B, Psr1 (GARP), B3) from over 20 DEGs accounted for 7/20 of all TFs. The number of predicted DEG members in MYB, WRKY, C3H, Dof, Trihelix, Nuclear Factor Y (NF-Y), and Fatty Acyl-CoA Reductase 1 (FAR1) TF families ranged from 10 to 20, and the number of members in TCP and Whirly TF families was less than 10 (**Figure 3B**). The WRKY, bZIP, AP2/ERF, MYB, and bHLH TF families were previously identified in *D. officinale* (He et al., 2017; Zeng et al., 2021; Li et al., 2022), whereas the families of TFs that the *D. officinale* Dofs belong to remain unclear. Furthermore, expression of the enzyme-encoding genes involved in WSP biosynthesis was positively correlated with *Dof* genes (Pearson’s R^2^ > 0.8) (**Figure 3C**). Therefore, the *Dof* genes were studied in more detail to appreciate how they regulate the accumulation of WSPs.

### 3.3 Identification and physicochemical properties analysis of *Dof* genes

After screening the *D. nobile*, *D. huoshanense* and *D. officinale* genome following the characteristic Dof structural domain (PF02701), and removing redundant and incomplete sequences, 80 *Dof* genes were identified. Among these Dof proteins, *D. nobile* and *D. huoshanense* have an identical number (*n* = 29) of Dof genes, and the number of DoDof genes were lowest (*n*= 22). Based on a distribution of individual *Dof* genes on the corresponding chromosomes, they were sequentially named *DhDof1*-*DhDof29*, *DnDof1*-*DnDof29*, and *DoDof1*-*DoDof22*.

To better understand the roles and mechanisms of these Dof proteins, we systematically predicted and analyzed their physicochemical properties (**Supplementary Table S7**). Our results revealed that three *Dendrobium* species exhibited similar levels in terms of average size (**Supplementary Figure S1A**), MW (**Supplementary Figure S1B**), pI (**Supplementary Figure S1C**), instability index (**Supplementary Figure S1D**), aliphatic index (**Supplementary Figure S1E**), and GRAVY (**Supplementary Figure S1F**) for their Dof proteins. The size in the Dof proteins ranged from 109 (DnDof7) to 626 (DhDof1), and their molecular weights ranged between 12.05 (DnDof7) and 71.12 kDa (DhDof1), and their pIs ranged from 5.17 (DnDof14) to 9.79 (DoDof3). The instability index of all Dof proteins exceeded 40, which indicates that they are highly stable. Combined with aliphatic index and GRAVY, all Dof proteins were classified as hydrophilic proteins.

### 3.4 Phylogenetic analysis and multiple sequence alignment Dof proteins

**Figure 4:**
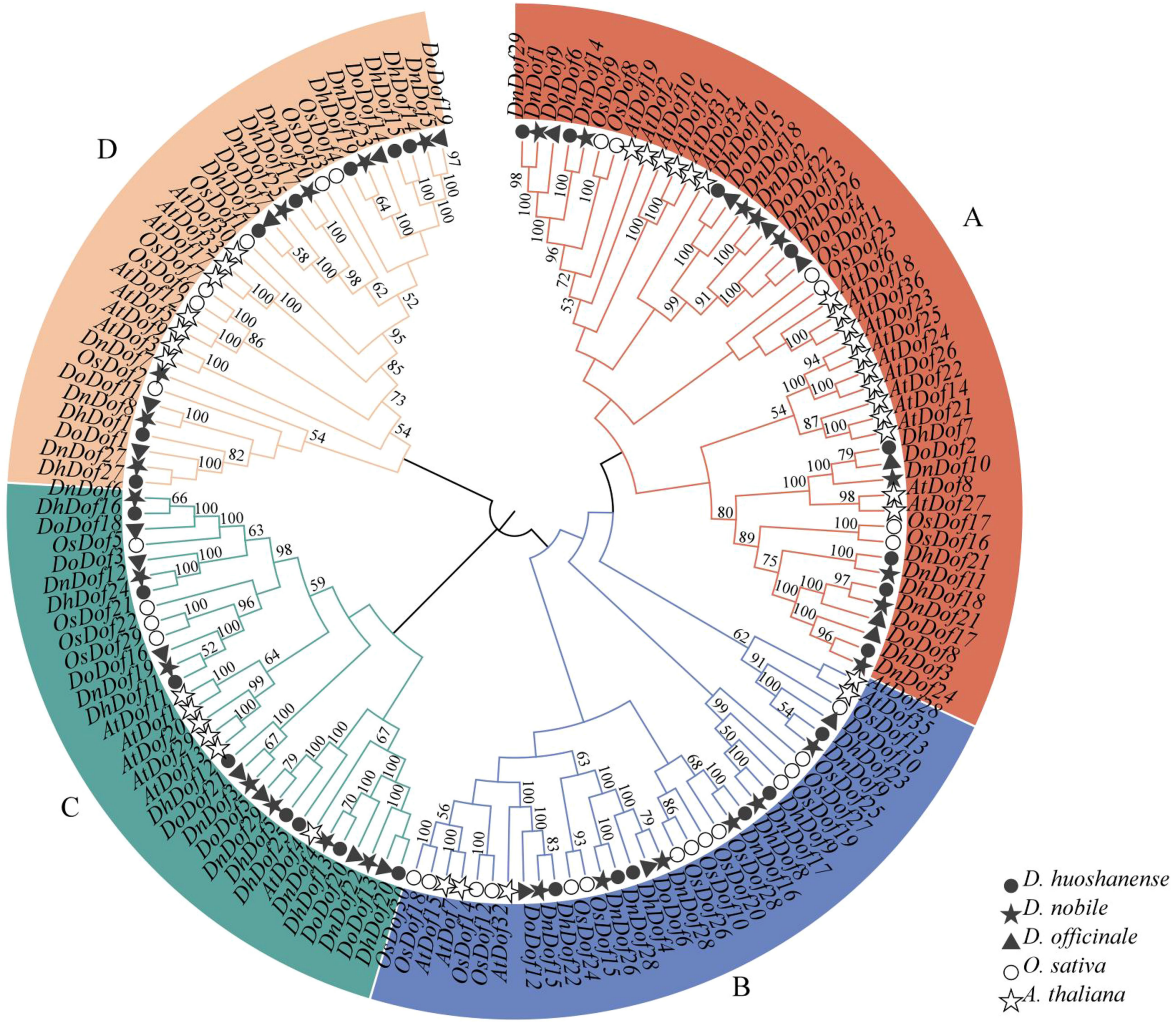
Phylogenetic relationship between the *DhDof*, *DnDof*, *DoDof*, *OsDof*, and *AtDof* genes. The phylogenetic tree was generated with MEGA software using the neighbor-joining method with 1000 bootstrap values. **(A–D)** are defined based on the *Arabidopsis thaliana* Dof family.

To explore the evolutionary relationships within the Dof gene family of *Dendrobium*, a phylogenetic tree of *AtDof*, *OsDof*, *DhDof*, *DnDof* and *DoDof* genes was shown in **Figure 4**. *Dof* genes were divided into four groups according to their phylogenetic relationships (**Figure 4**). Group A contained the largest number of Dof genes (*n* = 48), groups B/C/D contained 34, 32, and 32 Dof genes. Further analysis revealed that the Dof genes from *Dendrobium* species clustered closely together, rather than randomly distributed with those from *A. thaliana* and *O. sativa*. Multiple sequence alignment showed that all *Dof* genes consist of 50 highly conserved amino acids that form a CX_2_CX_21_CX_2_C sequence at the N-terminus, namely the Dof structural domain (**Supplementary Figure S2**). This suggests that Dof genes are relatively conserved during the evolution of *Dendrobium* species.

### 3.5 Chromosome localization and collinearity analysis of Dof genes

**Supplementary Figure S3** showed the location of the Dof genes in *D. huoshanense* (**Supplementary Figure S3A**), *D. nobile* (**Supplementary Figure S3B**) and *D. officinale* (**Supplementary Figure S3C**) chromosomes. The 29 *DhDof* genes were unevenly mapped on the 15 chromosomes while no *DhDof* genes localized on Chr3/10/12/15. Chr8 possessed the highest number (*n* = 5) of *DoDof* genes (*DoDof12-DoDof 16*). The 29 *DhDof* genes exhibited random distribution across 15 chromosomes, mirroring the distribution pattern observed in *D. huoshanense*. Among all chromosomes, Chr3 exhibited the highest density (*n* = 5) of *DhDof* genes (*DhDof2*-*DhDof6*) while Chr2/7/13/16 lacked *DhDof* genes. The 22 *DoDof* genes were unevenly distributed across the 13 chromosomes. Chr19 had the highest number (*n* = 5) of *DoDof* genes (*DoDof18-DoDof 22*) while five chromosomes (Chr7/8/10/15/16) had no *DoDof* genes. An interspecies (i.e., between three *Dendrobium* species) analysis revealed that *D. officinale* and *D. nobile* displayed the highest level of collinearity (*n* = 32), and *D. officinale* and *D. huoshanense* had 21 pairs (**Supplementary Figure S4A**). This indicates that *D. officinale* and *D. nobile* have a closer relationship than *D. officinale* and *D. huoshanense*. In addition, there were 25 collinear genes when compared with *A. thaliana*, and even fewer (*n* = 8) between *D. officinale* and *O. sativa* (**Supplementary Figure S4B**).

### 3.6 Tissue-specific expression analysis of *DoDof* genes

**Figure 5:**
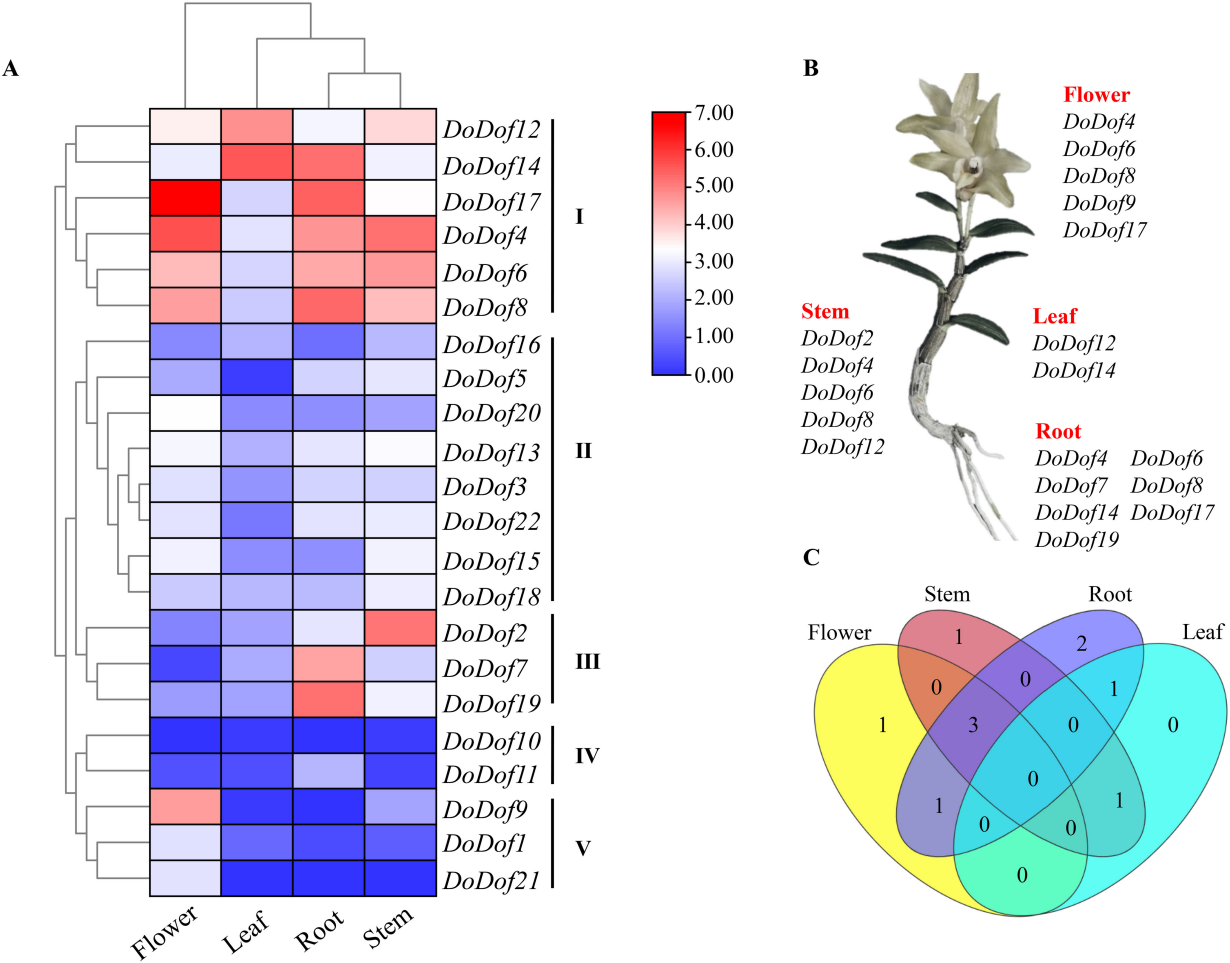
Expression pattern analysis of *DoDof* genes. **(A)** Tissue-specific expression in flower, leaf, root, and stem of *D*. *officinale*. Red and blue in the heatmap represent high and low relative expression, respectively. **(B)** Identification of highly expressed *DoDof* genes in each tissue. **(C)** Venn diagram of *DoDof* genes in different tissues.

The expression of genes in plants is closely related to their functions, so the expression of four tissues in *D. officinale* (flower, leaf, stem, and root) were analyzed (**Supplementary Table S8**). The expression pattern revealed that 22 *DoDof* genes exhibited a distinct organ-specific expression. These were divided further into 5 groups I-V (**Figure 5A**). In group I, 6 *DoDof* genes exhibited high expression in many tissues. For example, *DoDof12* exhibited high expression in leaf and stem, *DoDof14* had high expression in root and leaf, *DoDof17* showed high expression in flower and root, and *DoDof4*/*6*/*8* had high expression in flower, root, and stem. In group II, eight genes (*DoDof3*, *DoDof5*, *DoDof13*, *DoDof15*, *DoDof16*, *DoDof18*, *DoDof20*, and *DoDof22*), and in group III, *DoDof2*, *DoDof7*, and *DoDof19* displayed high expression in root and stem, and relatively uniform expression in flower, root, and stem. *DoDof10* and *DoDof11*, which formed part of group IV, displayed low expression in all tissues. In group V, the remaining *DoDof* genes were expressed mainly in flower and had similarly low expression in other tissues. Overall, most genes were highly expressed in flower, but many genes were also expressed in root and stem. Highly expressed *DoDof* genes were also identified in each tissue (**Figure 5B**). Compared with the expression patterns of the 22 *DoDof* genes, *DoDof17* displayed the highest expression in flower and root, and *DoDof14* displayed the highest expression in leaf. Expression in stem was dominated by *DoDof2* and *DoDof4*, then by *DoDof6*, and finally *DoDof8* and *DoDof12.* A Venn diagram (**Figure 5C**) revealed that no genes were highly expressed in all tissues, while three genes (*DoDof4/6/8*) were highly expressed in flower, stem, and root.

### 3.7 Expression patterns of DoDof genes following treatment with MeJA

**Figure 6:**
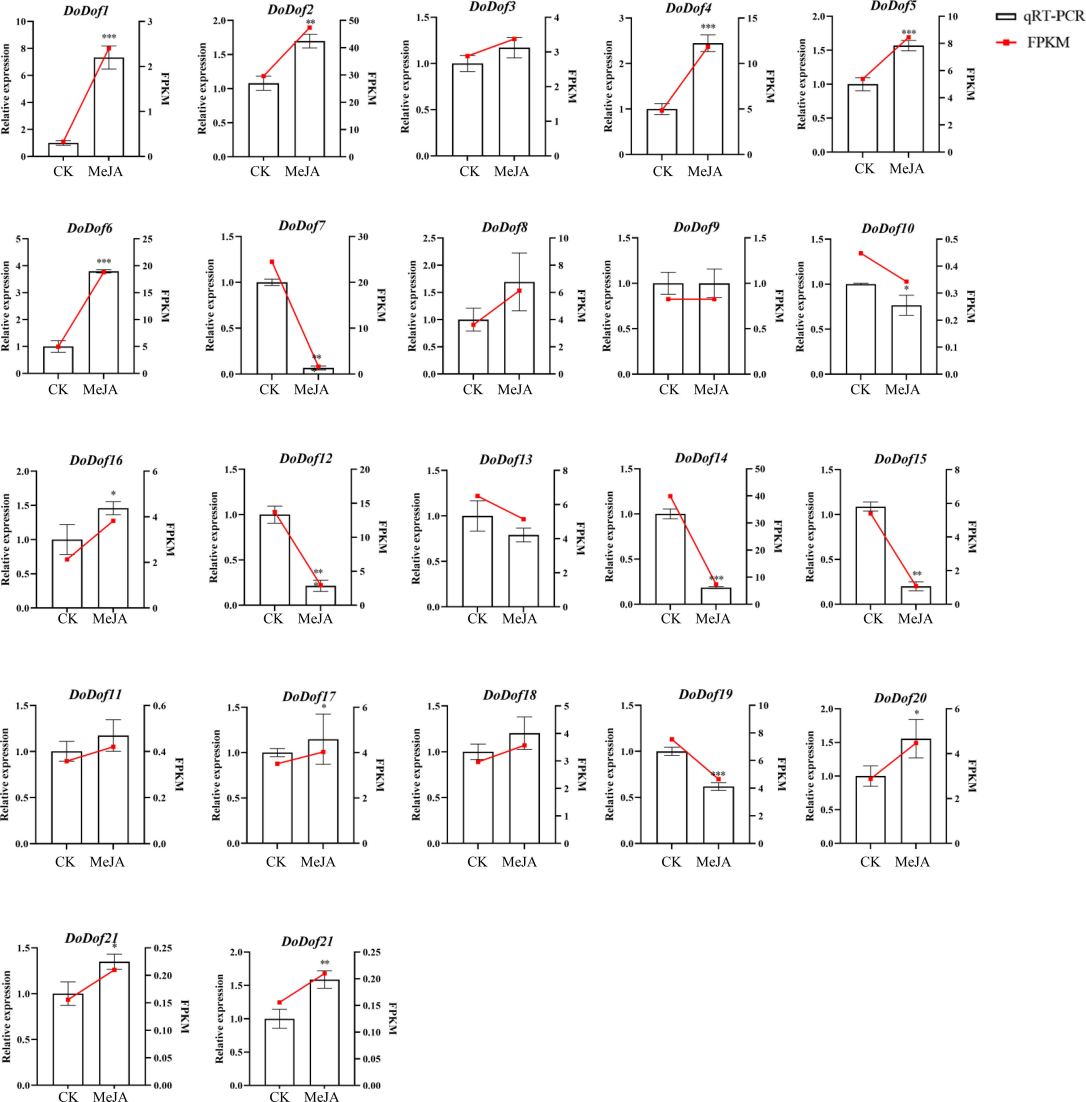
Expression levels of *DoDof* genes induced by MeJA, as determined by qRT-PCR. The X-axis shows the different treatments of PLBs: CK represents the control group and MeJA represents the experimental group induced by 0.1 mM MeJA. The Y-axis represents the relative expression level. Mean values and standard deviations (SDs) indicated by error bars. Significant differences: *(*P* < 0.05), **(*P* < 0.01), and ***(*P* < 0.001).

In order to assess the relationships between *DoDof* genes and MeJA, the relative expression levels of *DoDof* genes were examined by qRT-PCR after 30 d of MeJA treatment to validate the corresponding transcriptomic data. The change in patterns of *DoDof* genes was consistent with the trend of the transcriptome data, which indicates that the transcriptomic data was reliable (**Figure 6**). After MeJA induction, the expression of 14 *DoDof* genes (*DoDof1*/*2*/*3*/*4*/*5*/*6*/*8*/*11*/*16*/*17*/*18*/*20*/*21*/*22*) was up-regulated. The expression of *DoDof9* did not change before and after induction, and the remaining seven genes showed different degrees of down-regulated expression after induction. *DoDof4*/*6* were highly up-regulated, 2.44- and 3.79-fold, respectively, after MeJA treatment. Very few genes of same subfamilies showed same expression trend. For example, relative expression of *DoDof1*/*11* was lower before and after induction, and relative expression of *DoDof7*/*14*/*19* decreased after induction. These findings suggest that MeJA has a significant effect on expression of *DoDof* genes and may thus play a role in the regulation of plant WSP biosynthesis.

### 3.8 Correlation analysis between DoDof genes and enzyme-encoding genes involved in the biosynthesis of water-soluble polysaccharides

**Figure 7:**
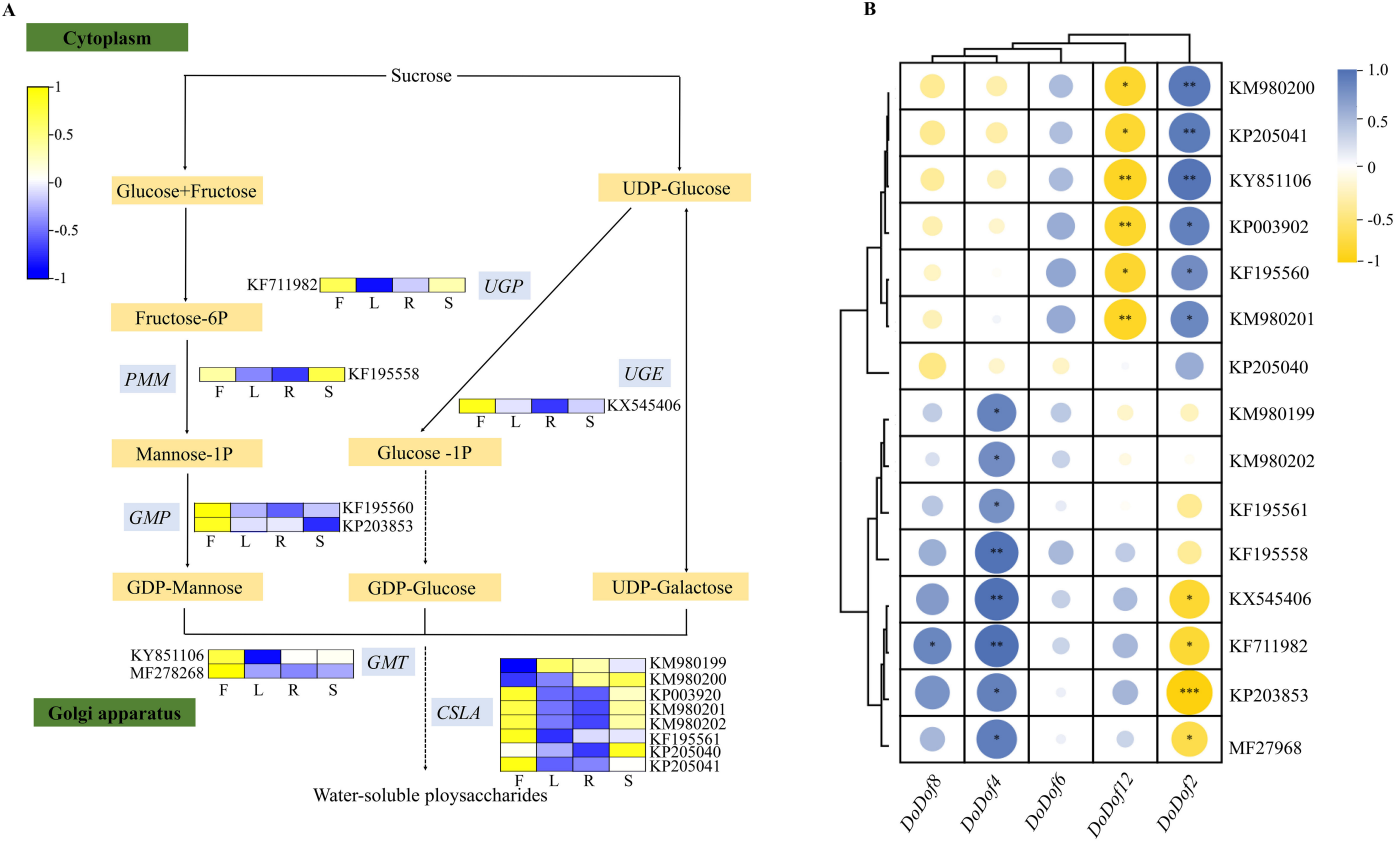
Expression of enzyme-encoding genes involved in the metabolic pathway of water-soluble polysaccharides (WSPs), and a correlation analysis with *DoDof* genes. **(A)** Tissue-specific expression of enzyme-encoding genes involved in the metabolic pathway of WSPs. F, L, R, and S in the heatmap denote flower, leaf, root, and stem tissues, respectively. *DoPMM* (KF195558): Phosphomannomutase, *DoGMP* (KF195560, KP203853): GDPmannose-pyrophosphorylase, *DoGMT* (KY851106, MF278268): GDP-mannose transporter, *DoUGP* (KF711982): Uridine diphosphate glucose pyrophosphorylase, *DoUGE* (KX54540): UDP glucose 4-epimerase, *DoCSLA* (KM980199, KM980200, KP003920, KM980201, KM980202, KF195561, KP205040, KP205041): CELLULOSE SYNTHASE-LIKE. **(B)** Correlation analysis between enzyme-encoding genes and DoDof genes. Significant differences: *(*P* < 0.05), **(*P* < 0.01), and ***(*P* < 0.001).

*DoPMM* (KF195558), *DoGMP* (KF195560, KP203853), *DoGMT* (KY851106, MF278268), *DoUGP* (KF711982), *DoUGE* (KX54540), and *DoCSLA* (KM980199, KM980200, KP003920, KM980201, KM980202, KF195561, KP205040, KP205041), which were highly expressed in flowers and stems (**Figure 7A**), were identified. According to the 2020 edition of the Chinese Pharmacopoeia (Chinese Pharmacopoeia Commission, 2020), the stems of *D. officinale* are usually used as medicine, while the flowers, roots, and leaves are non-medicinal parts. Analysis of the expression of *DoDof* genes in different tissues revealed that *DoDof2*/*4*/*6*/*8*/*12* had highest expression in stem, so they were selected for correlation analysis with these 16 enzyme-encoding genes by SPSS Statistics version 27.0 (**Figure 7B**). *DoDof4* had a notable co-expression relationship with 8 enzyme-encoding genes (KM980199, KM980202, KF195561, KF195558, KX545406, KF711982, KP203853, and MF27968). *DoDof2* was correlated with 6 enzyme-encoding genes (KP203853, KX545406, KF711982, MF27968, KF195561, and KF195558), *DoDof8* was highly and positively correlated with KF711982, no enzyme-encoding gene had a high positive correlation with *DoDof6* or *DoDof12*.

### 3.9 Subcellular localization and transcriptional activation assay of *DoDof4*

**Figure 8:**
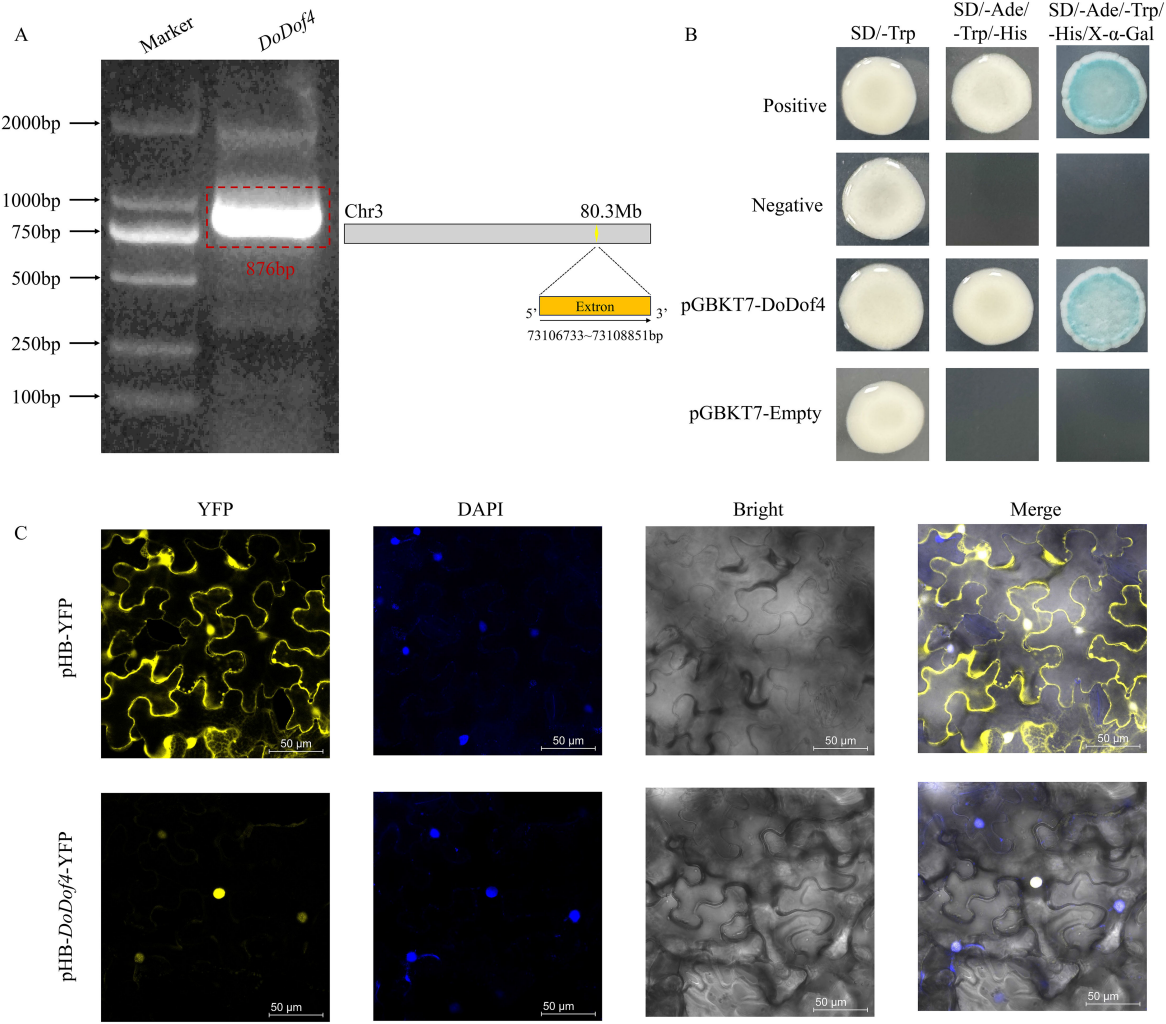
**(A)** Cloning of the gene. **(B)** Transcriptional activation of DoDof4 in yeast cells. SD/-Trp: Synthetic dropout medium-Tryptophan. SD/-Ade/-Trp/-His: Synthetic dropout medium-Adenine/-Tryptophan/-Histidine. SD/-Ade/-Trp/-His/X-α-Gal: Synthetic dropout medium-Adenine/-Tryptophan/-Histidine with X-α-Gal. **(C)** Subcellular localization. pHB-YFP, as the positive control, or pHB-*DoDof4*-YFP, were individually transiently infected into *Nicotiana benthamiana* leaves. YFP is a nucleus and membrane co-localized signal. Scale bars = 50 μm.

*DoDof4* showed 99.65% similarity with the original sequence. *DoDof4* open reading frame (ORF) consisted of 876 bp encoding 292 amino acids (**Figure 8A**) and the predicted molecular weight was 27.84 kDa. The protein sequence contains one Dof domain at the N-terminus indicating that cloned *DoDof4* belongs to the Dof family. Subcellular localization analyses revealed that pHB-*DoDof4*-YFP was localized in the nucleus (**Figure 8C**). Furthermore, pGBKT7-DoDof4 plasmid and positive control grew well on SD/-Ade-Trp-His medium, which degraded the X-α-Gal and changed the color of yeast colony from white to blue. pGBKT7-Empty and negative control did not grow on the SD/-Ade-Trp-His medium (**Figure 8B**), suggesting that DoDof4 was a transcriptional activator.

### 3.10 Transient transformation validated of *DoDof4*

**Figure 9:**
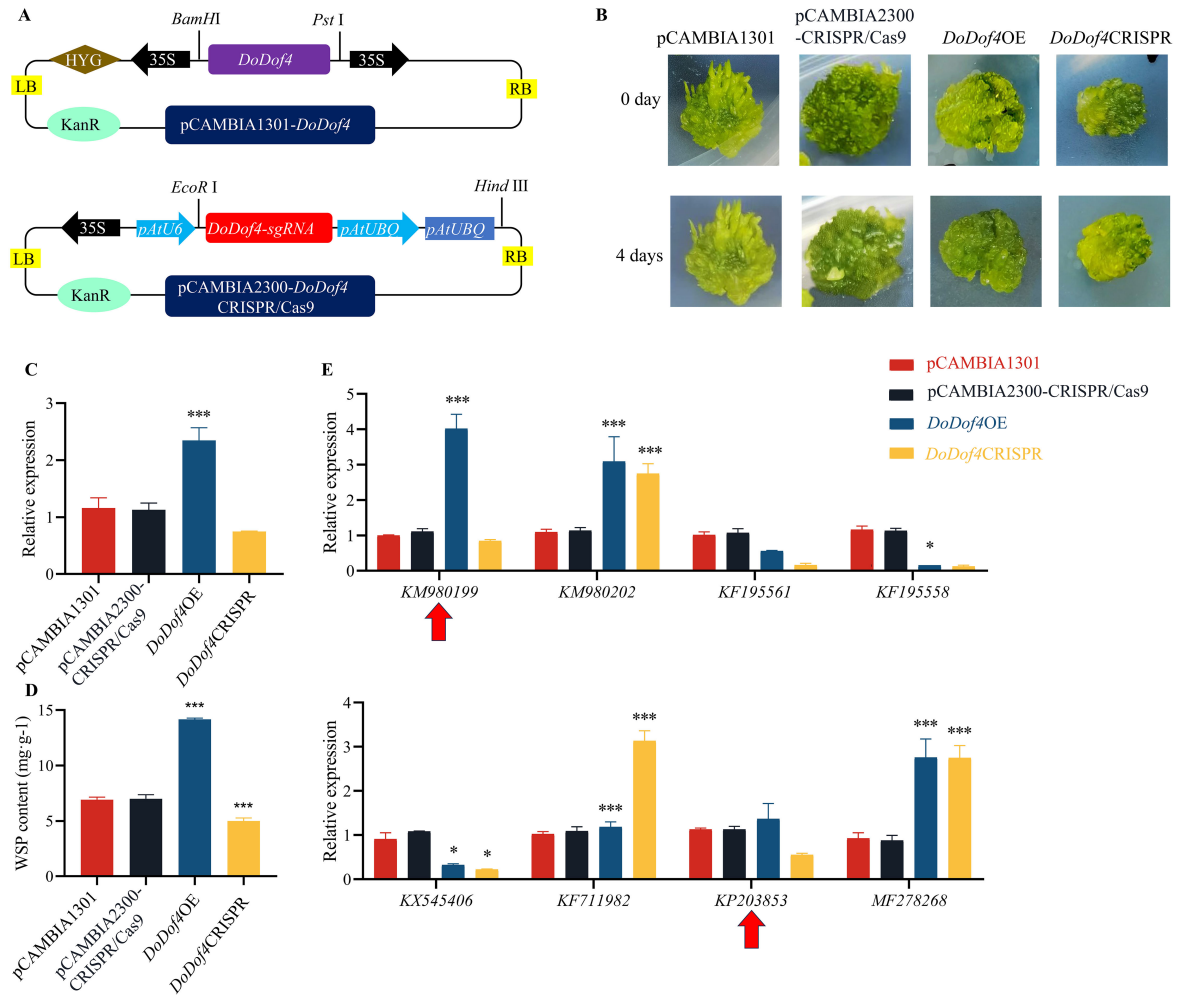
Functional analysis of the DoDof4 gene. **(A)** Diagram of *DoDof4OE* and DoDof4CRISPR vector construction. The coding sequence without the termination codon TGA of *DoDof4* was constructed on pCAMBIA1301 vector. *DoDof4-sgRNA* were ligated to the pCAMBIA2300-CRISPR/Cas9 vector. **(B)** Changes to *D. officinale* PLBs after transformation. **(C)** qRT-PCR analysis of the *DoDof4* gene. **(D)** Analysis of water-soluble polysaccharide content in transgenic PLBs. **(E)** qRT-PCR analysis of *DoDof4* and enzyme-encoding genes. Red arrows (KM980199 and KP203853) indicate potential targets of *DoDof4* to regulate WSP production in *D. officinale*. Bar indicates the mean ± standard deviation (SD) of three replicates (n = 10). Significant differences between groups: * (P < 0.05), and *** (*P* < 0.001).

Plasmids (pCAMBIA1301, pCAMBIA2300-CRISPR/Cas9, *DoDof4*OE, and *DoDof4*CRISPR) were then separately transformed into the D. officinale PLBs (**Figure 9A**). After 4 d of transient transformation, *DoDof4*OE transgenic lines were greener than other lines (**Figure 9B**). After 30d, RNA was extracted from the transgenic lines, and their cDNA was reverse transcribed. The qRT-PCR results showed that the relative expression of the *DoDof4*OE line was higher than that of the pCAMBIA1301 line (**Figure 9C**). Sequencing results revealed a 3 bp deletion in DoDof4CRISPR (**Supplementary Figure S5**). These results validated successful genetic transformation.

### 3.11 *DoDof4* enhances WSP content in *D. officinale*

WSP content in the *DoDof4*OE transgenic line was 1.5-fold higher than that in the pCAMBIA1301 transgenic lines (**Figure 9D**). HPLC detection revealed that the WSPs in *D. officinale* were predominantly composed of glucose and mannose. The contents of glucose and mannose in *DoDof4*OE showed a sharp increase relative to pCAMBIA1301. Furthermore, the mannose/glucose ratio in *DoDof4*OE was 1.87-fold higher than that in pCAMBIA1301 and the mannose/glucose ratio in *DoDof4*CRISPR was the lowest. These results imply that *DoDof4* may significantly stimulate the biosynthesis of WSPs in *D. officinale*, possibly by regulating mannose transportation (**Supplementary Figure S6**). Therefore, the relative expression of KM980199, KM980202, KF195561, KF195558, KX545406, KF711982, KP203853, and MF278268 were characterized in pCAMBIA1301 and *DoDof4*OE transgenic PLBs to explore how *DoDof4* regulates the biosynthesis of WSPs. The relative expression of KM980199 and KP203853 increased in the *DoDof4*OE line (**Figure 9E**), indicating that *DoDof4* may regulate WSP biosynthesis by converting mannose-1P to GDP-mannose.

## 4 Discussion

Bioactive polysaccharides in plants have a wide range of nutritional and health functions, as well as medicinal value. Although most research has focused on extraction, purification, biological activity and structural characterization of WSPs, there are a few studies on their biosynthetic mechanism. The medicinal orchid has a rich content of WSPs, mainly in its stems. The concentration and induction period of MeJA treatment are important for the production of specialized metabolites. Previously, *D. officinale* seedlings treated with 0.1 mM MeJA markedly stimulated the accumulation of WSPs (Yuan et al., 2017), but the time-response relationship is unclear. To better understand the mechanism of *Dendrobium* responding to MeJA, we used *D. officinale* as model, treating them with 0.1 mM MeJA for 30 to 90 d. After stimulation with MeJA, the content of WSPs increased, even more over time. WRKY, bZIP, AP2/ERF, MYB, and bHLH are shown to be associated with resistance to various abiotic stresses (He et al., 2017; Zeng et al., 2021; Li et al., 2022). Dof members accounted for 4% of all DEGs and their homologous genes exhibited a link to secondary metabolism. For example, *ZmDof36* can specially bind to genes involved in starch synthesis at downstream promoters such as Alkylglycerone Phosphate Synthase 1a (*ZmAGPS1a*), *ZmISA3*, and *ZmGBSSI*, to positively up-regulate starch biosynthesis (Wu et al., 2019). However, *Actinidia deliciosa* Dof3 transactivated the starch degradation gene *AdBAM3* (β-amylase 3), affecting fruit flavor (Zhang et al., 2018). In addition, overexpression *CrDof* assisted the intracellular lipid content of *Chlamydomonas reinhardtii* and may regulate the expression of crucial genes such as *CrBCC1*, *CrFAT1*, and *CrSQD1* (Jia et al., 2019). Moreover, in *Fragaria ananassa*, *FaDof2* positively controlled the expression of *FaEGS2* and *FaEOBII*, which are involved in eugenol synthesis, thereby regulating the volatile phenylpropanoid pathway (Molina-Hidalgo et al., 2017). The promoter regions of enzyme-encoding genes involved in WSP biosynthesis contained many Dof binding sites (**Supplementary Figure S7**), such as [T/A]AAAG or their complementary sequences CTTT[T/A]. Furthermore, the enzyme-encoding genes involved in the metabolic pathway of WSPs were significantly (*P* > 0.8) associated with the Dof genes (**Figure 3C**). However, the regulatory mechanism by which Dof modulates the accumulation of WSPs remains unclear in *Dendrobium* species.

To investigate the distribution and functional of Dof genes to regulate WSPs in *Dendrobium* species, we identified 29 Dof members in *D. huoshanense* and *D. nobile*, along with 22 Dof members in *D. officinale.* These were less than the number of *Dof* genes in *A. thaliana* (*n* = 36) (Xu et al., 2020), *Capsicum annuum* (*n* = 33) (Wu et al., 2016), and *Brassica rapa* (*n* = 76) (Ma et al., 2015), but similar to the number in *Cyperus esculentus* (*n* = 29) (Fu et al., 2024). Although three *Dendrobium* species had conserved chromosome numbers (*n* = 19), the *D. huoshanense*, *D. nobile D. officinale* showed species-specific Dof genes localization patterns. *DhDof* and *DnDof* genes were unevenly distributed on 15 chromosomes, while DoDof genes only localized to just 13 chromosomes. These studies indicate similar results not only in the *Dof* gene family, but also in other gene families. During evolution, plants’ chromosomes undergo changes such as fusion and cleavage, resulting in processes such as gene recombination, transfer, and elimination, prompting uneven gene distribution. This phenomenon is prevalent among plants, driving them towards optimal evolution. The collinearity between *D. officinale* and *A. thaliana* (eight pairs) was less than that between *D. officinale* and *O. sativa* (25 pairs), which is consistent with the evolutionary pattern of dicotyledonous plants (Fu et al., 2024). Compared with *D. hosannae*, *D. officinale* and *D. nobile* displayed higher collinearity, indicating a closer evolutionary relationship between *D. officinale* and *D. nobile*.

*Dof* genes were categorized into 4 groups based on the phylogenetic analysis, which was consistent with previous reports (Moreno-Risueno et al., 2007). The expression profiles of 22 *DoDof* genes in flower, leaf, root, and stem were analyzed. Among them, five *DoDof* genes (*DoDof2*/*4*/*6*/*8*/*12*) were highly expressed in stem, suggesting their role in WSP biosynthesis. High expression of *DoDof4*/*6*/*7*/*8*/*14*/*17*/*19* in root, and of *DoDof12* and *DoDof14* in leaf, may be related to resistance to abiotic stresses. After analysis of transcriptomic data, *Dof* genes in the MeJA treatment were screened. qRT-PCR analysis demonstrated that *DoDof1*/*2*/*3*/*4*/*5*/*6*/*8*/*11*/*16*/*17*/*18*/*20*/*21*/*22* were strongly up-regulated, *DoDof9* was unchanged, and the 7 genes (*DoDof7*/*10*/*12*/*13*/*14*/*15*/*19*) were down-regulated. Among the *DoDof* genes that were highly expressed in stem, *DoDof4* displayed the highest expression. WSP was higher in *DoDof4*OE lines than in pCAMBIA1301 lines (**Figure 9D**). Additionally, *DoDof4*OE exhibited significantly increased mannose and glucose contents, along with a higher mannose/glucose ratio (**Supplementary Figure S6**). These results indicate that *DoDof4* is a positive regulator of WSP accumulation, particularly in the transportation of mannose. Conversely, *ZmDof36* decreased WSP content in the seed endosperm, and ultimately contributed to the regulation of carbohydrate metabolism. *SRF1* (a Dof gene) in sweet potato modulated polysaccharide metabolism in storage roots, reducing the glucose and fructose contents (Tanaka et al., 2009). In *O. sativa*, *OsDof11* promoted the expression of *SUCROSE TRANSPORTER 1* (*OsSUT1*), *SUGAR WILL EVENTUALLY BE EXPORTED TRANSPORTER 11* (*OsSWEET11*), and *OsSWEET14*, thereby regulating sugar transport (Wu et al., 2018). Based on the above-mentioned evidence, we propose that *D. officinale* may have evolved a specialized mechanism to biosynthesize and accumulate WSPs as a way to adapt to extreme environments. Furthermore, two polysaccharide biosynthetic genes (KM980199 and KP203853) were potential targets of nucleus-localized *DoDof4* to regulate WSP production in *D. officinale. DoDof4* might combine *DoCSLA* (KM980199) and *DoGMP* (KP203853) genes to positively regulate the metabolic pathway of *D. officinale* WSPs.

## 5 Conclusion

This study aimed to appreciate details about the genetic regulation of the WSPs metabolic pathway in *Dendrobium* species by MeJA. *DhDof*, *DnDof* and *DoDof* genes whose physicochemical properties, chromosome localization, collinearity was analyzed. *DoDof* genes were expressed in flower, stem, leaf, and root. WSP content in transgenic *DoDof4* plants was significantly correlated with *DoCSLA* (KM980199) and *DoGMP* (KP203853), two genes related to the WSP biosynthetic pathway. *DoDof4* might play a role in the WSPs metabolic pathway. This study lays a foundation for exploring additional functions of the *Dof* genes to better understand the molecular mechanism of the WSPs metabolic pathway from the perspective of transcriptional regulation, and fortify cultivation practices of *Dendrobium* species.

## Data Availability

The original contributions presented in the study are publicly available. This data can be found here: China National Center for Bioinformation (CNCB, https://www.cncb.ac.cn/) under BioProject accession number PRJCA043716.
