# Supplementary material for: Comprehensive analysis of Dof transcription factors in Dendrobium species and functional characterization of DoDof4 in the accumulation of water-soluble polysaccharides
Source: Front Plant Sci. 2025 Aug 7;16:1617856. doi: 10.3389/fpls.2025.1617856 (PMC12367786; doi:10.3389/fpls.2025.1617856)
Supplement: Supplementary file 1 [file DataSheet1.pdf]

Supplementary materials

Table legends
Table S1 Protein sequences of 22 DoDof proteins.
Table S2 Protein sequences of 29 Dhof proteins.
Table S3 Protein sequences of 29 Dnof proteins.
Table S4 Dof protein sequences used to construct the phylogenetic tree.
Table S5 Primers used for qRT-PCR analysis of *DoDof* genes.
Table S6 Primers used or cloning *DoDof4*.
Table S7 Physicochemical properties of Dof proteins.
Table S8 Tissue-specific relative expression of *DoDof* genes.

Figure legends
Figure S1 Physicochemical properties analysis of *DhDof*, *DnDof*, *DoDof* genes.
Figure S2 Multiple sequence alignment Dof proteins.
Figure S3 Chromosome localization of *DhDof*, *DnDof*, *DoDof* genes.
Figure S4 Collinearity analysis of *DhDof*, *DnDof*, *DoDof* genes.
Figure S5 Identification of *DoDof4* positive transgenic PLBs.
Figure S6 Quantitative analysis of mannose and glucose content in transgenic PLBs.
Figure S7 Schematic diagram of the promoters of key enzymes related to the biosynthesis of WSPs.

Table S1 Protein sequences of 22 DoDof proteins.

Protein name	Amino acid sequences
DoDof1	MADIGEESPAFKLFGTVIVADENRLKKNSQPPPPPSSSEAAAAELPCPR<br>CRSQETKFCYFNNYNVNQPRHFCKACHRYWTAGGALRNVPVGAGR<br>RRSRFYARTGGETAAEGGRVDFTAEVERWLLRREQMPAAGGKLSNAA<br>SESC
DoDof2	MDAAQWSKEAAVAETAATTAPAISQRKNRRKNDQKQLNCPRCNSNN<br>TKFCYYNNYSLTQPRYFCKTCRRYWTEGGSLRNIPVGGGSRKNKRSIT<br>ATSSSTPSSSSMQNHPKFSSTDQVRDLNLGFQQGGGSGLPELGILRGG<br>GMSGRQMGSNFMHEVFGLQELGLGFQVGAGGATDHDEGRVLFSSSA<br>PRDGILGQNRGLGGETQGFWNGMMGGGGSW
DoDof3	MKKMFISFHSPMRRLLLALHHIKWQQEEILLARSLWWYLWQKGPDL<br>QNSPRLRPPLKCPRCDSTNTKFCYFNNYSLTQPRHFCKTCRRYWTRG<br>GALRSVPVGGGCRRNKRGNKSSSCKPSAPAPITPSASTSSAAAGVFRT<br>APPQFLAPWHHLPDYGFPHGIQQPIDHTMQENMGLEQWRIQQFPFLG<br>GLVEPSLPTAAMVPGLYPFEGELGGGHGKIMSTVKMEESSQAAAAAA<br>ATAATTAANMPRQFFNVGRNDGQIWPGGGDSGSGEGHGGRWISGEH<br>LSGFSSSSTGNLL
DoDof4	MEISTGHHHQAMANHQLDHEVLNCPKSSSSTSPAHHLQQEIKKPRPQ<br>PEQALRCPRCDSTNTKFCYYNNYSLSQPRYFCKGCRRYWTKGGSLRN<br>VPVGGGCRKNKRSSPSTSNSSSSSKKPALQDNQDQVNPTSLLIPSMPLP<br>PPLTYDPSDLTLAFSSFHSHDPFLLGNPNPNPNPNPNGAAFLDILRGGD<br>HSQNHGGGGGGLHSFYYGFSGGGDDIKGMLSFDGGVGGCSDSTTVA<br>AETGAVVGNSDHPASNAHSTSTSHGSLGMESGRDYWNGVPSNSSWH<br>GLINSSLL
DoDof5	MGLSSNQVSVDIHHWPQGGLEFLKSEGREEKQNAPLLMCPRCESTNT<br>KFCYYNNYSRSQPRHFCRACRRHWTEGGTLRNIPVGGGRKNKRRKIT<br>PTATSLAAAAGGNPIAPVVGEMKDPIFPDILRQVLLHQPPPPPLPLESPC<br>MDTLEGLFGASLQKTFLTFMEDFSGVSSGLALSAGEIPFMAAASTSHQ<br>NLSGVQDVSGDGFLGMMDASAASIWVKKETVFPASYWDFWDGGAE<br>DLNIGAVAEEIVAETLLQE
DoDof6	MQGTTMTAFMASRPPLTEPEQNLPCPRCESTNTKFCYYNNYNLSQPR<br>HFCKDCRRYWTKGGTLRNVPVGGGTRKNSKRSLCSSGAASPSASGG<br>VNSKRPSPSSSAGEVKNSELFSSSIPTVDNDHRMLDMTGSFSSLLSSTA<br>QFGNFFECFQSLDSLPILKGVTRPMREPEAQSPAESSGNESAAAAPPPV<br>MPENFLSLPGDTSSWAGGWPDLSIYTPGRNFQ
DoDof7	MAESRDPAIKLFGKTIQIPVGVVVSVTIGEEFIGDDEKAPSDKEASPEQ<br>MDNNEEAPISREEEKKNDESNNLTNEEKIDQNSSSPENSNSKADDEQN<br>ESNNNPLDKANKKPDKILPCPRCKSMDTKFCYYNNYNVNQPRHFCK<br>NCQRYWTAGGSMRNVPVGAGRRKSKSSASHQHFRQITIADCVQAVPE<br>SIHHLQPLKSNGTVLSFGSDAPLCESMASVMKLVEKTVQNRNQKEKE<br>QLSGSPNSNEQVKASSTSNCYGFPPNVSNINGSPWPYTWTHPPFAFPF<br>YPITAYWGMPWLSPPLSTSPSSGSSCSVSNSPNLGKHSREGNLLNHSH

	LDKGNTSIPEKSLWAPKTMRVDVPEKLQSVQCGQPLDSTLIKADLISR
	GGGLFKAFQSNGDHKNPSNELSQVMLANPAALSRSFTFQESS
DoDof8	MDAQWTTQGIGVVKEVEISNTRQPQLQAEKRVRPYKEQSLNCPRCNS
	TNTKFCYYNNYSLSQPRYFCKTCRRYWTEGGSLRNVPVGGGSRKNK
	RSSLANNSSSNPTTTTTSLVSLTSPNLQPTEPIPTVQIPIASSKFYQGSGQ
	DLNLSFQNQSLAHHDQLGAASGSISAMELLRSGISAKGFAPFPAAPGS
	VFGSGFGFQELMRQSNLKLPLDHHGRFSGSMHQMQEGGSGRLLFPFE
	DLKQVHVNNGGHEEENNGVVQEQGDPGLFWNGANGRGGGTW
DoDof9	MFPIYSFHPLPTTDRRWRPKVEEVAPNCPRCDSPNTKFCYYNNYCLAQ
	PRYLCKGCRRYWTKGGSLRSIPVGGGCRKTRRGKSAKFISTLSSSLPSP
	SNCFRPDQALENMSGRIGGNFLMSDQYLDQLPELPQQEALPKPMMTQ
	FDGGSREDIRAEEVENNNFLHYEVMEKEERGAEGVMQRNNDCFLGS
	SNYGFKMDLMRESSRFQGIGISEMTNLEAFL
DoDof10	MMASDSKAAATKSSGCGGGGTGASAGLRLPEQGLKCPRCDSPNTKF
	CYYNNYSLTQPRHFCKSCRRYWTKGGALRNVPVGGGCRKNRKSKSS
	SSSASSSSSSLLPLNPEPGKALTQTAMDLFLPFSYGANTAASMAGFSYN
	GRNSNYGSIASSIESLSSINQDLHWRLQQQRMQFLFGGELAQKDNIVS
	FMNSQVVEPNCFGMAENNKGEIFGDSSGANGSRKGSETGTAWFLDGS
	FAMPAPSPINTDVDMNNPNSVSDWNGISGWSDVHNFTALP
DoDof11	MAHPAALYITITRRQPFSPISLLAGKLSMADLDMESSGDSPVLKLFGAV
	IVGAEDESRLHKSSSASAAAAEEDTAEAAAETLPCPRCKSRETKFCYF
	NNYNVNQPRHFCKACHRYWTAGGVLRNVPVGAGRRRNPLSPRRKIIS
DoDof12	MQDFQSITGQSTGRIFGGVTDGAGGGWDNRRLRAYPSATMAPSPLHP
	PPPLKCPRCESQNTKFCYYNNYNLSQPRHFCKSCRRYWTKGGVLRNV
	PVGGGCRKPKRSSNSKSRRSSASRSSSDSSGRNPADASAALPDSVVFP
	NSSSSNPNPPNHPSFGQPFAAAPNRAAEIFPDGAGTSFSNLMAVTSSQS
	IHGFNFVNPPLLRHPDPHQKLEDITSQDIIHQVVPGRANNSAIDWPPAM
	DTSLYDLAGATDPTAYWSQNHWVDGDPSLYLP
DoDof13	MVAKLDSGTAAAQQNAGSPPLNCPRCDSTNTKFCYYNNYSLAQPRH
	FCKSCKRYWTRGGTLRNVPVGGGCRKNKRTKKSHRSAPSITAAAPSL
	PPPPVIHSPATDLSSLLYGFPQMMNSSTVFFPKFELDAQFAGYEENFQS
	CLSAANIVDLQKSAAAPADFLGDYSLLNSHIPSSSSPMAAEAYQPLLS
	YDDINMGTENNTSNRSINWQDTKGNPLEATNSFAASGGSGAVAQTPY
	LYWNQAMCSGWPESSSSVAPLI
DoDof14	MLPLKVGEKDTCKEKANTKVDAMLDAPENLDQNESLSSSGLCHGIEE
	NQKTATEIAKDAEEPKSEQDNKESGTSGEDKVLKKPDKVLQCPRCNS
	MDTKFCYYNNYNVNQPRHFCKNCQRYWTAGGTMRNVPVGAGRRK
	NKHSSSTYRHTVVMPDALASVQVDGSDLSHHEASPCGSSTSTRLPLK
	CNGTVLKFGPEAPLCESMTSVLKLGDQKRASELGFTTHRENKEEPSCS
	SSAKVSKHAENGFPEDAEQKALHGYQNGLAPLHHLQCYPVSPWAYA
	WNPGWSNIAAAAAMAAAGRSPEPLPRQENGIPTPISWSRPPMVTAPF
	CAPTFPFPLVPTSFWSCIPSWTGGPWNLPLVGNNNRLSPSSSTSNSNCS
	GNGSPTLGKHCRDATSIGEENTEKSLWVPKTLRIDDIDEAAKSSIFATL
	GIKLDEGCLFKAFPSKSQKGEAADAAQLLHANPAAFSRSQSFQETTT

DoDof15	MEITNGHQYQTMTSYPLNEVAPVLSCLKTAASSPYQLHQGINPSAQFQ<br>FQSESQQALKCPRCDSLNTKFCYYNNYSLSQPRYFCKSCRRYWTKGG<br>SLRNIPIGGGCRKNKKPASSSTSPSSSSKKIPLTQVNPNSNLDLGHGFLD<br>VLRSGFVGSANTTTTAIGGIQSHCYGFGGDNGVTLPFDGGLISVSNSTT<br>AAANASAGASTIRHENCWSGFFNGYSNEIHDGHF
DoDof16	MVFSSLPIYLDPPNWSQQTSHQPPSTTTISHPPQLSSLMADPPPHPIGLP<br>GPTRPVSMAERARLAKIPQPEPALKCPRCDSTNTKFCYFNNYSLSQPR<br>HFCKTCRRYWTRGGALRNVPVGGGCRRNKRTKAGSTSSKTSFSAGA<br>SSSTATASMNSTTILPPPPPPQLPFMTHFQPLSDYSTTNIGLGFTGIHPLD<br>SVDYQIGSSGGGGVGLEQWRMQQMQQFPFMGGLDASSTAPATVAGL<br>FPFDGESSGNDHQGYSTGRMLTKASSSGIISQLASVKMEDSSHGINLQ<br>RQYLGVTGNDQYWDGNGDGGASVGNGGGSGWLGDLSGFNSSSSGN<br>IL
DoDof17	MDTVQWPQQGIGVVKGSVVETNQRQAQIQVERSRVRPHKEQSLNCP<br>RCNSTNTKFCYYNNYSLTQPRYFCKTCRRYWTEGGSLRNVPVGGGSR<br>KNKRSSSSSSSCSSSTSSNSTPTPTLTSTTPTTTLPSSSSTNLHPSEHMIN<br>HSLGSSKFYQGGSVHDLNLSFPNQSLGGHELGGSMSALELLRNGISAR<br>GLGPFVPAAMPPGPTSLFGAGFGFQELMRPASNSMKLHPLDGVGGSS<br>GYGGSMQEEGDGRLLFPFEDLKQVSMSNGSHEGVVQGHGDPTLFWN<br>GIMGSGGGGGSGNGGGGGAGGGASW
DoDof18	MVFPSLPLYLDPPNSWNQPPPTHQPVGAGATTSDEHQPPPPAATTGTG<br>TSRPGSMAERARLAKIPQPEAALSCPRCDSNNTKFCYFNNYSLSQPRH<br>FCKTCRRYWTRGGALRNVPVGGGCRRNKRGGKSSSLTSTSTQPKLQP<br>SKSPTSTTATSAPPANLVLLSAAPPLSLLAAAPWNQLQEYGYAGQGLE<br>QWRLPYLGGMEVPAAAGMQVPAPAAAAVTAGMYGYHEGEWSVGG<br>GINGNVGGWMSNSITDHHALGFSGSSSAGNGNL
DoDof19	MSEGRDPAIKLFGRKIPLQEGQTRMDEGDVADSGSAAEPPPPPLEEKD<br>TCKEKAKIEVANKLGAPGELDPKKALDSSVLNNSSVVVEESSTESAKP<br>VEETQPEQDHKEADIAGQDKLLKKPDKLLPCPRCNSMDTKFCYYNN<br>YNVNQPRHFCKNCQRYWTAGGSMRNVPVGAGRRKNKHTGSGYRHT<br>VITPDSLPSVQVDGPDLIDPKPLSPFKVNGTILKFGPEAPLCDSMASILN<br>LGEQNLASQLDFTTGAENREETSCSSACKPKENGFPEKTQQNPMNSY<br>CNGITPFPQIQYFPGPPWASHWNPGWNNFAAATAATGCSPELLPVQDN<br>GILWHQPALITSSFCPHPPLPFPFISPSFWGWTGGPWNLPWLGSSNGVS<br>LSSSKSTSYCSGNDSPTLGKHSREKKTEKSLWIPKTLRIDDPDEAAKSS<br>IFATLGIKHDEVSKFKAFSSSTGNGKGDSSKTALALHANPAALSRSRSF<br>QEIIS
DoDof20	MAANIYPNVHSTATAAIAGGTPALQNSATPHLKCPRCDSTNTKFCYYN<br>NYSLAQPRHFCRTCKRYWTRGGTLRDVPIGGGCRKHKRHNKPKSAIV<br>AALPNSPTLRQLANPLRPPAADLSSLILGMPPATSSNRFFPMFEEAIQSD<br>IEAHHINTLQQSAEAVKSCQAPLICYDDLPTAICEELNLIKENPTEGIYS<br>FNGSSSAAAESAYLYAWPESCSSVVRLL
DoDof21	MEQRHHQLDKQGSRNMASESKSTSEPPRNCPRCESTNTKFCYYNNYS<br>SSQPRYFCRACRRYWTQGGALRNLPHGSSATRRVAKHSKRPSPTSYYS

	VAVSSTSVTPLYHQMNTGTVEPPRCFSGGRLPRQLSMGSQTIDPSMVVGVRIPPAQPEMAGAVSTVAGEEGDRILGNLFGGFEDGSGGEGAVKKMPSAASGPFI
DoDof22	MEISNANEQQVISSHGLEELFIACRKPPAPALEKKTRTQPEQALRCPRCDSTNTKFCYYNNYSLSQPRYFCKGCRRYWTKGGSLRNVPVGGGCRKNKRSSSSSSSLSSSSSSSSSNSIIPSSEKPQDDQTSFISYSHGELTLAFSRQVWPSFADHERFFLGNPNPNSGNSGGYLNFMRNGFLETSNINGYHQINYGNKGSVVEEVEGNNPNTTERSCKMLMDLQMPNGVDGSLGMEYSSWHGIINSSLI

Table S2 Protein sequences of 29 DhDof proteins.

Protein name	Amino acid sequences
DhDof1	MADVDMGGGDKAPGFIKLFGTFIVAHKNPSKKRSAATVTAAAVLPCP RCKSRETRFCYFNNYSVNQPRHFCKICHRYWTAGGTLRNGRGRAGG RNRLFERAAGDGGSCDGGCGEVDGYLIIKWKELISAPSYRCNNGAPH PPPSAITGAGHFPHRPFPSSPPLHHPLPASTLPRPQTPLPPPAPSLPSCPSS LDYLLLDICTRFPLSWRPPHRFHLFLLSLYPSFSLSPTAASKLLDVFGKS RNIDLLWNHLLLMTDRCLLSLASLRIAARALADAREISKCVLLFRLHP QFSTVVALNSVVDELCLRKHVDIARYVVAKHRDSIIGNRETYRLLIIGF CRAGDLMEAARVWNLMLEKEIEPEVDAYEEIITTMFKSNQLQEAMRL FKSMRERIFIDLRTSSYRVVIQWTSKIGRVSYAYMLFAEMLKRGMNFD NATIGYLIYGLLAKKRVKEAYKMFKEAEEVDLSLCHGLMKGLLRMK RPAEATEVFREMLRREIEPNMHTYIMLLQGHMGKRGRKGKEELVNFD SIFVGGLVKAGKTLEASKYAERTMRSTVEVPRFDYNKFLHLFSNEEGV EMFQEVGRRLKEVGMVDLGDVLLVYGERMATRERRRAIWRDSKV CEQYSN
DhDof2	MAESRDPTIKLFGKTIQIPVGVVVSVTIGEEFIGDDEKAPSDKEASPEQ MDNNEEAPISREEEKKNDESNNLTNEEKIDQNSSSPENSNSKADDEQN ESNNNPLDKANKKPDKILPCPRCKSMDTKFCYYNNYNVNQPRHFCK NCQRYWTAGGSMRNVPVGAGRRKSKSSASHQHFRQITIADCVQAVPE SIHHLQPLKSNGTVLSFGSDAPLCESMASVMKLVEKTVQNRNQKEKE QLIGSPNSNEQVKASSTSNCYGFPPNVSNVNGSPWPYTWTHPPFAFPF YPITAYWGMPWLSPPLSTSPSSGSSCSVSNSPNLGKHSREGNLLNHSH LDKGNTSIPEKSLWAPKTMRVDVPEKLQSVQCGQPLDSTLIKADLISR GGGLFKAFQSNGDHKNPSNELSQVLLANPAALSRSFTFQESS
DhDof3	MDAQWTTQGIGVVKEVEISNTRQPQLQAEKRVRPYKEQSLNCPRCNS TNTKFCYYNNYSLSQPRYFCKTCRRYWTEGGSLRNVPVGGGSRKNK RSSLANNSSSNPTSTTTSLVSLSSPNLQPTEPIPTVQIPIASSKFYQGSGQ DLNLSFQNQSLAHHDQLGAASGSISAMELLRSGISAKGFAPFPAAPGS VFGSGFGFQELMRQSNLKLPLDHHGRFSGNMHQMQQGGGGRLLFPF EDLKQVHVNNGGHEEEDNGVVQEQEDPGLFWNGGNGRGGGTW
DhDof4	MQGTTMTAFMASRPPLTEPEQNLPCPRCESTNTKFCYYNNYNLSQPR HFCKDCRRYWTKGGTLRNVPVGGGTRKNSKRSLCSSGVASPSASGCI NSKRPSPSSSAGEVKNSELFSSSIPTVENDHRMLDMTGSFSSLLSSTAQ FGNFFECFQSLDSLPILKGVTRPMLEPEAQSPAESSGNESAAAAPPPVM PENFLSLTGDTSSWAGGWPDLSIYTPGRNFQ
DhDof5	MGLSPNQVSVDIHHWPQGGLEFLTPEGREEKQNAPLLLCPRCESTNT KFCYYNNYSRSQPRHFCRACRRHWTEGGTLRNIPVGGGRKNKRRKIT PTATSFAAVAGGNPIAPVVGEMKDPIFPDILRQVLLNQPPPPPPPPLESL CTDTLEGLFGASLPKTFLTFMEDFSGVSSGLALSAGEIPFMAAASTSH QNLSGVQDVSGDGFLGMMDASAASIWVKKETAFPASYWDFWDGGA GDLNIGAVAEEIVVETLLPE
DhDof6	MFSPEDHRMLSYATRSVPVDHRRWKPSVEIAPNCPRCDSSNTKFCYY NNYSLTQPRYFCKGCRRYWTKGGSLRNVPVGGGCRKNRRGKSARLS

	SDSITSSTSSSSSLRIDTTRVLDSGLRPDQALDDMFDTYYPSSDSGGSNI<br>DMAVMYARYLNQVPDKLPVEIDDSFGFIGSVTAELSSSSTPSTDMNCQ<br>TVSQADEGNLGFMTSNCGMEESVVFPRLEYSSSMLPEMNGGDVFSM<br>NWGSVYPNMPWPAQEQNLAVASTRVQQEIGSNHHFQELVVGDWSSM<br>DQSGFEAF
DhDof7	MDAAQWSKEAAVAETAATTAPAISQRKNRRKNDQKQLNCPRCNSNN<br>TKFCYYNNYSLTQPRYFCKTCRRYWTEGGSLRNIPVGGGSRKNKRLIT<br>ATSSSAPSSSSSMQNHPKFSSTDQVRDLNLGFQQGGGSGLPELGILRG<br>GGMSGRQMGSNFMHEVFGLQELGLGFQVGAGGATDHDEGRVLFSSS<br>APRDGVLGQNRGLGGETQGFWNGMMGGGGSW
DhDof8	MIQELLSAVVEERKFSFPGGGRLVSDDAPSPFLYSTSTANTLSPSSSSSTI<br>QPSSSPSPSTASEPPQQKLRCPRCDSSNTKFCYYNNYNLTQPRHFCKTC<br>RRYWTKGGALRNVPIGGGCRKAKPVPIVAGSGKPGGGSKPNKPIPSLD<br>AIARAGFGFEPELFQPSPILWTGPTAPTPPPHTSHLLALLRAGGGAAIRS<br>FEPNPLSSLSSIRIKEEGMMAEPTGVGLGLNGLNLDPLGQFSSAAGMG<br>SLWRNEGQMPYLQLQENGSPFGDAAAAAGIQELYQKLRPQAYCNDQ<br>LSIAMSNGWPAPGCSSLATSATLTSSIGGGGGSSNSAVAGAEPAMGGEF<br>GYWNPAFSSWSDMSSTN
DoDof9	MSSTVASTIDAGAIRRRRPDSPDPEPDPSEPRRERCPRCSSRDTKFCYY<br>NNYNTSQPRHFCKSCRRYWTLGGSLRNVPIGGSSRKRLRPAAAPPPTQ<br>PLSAVPPPPSVEVGFSPLPIGFPPGLGEGIMDKRIGFDLGLGLGIGPGPL<br>LWPLSLFEEDVVGDTWRIDSGGETFGATTAVWKDVGFSAPMDGGAG<br>GVGAPERKVH
DhDof10	MEITNGHQYQTMTSYPLNEVAPVLSCLKAAASSPYQLHQEINPSPQFQ<br>SQSESQQALKCPRCDSLNTKFCYYNNYSLSQPRYFCKSCRRYWTKGG<br>SLRNIPIGGGCRKNKKPASTSTSPSSSSKKIQLTQVNPNSNLDLGHGFL<br>DVLRSGFVGSAYTTTTAIGGIQSHCYGFGGDNGVTLPFDGGLISVSNST<br>TAAANASAGASTIRHENCWSGFFNGFSNEIHDGHF*
DhDof11	MVFSSLPIYLDPPNWSQQTSHQPPSTTTINHPPQLSSLMADPPPHPIGLP<br>GPTRPVSMAERARLAKIPQPEPALKCPRCDSTNTKFCYFNNYSLSQPR<br>HFCKTCRRYWTRGGALRNVPVGGGCRRNKRTKAGSTSSKTSFSAGA<br>SSSTATASMNSTTILPPPPPPQLPFMTHFQPLSDYSTTNIGLGFTGIHPLD<br>SVDYQIGSSGGGGVGLEQWRMQQMQQFPFMGGLDASSTAPATVAGL<br>FPFDGESSGNDHQGYSTGRMLTKASSSGIISQLASVKMEDSSHGINLQ<br>RQYLGVTGNDHYWDGNGDGGASVGNGGGSGWLGDLSGFNSSSSGN<br>IL
DhDof12	MEQRHHQLDKQASRNMSSESKSTSEPPRNCPRCESTNTKFCYYNNYS<br>SSQPRYFCRACRRYWTQGGALRNLPHGSSTTRRVAKHSKRPSSTSYYS<br>VAVSSTSVTPLHHQMSTGAVEPPRCFSGGRLPRQLSMGCQTIDPSMVV<br>GIRIPPAQPEMAGVVSTVAGEEGERILGSLFGGFEDGSGGEGAVKKMP<br>SASSGPFI
DhDof13	MAANIYPNVHSTSTAAIAGGTPALQNSATPHLKCPRCDSTNTKFCYYN<br>NYSLAQPRHFCRTCKRYWTRGGTLRDVPIGGGCRKNKRHNKPKSAIV<br>AALPNSPTLRQLANPQRPPAADLSSLILGMPPATLSNRFFPMFEEAIQS

	GIESHHINTLQQSAEAVKSYQAPLLCYDDLPTAICEGLSLIKENPTEGF<br>YSFNGSSSAAAESAYLYAWPESCSSVVRLL
DhDof14	MSEGRDPAIKLFGRKIPFKEGQTRMDEGDVADSGSAAEAPLPPLEEKD<br>TCKEKAKIEVVNKLGAPGELDPKEALNSSVLNNSSVVVEETSTESAKT<br>VEEPQPEQDHKEADITGQDKLLKKPDKLLPCPRCNSMDTKFCYYNNY<br>NVNQPRHFCKNCQRYWTAGGSMRNVPVGAGRRKNKHTGSGYRHTV<br>ITPDSLPSVQVDVPDLIDPKPFSPFKVNGTILKFGPEAPLCESMSSILNL<br>GEQNLASQLDFTTGAENREETSCSSACKPKENGFPEKTQQNPMNSYS<br>NGITPLPHIQYFPGPPWASHWNPGWNNFAAAMATTGCSPELLHVQDN<br>GILWHQPALITSAFCPPPPLPFPFISPSFWGWTGGPWNLPWLGSSNGVS<br>LSSSKSTSYCSGNGSPTLGKHSREEKTEKSLWIPKTLRIDDPDEAAKSSI<br>FATLGIKHDEVSKFKAFSSSTGNGKGDSSKTALALHANPAALSRSRSF<br>QEIIS
DhDof15	MSEGRDPAIKLFGRKIPFKEGQTRMDQGDVADSGSAAEAPPPPLEEKD<br>TCKEKAKIEVVNKLGAPGELDPKEALNSSVLNNSSVVVEETSTESAKT<br>VEEPQPEQDHKEVDITGQDKLLKKPDKLLPCPRCNSMDTKFCYYNNY<br>NVNQPRHFCKNCQRYWTAGGSMRNVPVGAGRRKNKHTGSGYRHTV<br>ITPDSLPSVQVDVPDLIDPKPFSPFKVNGTILKFGPEAPLCESMSSILNL<br>GEQNLASQLDFTTGAENREETSCSSACKPKENGFPEKTQQNPMNSYS<br>NGITPLPHIQYFPGPPWASHWNPGWNNFAAAMATTGCSPELLHVQDN<br>GILWHQPALITSAFCPPPPLPFPFISPSFWGWTGGPWNLPWLGSSNGVS<br>LSSSKSTSYCSGNGSPTLGKHSREEKTEKSLWIPKTLRIDDPDEAAKSSI<br>FATLGIKHDEVSKFKAFSSSGNGKGDSSKTALALHANPAALSRSRSFQ<br>EIIS
DhDof16	MVFPSLPLYLDPPNSWNQPPPTHQAPVGAGATTSDERQPPPPADATGT<br>GASRPGSMAERARLAKIPQPEAALSCPRCDSNNTKFCYFNNYSLSQPR<br>HFCKTCRRYWTRGGALRNVPVGGGCRRNKRGGKSSSLTSTSTQPKLQ<br>PSKSPTSTTATSAPPANLVLLSAAPPFSLLAAAPWNQLQEYGYAGQGL<br>EQWRLPYMEVPAAAGMQVPAPAAAAVTAGMYGYHEGEWSVGGGIN<br>GNVGGWMSNSITDYHALGFSGSSSAGNGNH
DhDof17	MDPVIKLFGKTIQLPAARQGLAATAGEDRSGYEKKGNHESKRKNEET<br>RFSQQEERIDEKNMSEAKEEKNNPMDKTLKKPDKILPCPRCKSMDTK<br>FCYFNNYNVNQPRHFCKNCQRYWTEGGSMRNVPVGAGRRKSKSSTT<br>NQYYNHHIRIPDCAQADFYESNNQPSPLKHNGTEHSFSAEAPLLESMD<br>SNLENQFSRSLATPWPNPWSQTPLYPMTPYLAMPWLCSCRHSTVPKK<br>LRINEDHDKAAKCSVCKKLGFKNDKTDKIFQTRSAEASQVLCANPAA<br>LSRSLNFQERS
DhDof18	MDTVQWPQQGIGVVKGSVVESNQRQAQIQVERSRVRPHKEQALNCP<br>RCNSTNTKFCYYNNYSLTQPRYFCKTCRRYWTEGGSLRNVPVGGGSR<br>KNKRSSSSSSCSSSTSSNSTPTPTLTSTTPTTTLPSSSSTNLQPSEHMINH<br>SLGSSKFYQGGSVHDLNLSFPNQSLGGHELGGSMSALELLRNGISARG<br>LGPFVPAMPPGPTSLFGAGFGFQELMRPASNSMKLHPLDGVGGSSGY<br>GGSMQEEGDGRLLFPFEDLKQVSMSNGSHEGVVQGHGDPTLFWNGI<br>MGSGGGGGGGNGGGGGAGGGASW

DhDof19	MTLLTINTDTCKEKANTEVDAMLDAPKNLDQKESLSSSGLGHGIEED<br>QKTATELAKDAEEPKSEQDNKESGTSGEDKVLKKPDKVLQCPRCNSM<br>DTKFCYYNNYNVNQPRHFCKNCQRYWTAGGTMRNVPVGAGRRKN<br>KHSSSTYRHTVVTPDALASVQVDGSDLSHHEASPCGSSTTTRLPLKCN<br>GTVLKFGPEAPLCESMTSVLKLGDQKRASELGFTTHRENKEEPSCSSS<br>AKVSKHAENGFPEDAEQKALHGYQNGLAPLHHLQCYPVSPWAYAWN<br>PGWSNIAAAAAMAAAGRSPEPLPRQENGIPTPISWSRPPMVTAPPFCA<br>PTFPFPLVPTSFWSCIPSWTGGPWNLPLVGNNNRLSPSSSTSNSNCSGN<br>GSPTLGKHCRDATSIGEENTEKSLWVPKTLRIDDIDEAAKSSIFATLGIK<br>LDEGCLFKAFSSKSQKGEAADAAQLLHANPAAFSRSQSFQETTT
DhDof20	MVAKLDSGTAAAQQNAGSPPLNCPRCDSTNTKFCYYNNYSLAQPRH<br>FCKSCKRYWTRGGTLRNVPVGGGCRKNKRTKKSHRSAASITAAAPSL<br>PPPPVIHSPATDLSSFLYGFPQMMNSSTVFFPKFELDAQFAGYEENFQS<br>CLSAANIVDLQKSAVAPADFLGDYSLLNSHIPSSSSPMAAEAYQPLLSY<br>DDINMGTENNTSNRSINWQDTKGNPLEATNSFAASGGSGAVAQTPYL<br>YWNQAMSGSWPESSSSVAPLI
DhDof21	METVQWPQQGVERKVRPHREQALNCPRCKSTNTKFCYYNNYSLSQP<br>RYFCKSCRRYWTEGGSLRNVPVGGGSRKNNKRLNSSSSSQASSKITTL<br>ATPTSSTSLLSSTSMNQQPSKLIPLELTSFSLSSSVSALQPIRSGIASRKFI<br>SFEIPNSSKENELSANARIRLRKSTPFQEDLHALSMPTIEDFGLQELMW<br>PSKLKVPLDYGVGSMEEGKERRDGNLPFPFEDLKQVSIRNGCDEN<br>TVEGVDQTWFWEGIMGEGEGRGGW
DhDof22	MQDFQSITGQSTGRIFGGVTDGTGGGWDNRRLRAYPSATMAPSPLHP<br>PPPLKCPRCESQNTKFCYYNNYNLSQPRHFCKSCRRYWTKGGVLRNV<br>PVGGGCRKPKRSSNSKSRRSSASRSSSDSSGRNPADASAALPDSAIFPN<br>SSSSNPNPPNPSFDQPFAAAPDRAAEIFPDGAGTSFSNLMAVTSSQSIH<br>GFNFVNPPLLRNPDPHQKLEDITSQDIIHQVVPGRTNNSSIYWPPAMD<br>TSLYDLAGATDPTAYWSQNHWVDGDPSLYLP
DhDof23	MMASDSKAAATKSSGCGGGGTGASAGLRLPEQGLKCPRCDSPNTKF<br>CYYNNYSLTQPRHFCKSCRRYWTKGGALRNVPVGGGCRKNRKSKSS<br>SSSASSSSSSLLPLNPEPGKALTQTAMDLFLPFSYGANTAASMAGFSYN<br>GRNSNYGSIASSIESLSSINQDLHWRLQQQRMQFLFGGELAQKDNIVS<br>FMNSQVVEPNCFGMAENNKGEIFGDSSGANGSRKGSETGTAWFLDGS<br>FAMPAPSAINTDVDMNNPNSVSDWNGISGWSDVHNFTALP
DhDof24	MVFSSAPLYVDPPNWNQQGSNNIVVDEEDVHQLPQPHEAAASGSAP<br>HQVAAGGSTSRPEPVVVSMAERARLAKIPQVEAALKCPRCDSTNTKF<br>CYFNNYSLTQPRHFCKTCRRYWTRGGALRSVPVGGGCRRNKRGNKS<br>SSCKPSAPAPITPSASTSSAAAGVFRTAPPQFLAPWHHLPDYGFPHGIQ<br>QPIDHTMPENMGLEQWRIQQFPFLGGLVEPSLPTAAMVPGLYPFEGEL<br>GGGHGKIMSTVKMEESSQAAAAAAATAATTAANMPRQFFNVGRNDG<br>QIWPGGGDSGSGEGHGGRWISGEHLSGFSSSSTGNLL
DhDof25	MEGMEIPKASRTQKAPPLKCPRCYSANTKFCYYNNYSLTQPRHFCRT<br>CHRHWTAGGVLRNIPASGSRKNKRQKTTTTTNASTSAAAGHFPAITTP

	AVAENNDRFENLLTRNFRPFEFEFGTVSSGFSFPVNSTSLFPSTDYSFGR<br>SKEGLVMDESKFSTSFSVEESLVLPADFLDFWNGGVDFMAGAY
DhDof26	MEISTGHHHHQAMANHQLDHEVLNCPKSSSSTSPAHHLHQEIKKPRP<br>QPEQALRCPRCDSTNTKFCYYNNYSLSQPRYFCKGCRRYWTKGGSLR<br>NVPVGGGCRKNKRSSPSTSNSSSSSKKPALQDNQDQVNPTSLLIPSMP<br>LPPLTYDPSDLTLAFSSFHSHDPFLLGNPNPNPNPNPNGAAFLDILRGG<br>DHSQNHGGGGGGLHSFYYGFSGGGDDIRGMLSFDGGVGGCSDSTTV<br>AAETGAVVGNSDHPASNAHSTSTSHGSLGMESGRDYWNGVPSNSSW<br>HGLINSSLL
DhDof27	MADIGEEAPAFKLFGTVIVADENHLKKNSQPPPPPSSSAAAAAELPCPR<br>CRSQETKFCYFNNYNVNQPRHFCKACHRYWTAGGALRNVPVGAGR<br>RRSRFYARTGGGTASEDGRVDFNAEVERWLLRREQMPAAGGKLSNA<br>ASESC
DhDof28	MQGTTSASATATKSPFSDPEQNLQCPRCESTNTKFCYFNNYNLSQPRH<br>FCKDCRRYWTRGGALRNVPVGGGTRKNSKRSSATSAVAASSAVNPKR<br>SLPSSSTAEAQRSELLPAPFTPVDDDHRMLDITGSFSSLLSSTNHLGNFL<br>DGEAATPALAESFLGLPVDSSSWGWPDLTIYTPDTRFE
DhDof29	MFPIYSFHPLPTTDRRWRPKVEEVAPNCPRCDSPNTKFCYYNNYCLAQ<br>PRYLCKGCRRYWTKGGSLRSIPVGGGFRKTRRGKSAKFISTLSCSLPSP<br>SNSFRPDQALDNMSGRIGGNFLLPDQYLDQLPELPQQEALPKPMMIQF<br>DGGSREDIRAEEVENNNFLHYEVMEKEERGAEGVMHRNNDSFLGSS<br>NYGFKTDLMWESSRFQGIGISEMTSLEAFL

Table S3 Protein sequences of 29 DnDof proteins.

Protein name	Amino acid sequences
DnDof1	MFPIYSFHPLPTTDRRWRPKVEEVAPNCPRCDSPNTKFCYYNNYCLAQPRYLCKGCRRYWTKGGSLRSIPVGGGCRKTRRGKSAKFISTLSSSLPSPSSSFRPDQALDNMSGRIGGNFLLPDQYLDQLPELPQQEALPKPMMIQFDGGSREDIRAEEVENKNFLHYEVMEKEERGAEGVMHRNNDSFLGSSNYGFKTDLMWESSRFQGIGISEMTSLEAFL
DnDof2	MEISNANEQQVISSHGLEELFIACRKPPAPALEKKSRTQPEQALRCPRCDSSNTKFCYYNNYSLSQPRYFCKGCRRYWTKGGSLRNVPVGGGCRKNKRSSSSSSLSSSSSSSSNSIIPSSEKPQDDQTNFISYSHGELTLAFSRQVWPSFADHERFFLGNPNPNSGSSGGYLNFMRNGFLETSNINEYHKINYGNNGSVVEEVEGNNPNTTERSCKMLMDIQMPNGVDGSLGMEYSSWHGIINSSLI
DnDof3	MASESKSTSEPPRNCPRCESTNTKFCYYNNYSSSQPRYFCRACRRYWTQGGALRNLPHGSSATRRVAKHSKRPSSTSYYSVAVSSTSVTPLYHQMSTGAVEPPRCFSGGRLPRQLSMGSQTIDPSMVVGVRIPPAQPEMGGVVSTVAGEDQGDRILGNLFGGFEDGSGGEGAVKKMPSASSGPFI
DnDof4	MAANIYPTVHSTATAAIAGGTPALQNSATPHLKCPRCDSTNTKFCYYNNYSLAQPRHFCRTCKRYWTRGGTLRDVPIGGGCRKNKRHNKPKSAIVAALPNSPTLRQLANPLRPPAADLSSLIIGMPPATLSNRFVPMFEEAIQSGIESHHINTLQQSAEAVKSYQAPLLCYDDLPTVICEGLSLIKENPTEGIYSFNGSSSAAESAYLYAWPESCSSVVRLL
DnDof5	MSEGRDPAIKLFGRKIPLQEGQTRMDEGDVADSGSAAELPPPPLEEKDTCKEKAKIEVANKLGAPGELDPKKALDSSVLNNSSVVVEGSSTESAKPVEETQPEQDHKDADIAGQDKLLKKPDKLLPCPRCNSMDTKFCYYNNYNVNQPRHFCKNCQRYWTAGGSMRNVPVGAGRRKNKHTGSGYRHTVITPDSLPSVQVDCPDLIDPKPLSPFKVNGTILKFGPEAPLCDSMASILNLGEQNLASQLDFTTGAENREETSCSSACKPKENGFPEKIQQNPMNSYSNGITPLPHIQYFPGPPWASHWNPGWNNFASAMAATGCSPELLPVQDNGILWHQPALITSAFCPPLPFPFISPSFWGWTGGPWNLPWLGSSNGVSLSSSKSTSYCSGDGSPTLGKHSREEKTEKSLWIPKTLRIDDPDGAAKSSIFATLGIKHDEVSKFKAFSSSTGNGKGDSSKTALALHANPAALSRSRSFQEIIS
DnDof6	MVFPSLPLYLDPPNSWNQPPPTHQAPVGAGATTSDERQPPPPADATGTGASRPGSMAERARLAKIPQPEAALSCPRCDSNNTKFCYFNNYSLSQPRHFCKTCRRYWTRGGALRNVPVGGGCRRNKRSGKSSSLTSTSTQPKLQPSKSPTSTTATSAPPANLVLLSAAPPLSLLAAAPWNQLQEYGYAGQGLEQWRLPYLGGMEVPAVAGMQVPAPAAAAVTAGMYGYHEGEWSVGGGINGNVGGWMSNSITDHHALGFSGSSSAGNGNL
DnDof7	MESVRDSSGFKLFGAVIATDKRTLAAAQEGDDVATEAAGEEEVDVAASLPCPRCKSQQTKFCYFNNYNVNQPRHFCKACHRYWTAGGTLRNVPVGAGRRRNRIAPPELR
DnDof8	MESSGDSPVLKLFGAVIVGAEDESRLHKSSSASPAAAEEDTAEAAAETLPCPRCKSRETKFCYFNNYNVNQPRHFCKACHRYWTAGGVLRNVPV

	GAGRRRNPLSPRRKIIS
DnDof9	MASDSKAAATKSSGCGGGGTGASAGLRLPEQGLKCPRCDSPNTKFCY
	YNNYSLTQPRHFCKSCRRYWTKGGALRNVPVGGGCRKNRKSKSSSSS
	ASSSSSSLLPLNPEPGKALTQTAMDLFLPFSYGANTAASMAGFNYNGR
	NSNYGSIASSIESLSSINQDLHWRLQQQRMQFLFGGELAQKDNIVSFM
	NSQVVEPNCFGMAENNKGEIFGDSSGANGSRKGSETGTAWFLDGSFA
	MPAPSAINTDVDMNNPNSVSDWNGISGWSDVHNFTALP
DnDof10	MDAAQWSKEAAVAETAATTAPAISERKNRRKNDEKQLNCPRCNSNNT
	KFCYYNNYSLTQPRYFCKTCRRYWTEGGSLRNIPVGGGSRKNKRSITA
	TSSSAPSSSSSMQNHPKFSSTDQVRDLNLRFQQGGGSGLPELGILRGG
	GMSGRQMGSNFMHEVFGLQELGLGFQVGAGGATDHDEGRVLFRSSA
	PRDGILGQNRGLGGETQGFWNGMMGGGGSW
DnDof11	METVQWPQQVVERKVRPHREQALNCPRCKSTNTKFCYYNNYSLSQP
	RYFCKSCRRYWTEGGSLRNVPVGGGSRKNNKRLNSSSSSQASSKITTL
	ATPTSSTSLLSSTSMNQQPSKLIPLELTSFSLSSSVSALEPIRSGIASRKFI
	SFEIPNSSKENEVSANARIRLRKSTPFQNVHRQEELHALSMPSIEDFGL
	QELMWPSKLKVPLDYGVGSMEEGKERRDGNLPFPFEDLKQVSLRN
	ACDENTVEGVDQTWFWEGIMGEGEGRGGW
DnDof12	MAFSSAPLYVDPPNWNQQGSNNIVVDEEDVHQLPQPHEAAPHQVAA
	GGSTSRPEPVVVSMAERARLAKIPQVEAALKCPRCDSTNTKFCYFNN
	YSLTQPRHFCKTCRRYWTRGGALRSVPVGGGCRRNKRGNKSSSCKPS
	APAPITPSASTSSAAAGVFRTAPPQFLAPWHHLPDYGFPHGIQQPIDHT
	MPENMGLEQWRIQQFPFLGGLVEPSLPTAAMVPGLYPFEGELGGGHG
	KIMSTVKMEESSQAAAAAAATAATTAANMPRQFFNVGRNDGQIWPG
	GGDSGSGEGHGGRWISGEHLSGFSSSSTGNFL
DnDof13	MEISTGHHHQAMANHQLDHGVLNCPKSSSSTSPAHHLQQEIKKPRPQ
	PEQALRCPRCDSTNTKFCYYNNYSLSQPRYFCKGCRRYWTKGGSLRN
	VPVGGGCRKNKRSSPSTSNSSSSSKKPALQDNQDQVNPTSLLIPSMPLP
	PLTYDPSDLTLAFSSFHSHDPFLLGNPNPNPNPNPNGAAFLDILRGGDH
	SQNHGGGGGGLHSFYYGFSGGGDDIRGMLSFDGGVGGCSDSTTVAA
	ETGAVVGNSDHPASNAHSTSTSHGSLGMESGRDYWNGVPSNSSWHG
	LINSSLL
DnDof14	MFSPEDHRMLSYATRSAPVDHRRWKPSVEIAPNCPRCDSSNTKFCYY
	NNYSLTQPRYFCKGCRRYWTKGGSLRNVPVGGGCRKNRRGKSARLS
	SDSITSSTSSSSSLRIDTTRVLDSGLRPDQALDDMFDTYYPSSDSGGSNI
	DMAVMYARYLNQVPDKLPVEIDDSFGFIGSVTAELSSSSTPSTDMNCQ
	TVSQADEGNLGFMASNCGMEESVVFPRLEYSSSMLPEMNGGDVFSM
	NWGSVYPNMPWPAQEQNLAVASTRVQQEIGSNHHFQELVVGDWSSM
	DQSGFEAF
DnDof15	MQDFQSITGQSTGRIFGGVADGTGGGWDNRRLRAYPSATMAPSPLHP
	PPPLKCPRCESQNTKFCYYNNYNLSQPRHFCKSCRRYWTKGGVLRNV
	PVGGGCRKPKRSSNSKSRRSSASRSSSDSSGRNPADASAALPDSAIFPN
	SSSSNPNPPNPSFDQPFAAAPDRAAEIFPDGAGTSFSNLMAVTSSQSIH
	GFNFVNPPLLRNPDPHQKLEDITSQDIIHQVVPGRANNSAIDWPPAMD

	TSLYDLAGATDPTAYWSQNHWVDGDPSLYLP
DnDof16	MIQELLSAVVEERKFSFPGGGRLVSDDAPSPFLYSTSTATTLSPSSSSSTI
	QPSSSPSPSTASEPPQQKLRCPRCDSSNTKFCYYNNYNLTQPRHFCKTC
	RRYWTKGGALRNVPIGGGCRKAKPAPIVAGSGKSGAGSKPNKPIPSLD
	AIARAGFGFEPELFQPSPILWTGPTAPTPPPHTSHLLALLRAGGGAAIRS
	FEPSPLSSLSSIRIKEEGMMAEPTGVGLGLNGLNLDPLGQFSSAAGMG
	SLWRNEGQMPYLQLQENGSPFGDAAAAAGIQELYQKLRPQAYCNDQ
	LSIAMSNGWSAPGCSSLATSATLTSSIGGGGGGSSNSAVAGAEPAMGGEF
	GYWNPAFSSWSDMSSTN
DnDof17	MSSTAASTIDAGAIRRRRPDSPDPEPDPSEPRRERCPRCSSRDTKFCYY
	NNYNTSQPRHFCKSCRRYWTLGGSLRNVPIGGSSRKRLRPAAAPPPTQ
	PLSAVPPPPSVEVGFSPLPIGFPPGLGEGIMDKRIGFDLGLGLGIGPGPL
	LWPLSLFEEDVVGDTWRIESGGETFGATTAVWKDVGFSAPMDGGAG
	GVGAPERKVH
DnDof18	MEITNGHQYQTMISYPLNEVAPVLSCLKAAASSPYHLHQEINPSPQFQ
	SQSESQQALKCPRCDSLNTKFCYYNNYSLSQPRYFCKSCRRYWTKGG
	SLRNIPIGGGCRKNKKPASSSTSPSSSSKKIPLTQVNPNSNLDLGHGFLD
	VLRSGFVGSANTTTTAIGGIQSHCYGFGGDNGVTLPFDGGLISVSDSTT
	AAANASAGASTVRHENCWSGFFNGFSNEIHDGHF
DnDof19	MVFSSLPIYLDPPNWSQQTSHQPPSTTTINHPPQLSSLMADPPPHPIGLP
	GPTRPVSMAERARLAKIPQPEPALKCPRCDSTNTKFCYFNNYSLSQPR
	HFCKTCRRYWTRGGALRNVPVGGGCRRNKRTKAGSTSSKTSFSAGA
	SSSTATASMNSTTILPPPPPPQLPFMTHFQPLSDYSTTNIGLGFTGIHPLD
	SVDYQIGSSGGGGVGLEQWRMQQMQQFPFMGGLDASSTAPATVAGL
	FPFDGESSGNDHQGYSTGRMLTKASSSGIISQLASVKMEDSSHGINLQ
	RQYLGVTGNDQYWDGNGDGGASVGNGGGSGWLGDLSGFNSSSSGN
	IL
DnDof20	MDPVIKLFGKTIKLPAVREGLITTAGEDSSGYEKKFSPMILRASSKKQD
	NIESKRKNEETRFSQQEERIDEKNMSEAKEEKNNPMDKTLKKPDKILP
	CPRCKSMDTKFCYFNNYNVNQPRHFCKNCQRYWTEGGSMRNVPVG
	AGRRKSKSSTTNQYYNHHIRIPDCAQADFYESNNQPSPLKHNGTVLSF
	SAEAPLLESMDSNLENQLSRSLATPWPNPWSQTPLYPMTPYLAMPWL
	CSCRHSTVSGKRSISSGNGDIAKSEKYLWIPKKLRINEGHEKAAKCSV
	WEKLGFKNDKTDKIFQTRSAEASQVLCANPAALSRSLNFQERS
DnDof21	MDTVQWPQQGIGVVKGSVVESNQRQAQIQVERSRVRPHKEQALNCP
	RCNSTNTKFCYYNNYSLTQPRYFCKTCRRYWTEGGSLRNVPVGGGSR
	KNKRSSSSSSCSSSTSSNSTPTPTLTSTTPTTTLPSSSSTNLQPSEHMINH
	SLGSSKFYQGGSVHDLNLSFPNQSLGGHELGGSMSALELLRNGISARG
	LGPFVPAMPPGPTSLFGAGFGFQELMRPASNSMKLHPLDGVGGSSGY
	GGSMQEEGDGRLLFPFEDLKQVSMSNGSHEGVVQGHGDPTLFWNGI
	MGSGGGGNGGGGGAGGGASW
DnDof22	MVAKLDSGTAAAQQNAGSPPLNCPRCDSTNTKFCYYNNYSLAQPRH
	FCKSCKRYWTRGGTLRNVPVGGGCRKNKRTKKSHGSAASITAAAPSL
	PPPPVIHSPATDLSSLLYGFPQMMNSSTVFFPKFELDAQFAGYEENFQS

	CLSAANIVDLQKSVAAPADFLGDYSLLNSHIPSSSSPMAAEAYQPLLSY
	DDINMGTENNTSNRSINWQDTKGKPLEATNSFAASGGSGAVAQTPYL
	YWNQAMSGSWPESSSSVAPLI
DnDof23	MARVDSGGMSEVRDPAIKLFGRKIPLLEEQRLAEEGGVAALAQAMEP
	LKVGEKDTCKEKANTEVDAMLDALKNLDQKESLSSSGLGHGIEEDQ
	KTATELAKDAEEPKSEQDNKESGTSGEDKVLKKPDKVLQCPRCNSMD
	TKFCYYNNYNVNQPRHFCKNCQRYWTSGGTMRNVPVGAGRRKNKN
	SSSTYRHTVVTPDALASVQIDGSDLSHHEASPCGSSTTTRLPLKCNGT
	VLKFGPEAPLCESMTSVLKLGDQKRASELGFTTHRENKEEPSCSSSAK
	VSKHAENGFPEDAEQKALHGYQNGLAPLHHLQCYPVSPWAYAWNPG
	WSNIAAAATMAAVGRSPEPLPRQENGIPTPISWSRPPMVTAPPFCAPTF
	PFPLVPTSFWSCIPSWTGGPWNLPLVGNNNRLSPSSSTSNSNCSGNGSP
	TLGKHCRDATSIGEENTEKSLWVPKTLRIDDIDEAAKSSIFATLGIKLDE
	GCLFKAFPSKSQKGEAADAAQLLHANPAAFSRSQSFQETTT
DnDof24	MDAQWTTQGIGVVKEVEISNTRQPQLQAEKRVRPYKEQSLNCPRCNS
	TNTKFCYYNNYSLSQPRYFCKTCRRYWTEGGSLRNVPVGGGSRKNK
	RSSLANNSSSNPTTTTTSLVSLSSPNLQPTEPIPTVQIPIASSKFYQGSGQ
	DLNLSFQNQSLAHHDQLGAASGSISAMELLRSGISAKGFAPFPAAPGS
	VFGSGFGFQELMRQSNLKLPLDHHGRFSGSMHQMQQGGGGRLLFPF
	EDLKQVHVNNGGHEEEDNGVVQEQEDPGLFWNGGNGRGGGTW
DnDof25	MAESRDPAIKLFGKTIQIPVGVVVSVTIGEAFIGDDEKAPSDKEASPEQ
	MDNNEEAPISREEEKKNDESNNLTNEEKIDQNSSSPENSKADDEQNES
	NNNPQDKANKKPDKILPCPRCKSMDTKFCYYNNYNVNQPRHFCKNC
	QRYWTAGGSMRNVPVGAGRRKSKSSASHQHFRQITIADCVQAVPESI
	HHLQPLKSNGTVLSFGSDAPLCESMASVMKLVEKTVQNRNQKEKEQ
	LSGSPNSNEQVKASSTSNCYGFPPNVSNINGSPWPYTWTHPPFAFPFYP
	ITAYWGMPWLSPPLSTSPSSGSSCSVSNSPNLGKHSREGNLLNHSHLD
	KGNTSIPEKSLWAPKTMRVDVPEKMQSVQCGQPLDSTLIKADLISRGG
	GLFKAFQSNGDHKNPSNELSQVLLANPAALSRSFTFQESS
DnDof26	MQGTTSASATATKSPFSDPEQNLQCPRCESTNTKFCYFNNYNLSQPRH
	FCKDCRRYWTRGGALRNVPVGGGTRKNSKRSSATSAVAATSAVNPKR
	PLPSSSTGEAQRSELLPAPFTPVDDDHRMLDITGSFSSLLSSTNHLGNF
	LDGEAATPALAESFLGLPVDSSSWGWPDLTIYTPDTRFE
DnDof27	MADIGEESPAFKLFGTVIVADENRLKKNSQPPPPPSSSAAAEAELPCPR
	CRSQETKFCYFNNYNVNQPRHFCKACHRYWTAGGALRNVPVGAGR
	RRSRFYARTGGGTASEDGRADFTADVERWLLRREQMPAAGGKLSNA
	ASESC
DnDof28	MQGTTMTAFMASRPPLTEPEQNLPCPRCESTNTKFCYYNNYNLSQPR
	HFCKDCRRYWTKGGTLRNVPVGGGTRKNSKRSLCSSGAASPSASGG
	VNSKRPSPSSSAGEVKNSELFSSSIPTVDNDHRMLDMTGSFSSLLSSTA
	QFGNFFECFQSLDSLPILKGVTRPMLEPEVQSPAESSGNESAAAARPPV
	MPENFLSLPGDTSSWAGGWPDLSIYTPGRNFQ
DnDof29	MGLSSNQVSVDIHHWPQGGLEFLKSEGRDEKQNAPLLMCPRCESTNT
	KFCYYNNYSRSQPRHFCRACRRHWTEGGTLRNIPVGGGRKNKRRKIT

	PTATSFAAVAGGNPIAPVVGEMKDLIFSDILRQVRLNQPPPPPLESPCM DTMEGLFGASLQKTFLTFMEDFSGVSSGLALSAGEIPFMAAASTSHQN LSGVQDVSGGCGDGFFGMMDASAASIWVKKETVFPASYWDFWDGG AEDLNIGAVAEEIVTETLLPE

Table S4 Dof protein sequences used to construct the phylogenetic tree.

Plant name	Dof protein sequences
Arabidopsis	AtDof1
thaliana	MGGSMAERARQANIPPLAGPLKCPRCDSSNTKFCYYNNYNLTQPRHFC
	KGCRRYWTQGGALRNVPVGGGCRRNNKKGKNGNLKSSSSSSKQSSSV
	NAQSPSSGQLRTNHQFPFSPTLYNLTQLGGIGLNLAATNGNNQAHQIGS
	SLMMSDLGFLHGRNTSTPMTGNIHENNNNNNNENNLMASVGSLSPFA
	LFDPTTGLYAFQNDGNIGNNVGISGSSTSMVDSRVYQTPPVKMEEQPNL
	ANLSRPVSGLTSPGNQTNQYFWPGSDFSGPSNDLL
	AtDof2
	MLPYIGHNSYQQHQFPLPEMEIPEKWKLSYEQEAITAPACPRCASSNTK
	CYYNNYSLSQPRYFCKGCRRYWTKGGSLRNIPVGGGCRKRSRSRQNS
	HKRFGRNENRPDGLINQDDGFQSSPPGSDIDLAAVFAQYVTDRSPSSTD
	NTTGSDQDSPITTTTHALESLSWDICQETDVDLGFYGEFNNLTQKTKED
	QEVFGQFLQEDREEIFEFQGLLDDKEIQEILECSFSEEPDQLVSQGSFMIN
	GDNWSSTDLTRFGI
	AtDof3
	MWLSHLFMSLSKLTCNFSIFSVFMACGSIGMSQVRDTPVKLFGWTITPV
	SHDPYSSSSHVLPDSSSSSSSSSLSLRPHMMNNQSVTDNTSLKLSSNLN
	NESKETSENSDDQHSEITTITSEEEKTTELKKPDKILPCPRCNSADTKFC
	YYNNYNVNQPRHFCRKCQRYWTAGGSMRIVPVGSGRRKNKGWVSSD
	QYLHITSEDTDNYNSSSTKILSFESSDSLVTERPKHQSNEVKINAEPVSQ
	EPNNFQGLLPPQASPVSPPWPYQYPPNPSFYHMPVYWGCAIPVWSTLD
	TSTCLGKRTRDETSHETVKESKNAFERTSLLLESQSIKNETSMATNNHV
	WYPVPMTREKTQEFSFFSNGAETKSSNNRFVPETYLNLQANPAAMARS
	MNFRESI
	AtDof4
	MQSKNMIVASSHQQQQQQPQQPQPQLKCPRCDSSNTKFCYYNNYSL
	SQPRHFCKACKRYWTRGGTLRNVPVGGSYRKNKRVKRPSTATTTTAST
	VSTTNSSSPNNPHQISHFSSMNHHPLFYGLSDHMSSCNNNLPMIPSRFS
	DSSKTCSSSGLESEFLSSGFSSLSALGLGLPHQMSHDHTINGSFINNSTT
	NKPFLLSGLFGSSMSSSSTLLQHPHKPMNNGGDMLGQSHLQTLASLQD
	LHVGGNNEDMKYKEGKLDQISGNINGFMSSSSSLDPSNYNNMWNNAS
	VVNGAWLDPTNNNVGSSLTSLI
	AtDof5
	MATQDSQGIKLFGKTITFNANITQTIKKEEQQQQQPELQATTAVRSPSS
	DLTAEKRPDKIIPCPRCKSMETKFCYFNNYNVNQPRHFCKGCQRYWTA
	GGALRNVPVGAGRRKSKPPGRVGGFAELLGAATGAVDQVELDALLVE
	EWRAATASHGGFRHDFPVKRLRCYTDGQSC
	AtDof6
	MPSEPNQTRPTRVQPSTAAYPPPNLAEPLPCPRCNSTTTKFCYYNNYNL
	AQPRYYCKSCRRYWTQGGTLRDVPVGGGTRRSSSKRHRSFSTTATSSS
	SSSSVITTTTQEPATTEASQTKVTNLISGHGSFASLLGLGSGNGGLDYGF
	GYGYGLEEMSIGYLGDSSVGEIPVVDGCGGDTWQIGEIEGKSGGDSLI

WPGLEISMQTNDVK
AtDof7
MQDLTSAAAYYHQSMMMTTAKQNQPELPEQEQLKCPRCDSPNTKFCY
YNNYNLSQPRHFCKNCRRYWTKGGALRNIPVGGGTRKSNKRSGSSPSS
NLKNQTVAEKPDHHGSGSEEKEERVSGQEMNPTRMLYGLPVGDPNGA
SFSSLLASNMQMGGLVYESGSRWLPGMDLGLGSVRRSDDTWTDLAM
NRMEKN
AtDof8
MDTAKWPQEFVVKPMNEIVTNTCLKQQSNPPSPATPVERKARPEKDQA
LNCPRCNSLNTKFCYYNNYSLTQPRYFCKDCRRYWTAGGSLRNIPVGG
GVRKNKRSSSNSSSSSPSSSSSSKKPLFANNNTPTPPLPHLNPKIGEAAAT
KVQDLTFSQGFGNAHEVKDLNLAFSQGFGIGHNHHSSIPEFLQVVPSSS
MKNNPLVSTSSSLELLGISSSSASSNSRPAFMSYPNVHDSSVYTASGFGL
SYPQFQEFMRPALGFSLDGGDPLRQEEGSSGTNNGRPLLPFESLLKLPV
SSSSTNSGGNGNLKENNDEHSDHEHEKEEGEADQSVGFWSGMLSAGA
SAAASGGSWQ
AtDof9
MSKSRDTEIKLFGRTITSLLDVNCYDPSSLSPVHDVSSDPSKEDSSSSSSS
CSPTIGPIRVPVKKSEQESNKFKDPYILSDLNEPPKAVSEISSPRSSKNNC
DQQSEITTTTTTSTTSGEKSTALKKPDKLIPCPRCESANTKFCYYNNYN
VNQPRYFCRNCQRYWTAGGSMRNVPVGSGRRKNKGWPSSNHYLQVT
SEDCDNNNSGTILSFGSSESSVTETGKHQSGDTAKISADSVSQENKSYQ
GFLPPQVMLPNNSSPWPYQWSPTGPNASFYPVPFYWGCTVPIYPTSETS
SCLGKRSRDQTEGRINDTNTTITTTRARLVSESLRMNIEASKSAVWSKL
PTKPEKKTQGFSLFNGFDTKGNSNRSSLVSETSHSLQANPAAMSRAMN
FRESMQQ
AtDof10
MDPEQEISNETLETILVSSTKGSNNNNKKMEEEMKKKVSRGELGGEAQ
NCPRCESPNTKFCYYNNYSLSQPRYFCKSCRRYWTKGGTLRNVPVGG
GCRRNKRSSSSAFSKNNNNKSINFHTDPLQNPLITGMPPSSFGYDHSIDL
NLAFATLQKHHLSSQATTPSFGFGGDLSIYGNSTNDVGIFGGQNGTYNN
SLCYGFMSGNGNNNQNEIKMASTLGMSLEGNERKQENVNNNNNNSE
NPSKVFWGFPWQMTGDSAGVVPEIDPGRESWNGMVSSWNNGLLNTP
LV
AtDof11
MVFSSVSSFLDPPINWPQSANPNNHPHHHQLQENGSLVSGHHQVLSHH
FPQNPNPNHHHVETAAATTVDPSSLNGQAAERARLAKNSQPPEGALKC
PRCDSANTKFCYFNNYNLTQPRHFCKACRRYWTRGGALRNVPVGGGC
RRNKKGKSGNSKSSSSSQNKQSTSMVNATSPTNTSNVQLQTNSQFPFLP
TLQNLTQLGGIGLNLAAINGNNGGNGNTSSSFLNDLGFFHGGNTSGPV
MGNNNENNLMTSLGSSSHFALFDRTMGLYNFPNEVNMGLSSIGATRVS
QTAQVKMEDNHLGNISRPVSGLTSPGNQSNQYWTGQGLPGSSSNDHH
HQHLM
AtDof12

MATQDSQGIKLFGKTIAFNTRTIKNEEETHPPEQEATIAVRSSSSSDLTAE
KRPDKIIACPRCKSMETKFCYFNNYNVNQPRHFCKGCHRYWTAGGAL
RNVPVGAGRRKSKPPGRVVVGMLGDGNGVRQVELINGLLVEEWQHA
AAAAHGSFRHDFPMKRLRCYSDGQSC
AtDof13
MVFSSIQAYLDSSNWQQAPPSNYNHDGTGASANGGHVLRPQLQPQQQ
PQQQPHPNGSGGGGGGGGGSIRAGSMVDRARQANVALPEAALKCPRC
ESTNTKFCYFNNYSLTQPRHFCKTCRRYWTRGGALRNVPVGGGCRRN
RRTKSNSNNNNNSTATSNNTSFSSGNASTISTILSSHYGGNQESILSQILS
PARLMNPTYNHLGDLTSNTKTDNNMSLLNYGGLSQDLRSIHMGASGG
SLMSCVDEWRSASYHQQSSMGGGNLEDSSNPNPSANGFYSFESPRITS
ASISSALASQFSSVKVEDNPYKWVNVNGNCSSWNDLSAFGSSR
AtDof14
MMNVKPMEQIMIPNNNTHQPNTTSNARPNTILTSNGVSTAGATVSGVS
NNNNNTAVVAERKARPQEKLNCPRCNSTNTKFCYYNNYSLTQPRYFCK
GCRRYWTEGGSLRNVPVGGSSRKNKRSSSSSSSNILQTIPSSLPDLNPPI
LFSNQIHNKSKGSSQDLNLLSFPVMQDQHHHHVHMSQFLQMPKMEGN
GNITHQQQPSSSSSVYGSSSSPVSALELLRTGVNVSSRSGINSSFMPSGS
MMDSNTVLYTSSGFPTMVDYKPSNLSFSTDHQGLGHNSNNRSEALHS
DHHQQGRVLFPFGDQMKELSSSITQEVDHDDNQQQKSHGNNNNNNNS
SPNNGYWSGMFSTTGGGSSW
AtDof15
MQDPAAYYQTMMAKQQQQQQPQFAEQEQLKCPRCDSPNTKFCYYNN
YNLSQPRHFCKSCRRYWTKGGALRNVPVGGGSRKNATKRSTSSSSSAS
SPSNSSQNKKTKNPDPDPDPRNSQKPDLDPTRMLYGFPIGDQDVKGME
IGGSFSSLLANNMQLGLGGGGIMLDGSGWDHPGMGLGLRRTEPGNNN
NNPWTDLAMNRAEKN
AtDof16
MDYSSMHQNVMGVSSCSTQDYQNQKKPLSATRPAPPEQSLRCPRCDS
TNTKFCYYNNYSLSQPRYFCKSCRRYWTKGGILRNIPIGGAYRKHKRSS
SATKSLRTTPEPTMTHDGKSFPTASFGYNNNNISNEQMELGLAYALLNK
QPLGVSSHLGFGSSQSPMAMDGVYGTTSHQMENTGYAFGNGGGGME
QMATSDPNRVLWGFPWQMNMGGGSGHGHGHVDQIDSGREIWSSTVN
YIN
TGALL
AtDof17
MMMETRDPAIKLFGMKIPFPSVFESAVTVEDDEEDDWSGGDDKSPEKV
TPELSDKNNNNCNDNSFNNSKPETLDKEEATSTDQIESSDTPEDNQQTT
PDGKTLKKPTKILPCPRCKSMETKFCYYNNYNINQPRHFCKACQRYWT
AGGTMRNVPVGAGRRKNKSSSSHYRHITISEALEAARLDPGLQANTRV
LSFGLEAQQQHVAAPMTPVMKLQEDQKVSNGARNRFHGLADQRLVA
RVENGDDCSSGSSVTTSNNHSVDESRAQSGSVVEAQMNNNNNNNMN
GYACIPGVPWPYTWNPAMPPPGFYPPPGYPMPFYPYWTIPMLPPHQSSS
PISQKCSNTNSPTLGKHPRDEGSSKKDNETERKQKAGCVLVPKTLRIDD

PNEAAKSSIWTTLGIKNEAMCKAGGMFKGFDHKTKMYNNDKAENSP
VLSANPAALSRSHNFHEQI
AtDof18
MPTSDSGEPRRIAMKPNGVTVPISDQQEQLPCPRCDSSNTKFCYYNNY
NFSQPRHFCKACRRYWTHGGTLRDVPVGGGTRKSAKRSRTCSNSSSSS
VSGVVSNSNGVPLQTTPVLFPQSSISNGVTHTVTESDGKGSALSLCGSF
TSTLLNHNAAATATHGSGSVIGIGGFGIGLGSGFDDVSFGLGRAMWPFS
TVGTATTTNVGSNGGHHAVPMPATWQFEGLESNAGGGFVSGEYFAWP
DLSITTPGNSLK
AtDof19
MERAEALTSSFIWRPNANANAEITPSCPRCGSSNTKFCYYNNYSLTQPR
YFCKGCRRYWTKGGSLRNVPVGGGCRKSRRPKSSSGNNTKTSLTANS
GNPGGGSPSIDLALVYANFLNPKPDESILQENCDLATTDFLVDNPTGTS
MDPSWSMDINDGHHDHYINPVEHIVEECGYNGLPPFPGEELLSLDTNG
VWSDALLIGHNHVDVGVTPVQAVHEPVVHFADESNDSTNLLFGSWSP
FDFTADG
AtDof20
MVFSSLPVNQFDSQNWQQQGNQHQLECVTTDQNPNNYLRQLSSPPTS
QVAGSSQARVNSMVERARIAKVPLPEAALNCPRCDSTNTKFCYFNNYS
LTQPRHFCKTCRRYWTRGGSLRNVPVGGGFRRNKRSKSRSKSTVVVST
DNTTSTSSLTSRPSYSNPSKFHSYGQIPEFNSNLPILPPLQSLGDYNSSNT
GLDFGGTQISNMISGMSSSGGILDAWRIPPSQQAQQFPFLINTTGLVQSS
NALYPLLEGGVSATQTRNVKAEENDQDRGRDGDGVNNLSRNFLGNINI
NSGRNEEYTSWGGNSSWTGFTSNNSTGHLSF
AtDof21
MDATKWTQGFQEMINVKPMEQMISSTNNNTPQQQPTFIATNTRPNATA
SNGGSGGNTNNTATMETRKARPQEKVNCPRCNSTNTKFCYYNNYSLT
QPRYFCKGCRRYWTEGGSLRNVPVGGSSRKNKRSSTPLASPSNPKLPD
LNPPILFSSQIPNKSNKDLNLLSFPVMQDHHHHALELLRSNGVSSRGMN
TFLPGQMMDSNSVLYSSLGFPTMPDYKQSNNNLSFSIDHHQGIGHNTIN
SNQRAQDNNDDMNGASRVLFPFSDMKELSSTTQEKSHGNNTYWNGM
FSNTGGSSW
AtDof22
MDHHQYHHHDQYQHQMMTSTNNNSYNTIVTTQPPPTTTTMDSTTATT
MIMDDEKKLMTTMSTRPQEPRNCPRCNSSNTKFCYYNNYSLAQPRYL
CKSCRRYWTEGGSLRNVPVGGGSRKNKKLPFPNSSTSSSTKNLPDLNP
PFVFTSSASSSNPSKTHQNNNDLSLSFSSPMQDKRAQGHYGHFSEQVV
TGGQNCLFQAPMGMIQFRQEYDHEHPKKNLGFSLDRNEEEIGNHDNF
VVNEEGSKMMYPYGDHEDRQQHHHVRHDDGNKKREGGSSNELWSGI
ILGGDSGGPTW
AtDof23
MNNLNVFTNEDNEMNVMPPPRVCPRCYSDQTRFSYFNNNKKSQPRYK
CKNCCRCWTHGGVLRNIPVTGICDKSNLPKIDQSSVSQMILAEIQQGNH
QPFKKFQENISVSVSSSSDVSIVGNHFDDLSELHGITNSTPIRSFTMDRL

DFGEESFQQDLYDVGSNDLIGNPLINQSIGGYVDNHKDEHKLQFEYES

AtDof24

MDNFNVVANEDNQVNDVKPPPPPPRVCARCDSDNTKFCYYNNYSEFQ
PRYFCKNCRRYWTHGGALRNVPIGGSSRAKRTRINQPSVAQMVSVGIQ
PGSHKPFFNVQENNDFVGSFGASSSSFVAAVGNRFSSLSHIHGGMVTNV
HPTQTFRPNHRLAFHNGSFEQDYYDVGSDNLLVNQQVGGYVDNHNG
YHMNQVDQYNWNQSFNNAMNMNYNNASTSGRMHPSHLEKGGP

AtDof25

MDNLNVFANEDNQVNGLKRPPPSRVCPRCDSDNTKFCFYNNYSESQPR
YFCKNCRRYWTHGGALRNIPVGGSCRKPKRLKVDQSSISEMVSVENQP
INHQSFRQTQENNEFVRSFDASSSATVTAVPNHFGYLSELHGVTNLLPIQ
SFRTMDCLDFGDESFQQGYYDVGSNDLIDNPLINQSIGGYVDNLTSYCI
NQVEPKLQPRYEHES

AtDof26

MDNLNVFANEDNQVNDVKPPPPPPRVCARCDSDNTKFCYYNNYCEFQ
PRYFCKNCRRYWTHGGALRNIPIGGSSRAKRARVNQPSVARMVSVETQ
RGNNQPFSNVQENVHLVGSFGASSSSSVGAVGNLFGSLYDIHGGMVTN
LHPTRTVRPNHRLAFHDGSFEQDYYDVGSDNLLVNQQVGGYGYHMN
PVDQFKWNQSFNNTMNMNYNNDSTSGSSRGSDMNVNHDNKKIRYRN
SVIMHPCHLEKDGP

AtDof27

MDTAQWPQEIVVKPLEEIVTNTCPKPQPQPLQPQQPPSVGGERKARPE
KDQAVNCPRCNSTNTKFCYYNNYSLTQPRYFCKGCRRYWTEGGSLRNI
PVGGGSRKNKRSHSSSSDISNNHSDSTQPATKKHLSDHHHHLMSMSQQ
GLTGQNPKFLETTQQDLNLGFSPHGMIRTNFTDLIHNIGNNTNKSNNNN
NPLIVSSCSAMATSSLDLIRNNSNNGNSSNSSFMGFPVHNQDPASGGFS
MQDHYKPCNTNTTLLGFSLDHHHNNGFHGGFQGGEEGGEGGDDVNG
RHLFPFEDLKLPVSSSSATINVDINEHQKRGSGSDAAATSGGYWTGMLS
GGSWC

AtDof28

MMTSSHQSNTTGFKPRRIKTTAKPPRQINNKEPSPATQPVLKCPRCDSV
NTKFCYYNNYSLSQPRHYCKNCRRYWTRGGALRNVPIGGSTRNKNKP
CSLQVISSPPLFSNGTSSASRELVRNHPSTAMMMMSSGGFSGYMFPLDP
NFNLASSSIESLSSFNQDLHQKLQQQRLVTSMFLQDSLPVNEKTVMFQ
NVELIPPSTVTTDWVFDRFATGGGATSGNHEDNDDGEGNLGNWFHNA
NNNALL

AtDof29

MVFSSFPTYPDHSSNWQQQHQPITTTVGFTGNNINQQFLPHHPLPPQQQ
QTPPQLHHNNGNGGVAVPGGPGGLIRPGSMAERARLANIPLPETALKCP
RCDSTNTKFCYFNNYSLTQPRHFCKACRRYWTRGGALRSVPVGGGCR
RNKRTKNSSGGGGGSTSSGNSKSQDSATSNDQYHHRAMANNQMGPPS
SSSSLSSLLSSYNAGLIPGHDHNSNNNNILGLGSSLPPLKLMPPLDFTDN
FTLQYGAVSAPSYHIGGGSSGGAAALLNGFDQWRFPATNQLPLGGLDP
FDQQHQMEQQNPGYGLVTGSGQYRPKNIFHNLISSSSSASSAMVTATAS

QLASVKMEDSNNQLNLSRQLFGDEQQLWNIHGAAAASTAAATSSWSE
VSNNFSSSSTSNI

AtDof30

MADPAIKLFGKTIPLPELGVVDSSSSYTGFLTETQIPVRLSDSCTGDDDD
EEMGDSGLGREEGDDVGDGGGESETDKKEEKDSECQEESLRNESNDV
TTTTSGITEKTETTKAAKTNEESGGTACSQEGKLKKPDKILPCPRCNSM
ETKFCYYNNYNVNQPRHFCKKCQRYWTAGGTMRNVPVGAGRRKNKS
PASHYNRHVSITSAEAMQKVARTDLQHPNGANLLTFGSDSVLCESMAS
GLNLVEKSLLKTQTVLQEPNEGLKITVPLNQTNEEAGTVSPLPKVPCFP
GPPPTWPYAWNGVSWTILPFYPPPAYWSCPGVSPGAWNSFTWMPQPNS
PSGSNPNSPTLGKHSRDENAAEPGTAFDETESLGREKSKPERCLWVPKT
LRIDDPEEAAKSSIWETLGIKKDENADTFGAFRSSTKEKSSLSEGRLPGR
RPELQANPAALSRSANFHESS

AtDof31

MDHLLQHQDVFGNYNKAREAMGLSYSSNPTPLDNDQKKPSPATAVTR
PQPPELALRCPRCDSTNTKFCYYNNYSLTQPRYFCKSCRRYWTKGGTL
RNIPVGGGCRKNKRSTSSAARSLRTTPEPASHDGKVFSAAGFNGYSNN
EHIDLSLAFALLNKQHPGSSSQLGFHSELGSSHQSDMEGMFGTSQQKE
NATYAFGNGSSGLGDPSRVLWGFPWQMNGESFGMMNIGGGGGHVDQI
DSGREMWTNMNYINSGALM

AtDof32

MQDIHDFSMNGVGGGGGGGGRFFGGGIGGGGGGDRRMRAHQNNILN
HHQSLKCPRCNSLNTKFCYYNNYNLSQPRHFCKNCRRYWTKGGVLRN
VPVGGGCRKAKRSKTKQVPSSSSADKPTTTQDDHHVEEKSSTGSHSSS
ESSSLTASNSTTVAAVSVTAAAEVASSVIPGFDMPNMKIYGNGIEWSTLL
GQGSSAGGVFSEIGGFPAVSAIETTPFGFGGKFVNQDDHLKLEGETVQQ
QQFGDRTAQVEFQGRSSDPNMGFEPLDWGSGGGDQTLFDLTSTVDHA
YWSQSQWTSSDQDQSGLYLP

AtDof33

MLETKDPAIKLFGMKIPFPTVLEVADEEEEKNQNKTLTDQSEKDKTLKK
PTKILPCPRCNSMETKFCYYNNYNVNQPRHFCKACQRYWTSGGTMRS
VPIGAGRRKNKNNSPTSHYHHVTISETNGPVLSFSLGDDQKVSSNRFG
NQKLVARIENNDERSNNNTSNGLNCFPGVSWPYTWNPAFYPVYPYWS
MPVLSSPVSSSPTSTLGKHSRDEDETVKQKQRNGSVLVPKTLRIDDPNE
AAKSSIWTTLGIKNEVMFNGFGSKKEVKLSNKEETETSLVLCANPAALS
RSINFHEQM

AtDof34

MGLTSLQVCMDSDWLQESESSGGSMLDSSTNSPSAADILAACSTRPQA
SAVAVAAAALMDGGRRLRPPHDHPQKCPRCESTHTKFCYYNNYSLSQP
RYFCKTCRRYWTKGGTLRNIPVGGGCRKNKKPSSSNSSSSTSSGKKPS
NIVTANTSDLMALAHSHQNYQHSPLGFSHFGGMMGSYSTPEHGNVGF
LESKYGGLLSQSPRPIDFLDSKFDLMGVNNDNLVMVNHGSNGDHHHH
HNHHMGLNHGVGLNNNNNNGGFNGISTGGNGNGGGLMDISTCQRLM
LSNYDHHHYNHQEDHQRVATIMDVKPNPKLLSLDWQQDQCYSNGGG

	SGGAGKSDGGGYGNGGYINGLGSSWNGLMNGYGTSTKTNSLV
	AtDof35<br>MSSHTNLPSPKPVPKPDHRISGTSQTKKPPSSSVAQDQQNLKCPRCNSP<br>NTKFCYYNNYSLSQPRHFCKSCRRYWTRGGALRNVPIGGGCRKTKKSI<br>KPNSSMNTLPSSSSSQRFFSSIMEDSSKFFPPPTTMDFQLAGLSLNKMN<br>DLQLLNNQEVLDLRPMMSSGRENTPVDVGSGLSLMGFGDFNNNHSPT<br>GFTTAGASDGNLASSIETLSCLNQDLHWRLQQQRMAMLFGNSKEETV<br>VVERPQPILYRNLEIVNSSSPSSPTKKGDNQTEWYFGNNSDNEGVISNN<br>ANTGGGGSEWNNGIQAWTDLNHYNALP
	AtDof36<br>MPSEFSESRRVPKIPHGQGGSVAIPTDQQEQLSCPRCESTNTKFCYYNN<br>YNFSQPRHFCKSCRRYWTHGGTLRDIPVGGVSRKSSKRSRTYSSAATTS<br>VVGSRNFPLQATPVLFPQSSSNGGITTAKGSASSFYGGFSSLINYNAAVS<br>RNGPGGGFNGPDAFGLGLGHGSYYEDVRYGQGITVWPFSSGATDAAT<br>TTSHIAQIPATWQFEGQESKVGFVSGDYVA
Oryza sativa	OsDof1<br>MDDLAAASPPHPPPPPPESHVPPPPQTPEKDSCEDTGDMRISEEKPCTD<br>QELDADQMNSSSFNSSSECENQTPSNDEMTGSESKSEAAQTEGGGSSE<br>EKVLKKPDKILPCPRCNSMDTKFCYYNNYNINQPRHFCKSCQRYWTA<br>GGSMRNLPVGAGRRKSKSSTANYRSILITGSNLAAPAGDAPLYQLSIKG<br>DQTATAVKFAPDSPLCNSMASVLKIGEQSKNAKPTSTAQPRNGETQTCP<br>ASGTTSDSPRNEPVNGAVSGHQNGIVGHSGVPPMHPIPCFPGPPFVYPW<br>SPAWNGIPAMAPPVCTAPAEPANSSDNGSTASVQWSMPPVMPVPGYFP<br>VIPSSVWPFISPWPNGAWSSPWIQPNCSVSASSPTSTSTCSDNGSPVLGK<br>HSRDSKPQGDDKAEKNLWIPKTLRIDDPDEAAKSSIWTTLGIEPGDRSM<br>FRSFQSKPESREQISGAARVLQANPAALSRSQSFQETT
	OsDof2<br>MLSHVEMAPAAGGFKLFGKVIMQCGVSEGTQDKAQGFVVAREKVEPE<br>EEEEEEQRVPAAATSGQRASIKREAADRDEEQRQGGGDAAGQPTQRRL<br>QDSAEARAAAAAPLPCPRCRSRDTKFCYFNNYNVNQPRHFCKACHRY<br>WTAGGALRNVPVGAGRRKNRPLGPLAVAHHNHHHRAAAGFVLGFPN<br>PSSPTSPSPVYTDRWPVTPDRPF
	OsDof3<br>MCDKDPGIKLFGRVIPLAPEAEAAAAADGSDQPEAAAAAAAEVEPAA<br>QDEDHHKETEERKYDEMKVDVPQEEEDNEMKVDAPQEKKDNEVTAD<br>VPEEKGNDEMRVDASESIESIEPVSRSTLDNKKEDQGQMNNVEEKAAS<br>DSKDENEKTANDESGQDKVLKKPDKILPCPRCNSMDTKFCYYNNYNV<br>NQPRHFCKNCQRYWTAGGTMRNVPVGAGRRKSKSSSLHYRHLLMAP<br>DCMMGSRVEISKSMNPEAFASAHSTPIQPIGRNETVLKFGPEVPLCESM<br>ASVLNIQEQNGTNAAAVPTGENQEDNSCISSITSHNVLPENAAQVDKNS<br>TPVYCNGVGPVPQYYLGAPYMYPWNIGWNNVPMMVPGTSMPESASQ<br>SESCSTSSAPWMNMNSPMMPVASRLSAPPFPYPLVPPALWGCLSSWPAT<br>AWNIPWIRTNGGCMSPSSSSNSSCSGNGSPLGKHSRDSSLPLKEDKEEK<br>SLWVPKTLRIDDPDEAAKSSIWATLGIKPGDPGIFKPFQSKGESKGQAAS

ETRPARALKANPAALSRSQSFQETS*
OsDof4
MAALRQGDDPAIKLFGRTIPLLLDPPAAAAAAADEVMPNLGNGVKTN
NDLPLVSDKLLIVKGIPFCPNNSKKNDLQGISRPDGRIEIDSMTEDVKTE
PDGSVPEKILKKPDKILPCPRCNSMETKFCYFNNYNVHQPRHFCRNCQ
RYWTAGGAMRNVPVGAGRRRNKHVSKYCQAMMTCNNTVAPGDVSD
VVHHQVITHGSSLLPATLKENETPTEFISEVPPCKSSASILDIGEPNDTDL
VPLASGDNKEEKSCASSVVVSSCSENLMPDNAIMKEPNNRSGCCNGVA
LPFPTGPALVLPWSLGWNSVALMPATQCSMQPVLGLKDGIPCPPSWPPQ
LMVPAPGICTPVVPIPLVPPLWSCFPGWPNGMWNAQCPGGNTTVLPST
APNKISCSGSSSLVLGKHSREESLQEEEKTRNYLWVPKTLRIDDPAEAA
KSSIWATLGIKPDDKGIFKSFQPNVAKNGTAPESPQALQANPAAFSRSQS
FQETT
OsDof5
MVFPSVPAYLDPPNWNNQQGQPPRPANVGGGDAQHLPVGPTAAAAAP
GEIGGLPTSSSSASAAAAAAQQARPNSMAERARLARAPQPEPALKCPR
CDSTNTKFCYYNNYSLSQPRHFCKTCRRYWTRGGSLRNVPVGGGCRR
NKRSGKSSSAAAAGASSSSSKPSSSAARQLPGGGASPMPSAAASTQPG
GAAAGAIIPPSGLSSMSHHLPFLGAMHPPGPNLGLTFSAGFQPLGGMHH
HVDTADQFPVASGGGATIGASLEQWRVQQQQQQPQQHQFPFLGGAL
ELPPPPPMYQLGLEATRAAGTGATAAAFTLGQTSATATTSRQEGSMKL
EDSKGLEMSLQRQYMAALRQGDGVWGNNNGGNGGSDGGGNGGGGS
WTMNFPGFHSSSGGGGDDGGGVL
OsDof6
MGECKVGGGGGGGDCLIKLFGKTIPVPEPGACAAGDVDKDLQHSGSS
TTEPKTQENTVQDSTSPPPQPEVVDTEDSSADKNSSENQQQQGDTANQ
KEKLKKPDKILPCPRCSSMDTKFCYYNNYNINQPRHFCKNCQRYWTA
GGAMRNVPVGAGRRKSKSVSAASHFLQRVRAALPGDPPLYAPVKTNG
TVLSFGSDLSTLDLTEQMKHLKDKFIPTTGIKNTDEMPVGLCAEGLSKT
EESNQTNLKEKVSADRSPNVAQHPCMNGGAMWPFGVAPPPAYYTSSIA
IPFYPAAAAAVAAYWGCMVPGAWNAPWPPQSQSQSVSSSSAASPVSTM
TNCFRLGKHPRDGDEELDSKGNGKVWVPKTVRIDDVDEVARSSIWSLI
GIKGDKVGADHGRGCKLAKVFESKDEAKASTHTAISSLPFMQGNPAAL
TRSVTFQEGS
OsDof7
MGECRGGGGGGGDGLIKLFGKTIPVQPDAKDVQQHSGSSSSSTESDVQE
TAAVAVADPSPRSEVVDGESPPQPGGEAASHQQQQKEMKLKKPDKILP
CPRCSSMDTKFCYFNNYNVNQPRHFCKHCQRYWTAGGAMRNVPVGA
GRRKNKNATAAAHFLHRVRACAAAAAMPAAPHDATNATVLSFGGGG
GGHDAPPVTLDLADKMTRLGKEGLVAHARNADAAAACSEVSSNRDD
EQIGNTVAKPANGLQQHPPPPHHHHHSAMNGGGIWPYYTSGIAIPIYPA
APAYWGCMIPPPGAWSLPWPATVQSQAISSSSPPTSATPSVSSFTLGKHP
REGGDHEARDHHGNGKVWVPKTIRIDNADEVARSSIRSLFAFRGGDKA
DDNNDDDGTGVHKLATTVFEPKRDSKTAKHPAITSLPLLHTNPVALTRS

ATFQEGS
OsDof8
MLSSHCESMLAYAAAAGRRAVVVDHHQRRYRPNVEVAPNCPRCESPN
TKFCYYNNYSLSQPRYFCKGCRRYWTKGGSLRNVPVGGGCRKNRRG
KAVRAMVGETMTARGGGGGGAAAFSHRFHGPVRPDMILEGMAGSTA
ASAGLGEQPGVAAPDEKPAAADGSTIDLALLYAKFLNHHQPTMAEQGG
GAAVPESVDTSSGSSSDRTTSPAAAQPAAAAAYGPGQDGLVGEPISTEE
HGAAAMARCAQALGELNFSVDQISCYTSLGLPTTDGGDLILPSTLDQH
AKYEPFDSLPEDALSLHDIISGDDDVWCNALGCQGLEAALCRP
OsDof9
MLPYAPRPPSLLVDRRYKQGAEAAPNCPRCDSPNTKFCYYNNYSLSQP
RYFCKGCRRYWTKGGSLRNVPVGGGCRKNRRGKSSSSARSAADAVSS
GRDAAFGHRFPGPVRPDMVLEGMVGNPANPGQAMPDVAAAADGSTI
DLAMLYAKFLNHPPTDAGLGAVTPESGGHVDEAFDTFSASSDLSPGILA
AASAQFDPNQDGFGEWSSPASGNDPTSTATTATTSMLCTDASVQAALG
ELNFAMDQSCFDSLGLPTDVAGAGSLSSWCSIVPSLSTWEEPKYDSLDS
FPDDAMSLHECMIGAPDHDWSVDCQGLEALYMP
OsDof10
MAGAGGAATAAAGGGGGGGVAAGRSGGGGGGGAAAAAGAGAPDPR
AEALRCPRCDSANTKFCYYNNYSLSQPRHFCKACKRYWTRGGTLRNV
PVGGGCRKNKRSRSGGAAPGGGVGRGGPGGGAAAAVSSAGGGAAGT
SPASSLALPQPGSLPSLSSALGLTGGTSLASLLLGSGGSGGDHLGLFQA
MQSVVSDAAAFEMHQQHQSQVDHLLGLGYGAAGAGAQIQAAKPWLHD
GGATGGLLDGFYAPLLSGSIVPGLEELQVKAEATTGDHQQKSSAAAAG
EQSWDLPTPSSSNVEASIIASDALMAAAAASMNPAVSAAAASTAPSAQS
LLYWGNGGIGAAAAAWPDLANCGSSIATLF
OsDof11
MMAGAPPMHICMDSDWLKGIVPEEHGMGSSSPSAELIACPRAPMQAA
AAAADRRLRPQHDQPLKCPRCESTHTKFCYYNNYSLSQPRYFCKTCRR
YWTKGGSLRNVPVGGGCRKNKRAPPKKAAAHAQPAVAVAAALQGRH
METGLHLSFSGMQHHLAPPPPAAATAADPLCSLGLFDWKQYDPVFAGS
GGGGSPVAALESAGGSEAQFMGAGMMGIGGGGVAEYHALSALRFAA
GLGDHLALPFGAVRAEHDAVEVKPVAAERLLSLEWCGEASRTAAPESSI
SSLGGLGLWSGMIGGGHHHHGSSAAI
OsDof12
MQEFQSIPGLAGRLFGGAAAADIRRAQAQQGPASRCGGIPSPEAVKCPR
CESTNTKFCYYNNYNLSQPRHFCKSCRRYWTKGGVLRNVPVGGGCRK
TKRSGSSSAASSAPSTPTAATDNAKNQRRASASSPRSSSGGSGNTSPTA
AAATTPTTPATPSSNTIAVINHATTTTTTTNPFPTDVPPPAPIFADQAAAL
ASLFAPPPPPPLPVFSFAAQAKTEDGIASVLLAGQTTAPTAATVADMTPF
TSLDAGIFELGDVPPAAYWNAGSCWTDVPDPNVYLP
OsDof13
MAPAAGDDAVVPRKGAGGGGTTTPPPPPPAQQQQQQPLPPPPPQEQGL
RCPRCDSPNTKFCYYNNYSLSQPRHFCKTCRRYWTKGGALRNVPVGG

GCRKNKRSRSAAAASRLSLNLPTVEGIGGAAADPAAAAAARLGFLGG
GTTMMMSSSTSPLGGAAAAVADFQQGGAVGMLPLPRLQSPAGGGVGH
HQYVPFGEWPSGDIAGGNAVNGGGGHGAVSSTIASSIESLSFINQDLHW
KLQQQRLATMFLGPPGSASAAAAAAQANDGGGNGAQATASGHAAGG
GGGAFMHMAGSVPSMEAAMPSATSWFMDSCSYGLPSPPPPATAAVAAT
TSSNLNSGGRSSGGDDNATSNCGSAIPAWGDISTFAMLP
OsDof14
MQEFHPVPGLAGRLFGGAAAAAAVAAVEEVRCPRCDSSNTKFCYYNN
YNLSQPRHFCKACRRYWTKGGLLRNVPVGGGCRKPKRPAPPPSSSFTG
GGGGGGGCGHRDSKSARSAGGGGDGSGSTASATATPAAAPASSNTLSA
AVSQPSSVDALSPPPAPMFADQATAFASLFAPPPPPPSQALPAFASFTAQP
KAEEDVADAPALAATEQHRSSSAASFAAHSISPPFAAARSSDGPAAAAA
AAAADWAPPTAVLDAGMFDLAGAIGGDTSYWNAASWTDHDGTIYLP
OsDof15
MEVAEGRTVAAAAAGGGGLGGGARTEAEGLACPRCESTNTKFCYYNN
YNLAQPRHFCKACRRYWTRGGALRNVPVGGGTRNKVAPAPCTGRRK
RAAHAAHAAAPPPTTTASSAPLPLMPPAVAYELPFLPPPPPLPLAAVDPD
RRLLDLGGSFTSLLAPAQLHNGHFTTGFLLGTMSSAPPPPPPPATSTPSP
APAAHPPVSDSIWAMGWPHLSI
OsDof16
MDAAHWHQGLGLVKPMEEMLMAANAAAGANPNPAATAPSSVTGGA
LRGGGGGGAPPVAGGAGAGSTERRARPQKEKALNCPRCNSTNTKFCY
YNNYSLQQPRYFCKTCRRYWTEGGSLRNVPVGGGSRKNKRSSSSAAS
ASPASASTANSVVTSASMSMSMASTGGGASKNPKLVHEGAQDLNLAF
PHHGGLQAPGEFPAFPSLESSSVCNPGGPMGTNGRGGGALSAMELLRS
TGCYMPLQVPMQMPAEYATPGFALGEFRAPPPPPQSSQSLLGFSLDAHG
SVGGPSAAGFGSSAGLQGVPESTGRLLFPFEDLKPTVSSGTGGGGASG
GGAGVDGGHQFDHGKEQQAGGGGGGPGGHDTPGFWNGMIGGGSGT
SW
OsDof17
MDAAHWHQGLGLVKPMEEMLMGANPNPNGSSNQPPPPPSSAASAQR
PIAPPAAGAAAGAGAAGAGAGTERRARPQKEKALNCPRCNSTNTKFC
YYNNYSLQQPRYFCKTCRRYWTEGGSLRNVPVGGGSRKNKRSSSSVV
PSAAASASTSAAVSGSVPVGLAAKNPKLMHEGAQDLNLAFPHHHGRA
LQPPEFTAFPSLESSSVCNPGGNLAAANGAGGRGSVGAFSAMELLRST
GCYVPLPQMAPLGMPAEYAAAGFHLGEFRMPPPPQQQQQQAQTVLG
FSLDTHGAGAGGGSGVFGACSAGLQESAAGRLLFPFEDLKPVVSAAA
GDANSGGDHQYDHGKNQGGGGGVIGGHEAPGFWNSSMIGNGSSNGG
GGGGSW*
OsDof18
MQEQQPETGRRPAQQFATVDLRRPKGYAAAPATPQPGSAATAAAAAGP
AATAAAAAAGEGDPCPRCESRDTKFCYYNNYNTSQPRHFCKCCRRYW
TKGGTLRNVPVGGGTRKKSSSSSSSSSSSSAAAAAPAAKRQKTSKKRR
VTTPEPLAATTPVLTEAAADSAAKTTTEATSEKKTTTSTTTTTPPAPDTT

SEITTELVVPAVEEDSFTDLLQPDSAAVTLGLDFSDYPSITKSLADPDLHF
EWPPPAFDMASYWPAGAGFADPDPTAVFLNLP

OsDof19

MPGQVMEAALQQLPASMASGSLLLPPACLQHPLPAAAAASGGVGGSS
REQCPRCASHDTKFCYYNNYNTSQPRHFCRACRRYWTLGGSLRNVPI
GGSTRKRPRPPVRRPPVHFTAAAAAAAAAAPPHHHHHHHGGPLTPPPA
TSSSSQQAGLLGSLFALGAAPLLEGRVGVGFDLGLGLPGPGHHHAVAG
GGGPAAAVATSSSSSAAAPLLWPTGLLDSSSNNAETWRMAAGGMWPE
FTAAAAQNIRLVIDIGDTTIQVPLNGPTVVQNIGRQAAAAVAGDSSAGG
VSEKTGGAGGGGGEEWMQEQDGLLCMRGRRCGRRGGCLPRPRDWF
AALLAADPAAAAVTRDQAGKAMLYLIVNTCTFATSLAVLPDAVRRRRR
LRVEEGHRVADHQHDDGRRAVRRGDVRALRRRRLPAHGPFVGTVVAA
VTVVVVRCNLALPFRGGDAGHGCSWVSRL

OsDof20

MANLPSTAAAAAADASGFKLFGKVIQPDGQRGVEESAAAQAPPHPHPP
APPVMEAAAAAAGTSQTLQAAGGGGGGGGGGGGEPLPCPRCGSRET
KFCYFNNYNVRQPRHLCRSCRRYWTAGGALRRVASASPGRRRPRPSAA
RSAAAAAASASAASPPAAVPAASEGAESVDSRS

OsDof21

MVFSSLPIFLDPPNWTQMQQQPLQCLIGGGGSDHHHLMPPPSGLAPLPS
AAGAADTAASAPAAAAQQQQPRPAVVSMSERARLARVPLPEPGTLRCP
RCDSTNTKFCYFNNYSLSQPRHFCKACRRYWTRGGALRNVPVGGGCR
RNTKRSTKKSSSSSSRQGGGAGNAAAAATSSSSTTSTSTTATTSSAAAA
AAAAAADVIASMQAGGALLPHHLIGGLPSSAAAAAALEASLEGYHHH
HHAHGHQLPFLQPPPFLQQGLHGYHFADGDVAAGAALADGGFPRGVA
SGLLAQLASVKMEEHGTNNGGGVGGGFVGAHEQYWHGGNGGGGWP
AEFLSGFSSSSSGNVL

OsDof22

MTSASLLLAPRSPDMAAAGILPVSGGGGASSARPVSMAERARMAKIPQ
PEPGLKCPRCDSTNTKFCYFNNYSLTQPRHFCKACRRYWTRGGALRNV
PVGGGFRRNKRGTKPSNSKKPAAAVAGGVMAPPHAQLQLPFGFDGGG
GGGHGSIIGGGGGGGASRLGFPELSSLHAAAAVDYQLGGGGGGDGLG
LERQRLPHFPFLARSNAAVHPPPLMSTAAGVSYPFGDVAAGGLGGDMP
ANAASVAGSAGLITQMASVKMDDIDNHPPPSAATTTASSPIEFLGLRGS
LQFWGGGGGHRGGGDGAGGSAAPGGGGGGWSDLPAFDLSTSGNIL

OsDof23

MASGGALSPVEEKPTVVKTTKAEQHEEEAAVAVKSAAEMMKKSSPCC
PRCNSIKTKFCYYNNYSMAQPRYFCRECRRYWTQGGSLRNVPVGGGC
RKSKRSSASSASASAASPPAPAVGAAPPVVPALSSAISKLLQSEPMAAPC
ADFPNVLPTFVSTGFELPAAAGDRLSLGSFGAFGNLSAAVAAPGGGGGS
STTTSFMDMLRGVGGLFDGVGNSHQMGGNGGGGGSYYAPLITGAGN
GMLMPPPPLPPFSGSLMQHGMQGLFANHAMGGGGGGVMNAGEDGSV
MAGLGGGQWPPALGGADEQQGGGDGGEAVMTKDTGGGASSSASRPD
YFYGWNSAAGGVVAGGGIGGNAAAATGATPWQGLIDSSSAMM

OsDof24

MIFPPAFLDSSSWNDNNNNNHNQQQQQHAHGHHQHHQVAAGCGGG
GGGGDGNSHELLQQQSMIPGTLADGGGGGGAVGPAKPMSMSERARL
ARIPLPEPGLKCPRCDSTNTKFCYFNNYSLSQPRHFCRACRRYWTRGG
ALRNVPVGGGYRRHAKRAKPKPASAAGSASAATTTAGSTPAGSTTTTT
TSSTCATPNAPALPAMLGGNLSILPPLLRLADFDAMSLGSTFSGMAAAA
GKPPPVDAAGCYSVGAATGLEQWRLQQMQSFPFFHAMDHQAAMAAP
PPAMAMPGMFQLGLDGDGHGSGGGEDGGELHHAMPSSKREGYPRGM
YGDHHLAGGYTSYSSATTGNHLL

OsDof25

MAPAVASSPSLVLSAAAATASNKRPADSDASPPHQGDRTGQQEKKQQQ
LECPRCRSTNTKFCYYNNYSTSQPRHFCRACRRYWTHGGTLRDVPVG
GASRRGGGGKRRRVSADADPSSASPPPPTTSTTDAYADLPAGFPFLSDG
AFLPQFGLAGVAPAAFSWASAVPDLYNCGIAPWDDGTAVTGAAWDNFA
DIAGLDLSWPPPGN

OsDof26

MSSPFLGSSSSSASSPLSYLTPPRPPPPPPPLLMGQGYAAAANGGIGGGG
VEAVNAAAAPRQGGRHAGHPPLPRPPPRQCPRCGSANTKFCYYNNYS
RTQPRYLCKACRRHWTEGGTLRDVPVGGGRKNSKRAAGGGKAGATA
STAASAHVVAPAAAPPTSSSFPDLLRQMLMAPATAGGGGGYSIDLTAWQ
QMAAFAAPPQAATGDVGGAVGAASTAAPDANCGGGGVQYWNGWLQ
DDMPGLDGSC

OsDof27

MEAPLHQSPVPLLPPPPPPPRVVGVQQQQQQEAVVPPPPAMAAAAGGG
GREQCPRCASRDTKFCYYNNYNTAQPRHFCRACRRYWTLGGSLRNVP
IGGSTRKRPRPSRPARAAVAAAIAAAAAASASGSQIAAQQQQAPPVVM
SQHEAAAAAAAAASGGGGGDGLLVSLLGAAPVLEGRLGGGIGVDLLGG
EQLGFGAMAMPPAPLLWPARVLEGGDAWKSAAAAAGVSYSPFPALWQ
ELAAAAPVELAGGGGLLRHGGGGAPQLM

OsDof28

MIQELLGGTTMDQLKGASALNHASLPVVLQPIVSNPSPTSSSSTSSRSSA
QATQQRSSSATSSPHGQGQGGGAAEQAPLRCPRCNSSNTKFCYYNNYN
LTQPRHFCKTCRRYWTKGGALRNVPIGGGCRKPRPMPAPVAKPPMSCK
AAPPLGLGGGPVSWASGQQAATAHLMALLNSARGVQGHGGSNVHRL
LGLDTMGHLQILPGAPNGAGAGTAASLWPQSAPRPVTPPPPHMDSQLG
MGTLGHHDVLSSLGLKLPSSASSSPAASYYSDQLHAVVSNAGRPQAPY
DVATASLPCTTAVTSLPSALSSVSAAAPTSNTVGMDLPPVSLAAPEMQY
WNGPAAMSVPWPDLPTPNGAFP

OsDof29

MPPHHGGLMAPRPDMVAAAVAASGGGGGGGGPTGGTAVRPGSMTER
ARLAKIPQPEPGLKCPRCESTNTKFCYFNNYSLSQPRHFCKTCRRYWTR
GGALRNVPVGGGCRRNKRTKSSKSSSSTSAAGSASATGGTSSSTSSTAT
GGSSSAAAAAAMMPPQAQLPFLASLHHPLGGGDHYSSGASRLGFPGL
SSLDPVDYQLGGGAAAAAAIGLEQWRLPQIQQFPFLSRNDAMPPPMSG

	IYPFDAEAAADAAGFAGQLLAGTKVPGSSGLITQLASVKMEDSNAQSA<br>AMNSSPREFLGLPGNLQFWGGGNGAGPGGNGDGATGGSGAGVAPGG<br>GGSGGGWADLSGFNSSSSGNIL<br>OsDof30<br>MQEAGRRPAPQFAGVDLRRPKGYPAAAQLTPAAEEAAAGVGDPCPRC<br>ESRDTKFCYYNNYNTSQPRHFCKSCRRYWTKGGSLRNVPVGGGSRKS<br>STSSSSAAAAAASSSSSPSSPAKSPKRSKNSKRRRVSPPPPQPVPAPPPPT<br>TADAADVAAPTAPEATTKKAPEDLTAAAATQPAVALGLGVADGGGGGK<br>EHLDTSPFEWPSGCDLGPYWPTGVFADTDPSLFLNLP

Table S5 Primers used for qRT-PCR of *DoDof* genes.

Gene ID	Forward primer (5′ – 3′)	Reverse primer (5′ – 3′)
DoDof1	GCCGCCACCTTCTTCTT	GGCTGGTTCACGTTGTAGT
DoDof2	TTCGGGTTGCAGGAATTAGG	GAGGAGCACTTGAGCTAAACA
DoDof3	GGAGAAACGATGGGCAGATT	GAAGAACTGAACCCGGAAAGA
DoDof4	GGACCAAAGGTGGATCTCTAAG	AGAGGATGAATTGGAGGTTGAG
DoDof5	GCGGCTAGTATTTGGGTTAAGA	CCACAGCACCAATGTTCAAATC
DoDof6	GAACTCCAAGCGCTCCTTA	CAACAGTCGGGATGGAAGAA
DoDof7	ATGGAAGTCCTTGGCCTTATAC	CACTGCTTGGTGAAGTTGAAAG
DoDof8	CAATGGACTACGCAGGGAATAG	CTCTTTGTAAGGCCTCACTCTC
DoDof9	GGCGGCTCAAGGAAGAATAA	GGCTCAGTAGGCTGTAGATTTG
DoDof10	TCCTCATCTTCCTCCTCTCTTC	GCAGCTGTGTTAGCACCATA
DoDof11	CACCCTGCAGCCCTTTAT	GAGGTCGGCCATTGATAGTT
DoDof12	CCCTTCCCGATTCTGTAGTTT	TATTTCCGCCGCTCTGTTAG
DoDof13	CCCTTCCCGATTCTGTAGTTT	TATTTCCGCCGCTCTGTTAG
DoDof14	TTGTCGCCATCATCATCTACAA	CTCTGTGTTCTCCTCACCAATC
DoDof15	GGTGCCTGGAGAAAGTAAGAG	CACAGTCCTTAGTCGTCTTCTTC
DoDof16	GCTTCTAGCTCCGGGATTATTT	TCGCCATTTCCATCCCAATAC
DoDof17	AATGGCCTCAACAGGGAATAG	TCGATCTCTCCACTTGGATTTG
DoDof18	GAGTGTTGGTGGAGGCATTA	CCATTTCCCGCTGATGAAGA
DoDof19	CAGAAGCTCCTCTCTGTGATTC	GTTCTCCGCACCAGTAGTAAAG
DoDof20	GGACATCGAAGCTCATCACAT	GCCGTTGGCAGATCATCATA
DoDof21	CTCACCCACCTCTTACTACTCT	GATAGTCTGGCTTCCCATTGAC
DoDof22	CCACGTTGTGACTCTACTAACAC	TCCTTTCGTCCAGTACCTTCTA

Table S6 Primers used for cloning *DoDof4*.

Primer name	Primer sequence (5′ – 3′)
DoDof4-F	ATGGAGATCTCTACAGGCCAC
DoDof4-R	CAAGAGTGAACTATTAATAAGCCCA

Table S7 Physicochemical properties of Dof proteins.

Name	Size(aa)	MW (kDa)	pI	Instability index	Aliphatic index	GRAVY
DhDof1	626	71122.78	9.57	43.59	84.78	-0.244
DhDof2	423	46631.83	6.57	46.5	59.01	-0.801
DhDof3	280	30602.94	8.98	54.72	58.14	-0.696
DhDof4	223	24131.96	8.11	59.4	49.91	-0.54
DhDof5	258	27886.65	5.4	54.51	73.76	-0.231
DhDof6	291	32368.87	5.17	57.36	53.88	-0.567
DhDof7	219	23517.12	9.71	50	49.5	-0.7
DhDof8	358	37355.92	8.59	60.39	62.54	-0.337
DhDof9	203	21772.64	8.91	65.98	61.97	-0.492
DhDof10	228	24649.36	8.85	51.75	57.81	-0.48
DhDof11	336	35618.69	8.9	54.12	55.77	-0.489
DhDof12	199	21655.29	9.53	60.22	47.04	-0.671
DhDof13	223	24299.57	8.97	61.88	71.48	-0.33
DhDof14	484	52734.15	6.44	45.23	61.51	-0.626
DhDof15	483	52644.07	6.61	45.74	61.22	-0.632
DhDof16	266	27963.24	9.32	57.8	53.72	-0.5
DhDof17	293	33533.8	9.21	41.74	48.36	-1.031
DhDof18	308	32247.65	9.08	53.54	52.5	-0.56
DhDof19	471	51019.92	6.67	44.81	58.09	-0.647
DhDof20	259	27880.1	8.43	55.8	58.92	-0.412
DhDof21	264	29635.31	9.13	50.77	62.39	-0.705
DhDof22	271	29466.54	9.06	62.45	50.11	-0.733
DhDof23	276	29365.58	8.9	46.45	52.07	-0.5
DhDof24	319	33892.95	8.48	57.06	57.9	-0.429
DhDof25	188	20965.46	9.02	41.29	47.29	-0.494
DhDof26	292	31239.23	7.72	50.41	52.12	-0.74
DhDof27	145	15875.81	9.19	75.84	52	-0.634
DhDof28	183	19848.92	7.64	49.89	54.48	-0.573
DhDof29	221	25293.61	8.13	57.22	57.38	-0.685
DnDof1	221	25220.55	8.44	60.31	57.38	-0.691
DnDof2	247	27448.37	8.38	55.6	54.86	-0.738
DnDof3	185	19858.24	9.5	58.05	47.95	-0.568
DnDof4	222	24130.51	8.97	59.4	77.48	-0.254
DnDof5	482	52200.62	6.57	45.58	63.4	-0.596
DnDof6	269	28164.55	9.34	56.41	58.18	-0.454
DnDof7	109	12047.56	8.95	56.58	59.17	-0.578
DnDof8	111	12193.79	9.33	69.74	61.62	-0.566
DnDof9	275	29261.41	8.9	46.69	52.25	-0.518
DnDof10	219	23675.28	9.71	54.5	48.17	-0.756
DnDof11	269	30313.07	9.13	53.35	63.38	-0.707
DnDof12	314	33525.55	8.48	57.72	56.34	-0.451

DnDof13	291	31021.02	8.12	51.98	52.3	-0.722
DnDof14	291	32310.79	5.17	57.88	53.57	-0.567
DnDof15	271	29342.4	8.93	62.68	51.22	-0.713
DnDof16	358	37281.79	8.59	61.22	62.29	-0.318
DnDof17	203	21758.61	8.91	68.14	61.03	-0.503
DnDof18	228	24563.27	8.72	54.83	59.08	-0.459
DnDof19	336	35609.68	8.9	52.58	55.77	-0.49
DnDof20	328	37405.41	9.36	46.81	56.55	-0.902
DnDof21	305	32076.5	9.08	52.75	53.02	-0.562
DnDof22	259	27761.02	8.42	53.52	60.42	-0.394
DnDof23	516	55898.61	6.48	45.32	61.34	-0.625
DnDof24	280	30589.95	8.98	55.03	58.14	-0.686
DnDof25	421	46363.53	6.8	48.34	57.22	-0.81
DnDof26	183	19858.96	7.64	46.62	53.93	-0.589
DnDof27	145	15913.82	9.19	78.73	49.31	-0.695
DnDof28	223	24112.9	8.49	59.28	49.46	-0.571
DnDof29	257	27974.66	5.58	52.33	67.59	-0.274
DoDof1	145	15953.92	9.19	77.6	51.31	-0.661
DoDof2	218	23448.01	9.71	50.92	47.94	-0.731
DoDof3	294	32306.78	9.79	64.42	60.17	-0.463
DoDof4	292	31162.18	7.7	52.56	52.12	-0.733
DoDof5	256	27842.59	5.54	54.96	73.59	-0.255
DoDof6	223	24068.81	8.49	59.81	46.86	-0.606
DoDof7	423	46607.78	6.57	48	57.64	-0.811
DoDof8	280	30575.96	9.13	50.95	58.5	-0.668
DoDof9	221	25293.68	8.35	55	53.85	-0.718
DoDof10	276	29391.61	8.9	48.04	51.7	-0.512
DoDof11	144	15749	9.43	63.2	73.33	-0.335
DoDof12	272	29457.59	9.21	63.15	51.36	-0.702
DoDof13	259	27860.13	8.38	56.06	59.31	-0.418
DoDof14	471	51088.12	6.88	46.19	57.88	-0.648
DoDof15	228	24563.27	8.97	45.01	57.81	-0.467
DoDof16	336	35582.66	8.9	52.58	55.77	-0.482
DoDof17	310	32474.87	9.08	51.62	52.16	-0.562
DoDof18	268	28032.39	9.34	54.4	56.94	-0.463
DoDof19	484	52585.94	6.57	46.22	60.72	-0.653
DoDof20	223	24312.62	8.68	62.62	74.13	-0.305
DoDof21	199	21627.22	9.59	56.84	46.08	-0.708
DoDof22	247	27418.39	8.59	54.5	54.86	-0.728

Table S8 Tissue-specific relative expression of *DoDof* genes.

Gene ID	Flowers	Leaves	Roots	Stems
DoDof1	6.23	0.84	0.33	0.65
DoDof2	1.54	2.61	6.64	35.04
DoDof3	6.24	2.04	5.25	5.12
DoDof4	49.62	6.47	26.20	36.62
DoDof5	2.92	0.13	5.26	6.80
DoDof6	18.25	5.40	21.36	24.82
DoDof7	0.27	2.97	22.74	5.04
DoDof8	23.67	4.68	39.38	17.72
DoDof9	24.14	0.09	0.00	2.64
DoDof10	0.00	0.10	0.00	0.11
DoDof11	0.41	0.38	3.49	0.21
DoDof12	10.66	27.08	8.16	13.40
DoDof13	8.35	3.21	6.68	8.75
DoDof14	7.27	46.16	37.14	7.78
DoDof15	7.81	1.81	1.87	7.90
DoDof16	1.67	3.37	0.97	3.64
DoDof17	104.22	5.29	42.43	9.40
DoDof18	4.61	3.57	3.68	7.40
DoDof19	2.35	2.58	36.65	7.84
DoDof20	9.27	1.73	1.85	2.60
DoDof21	6.41	0.00	0.00	0.00
DoDof22	6.46	1.21	6.50	7.21

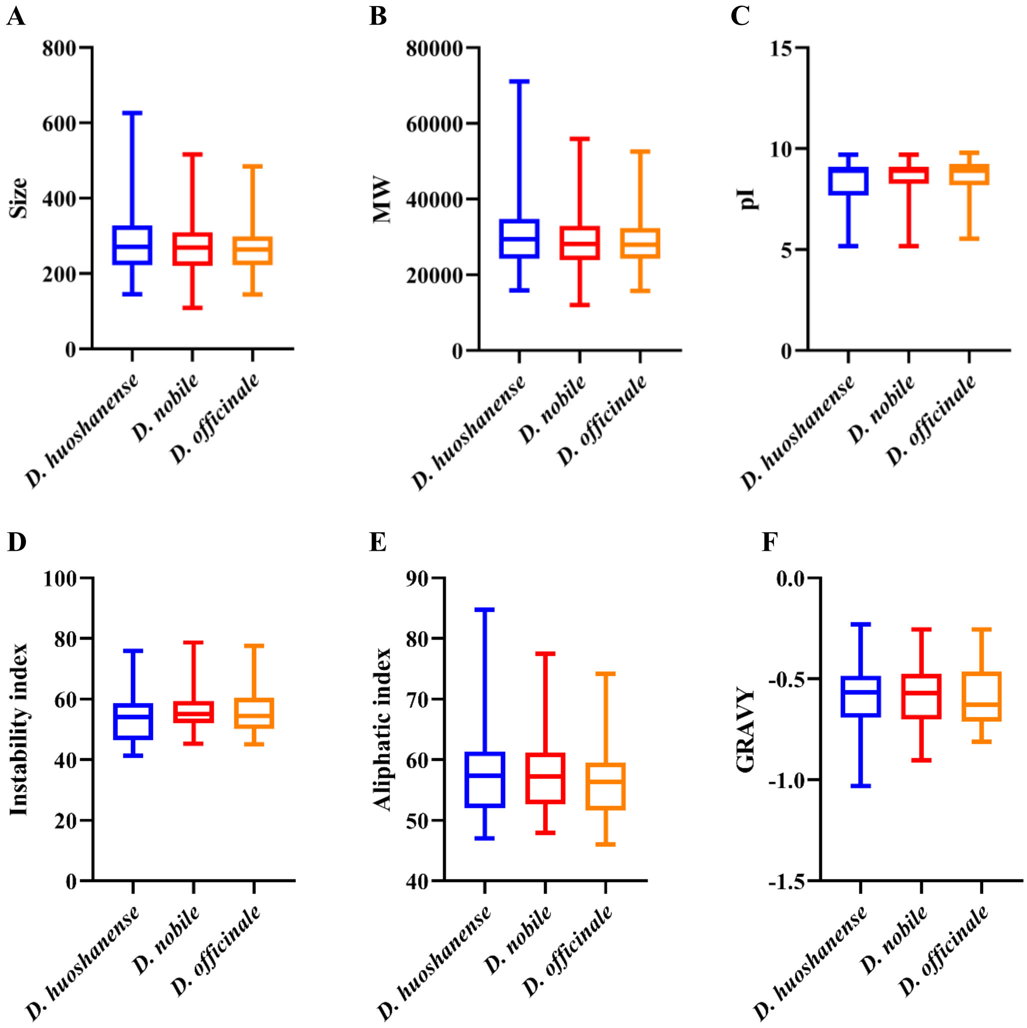

Figure S1 Physicochemical properties analysis of *DhDof*, *DnDof*, *DoDof* genes. Average size (A), MW (B), pI (C), instability index (D), aliphatic index (E), and GRAVY (F) of *DhDof*, *DnDof*, *DoDof* genes.

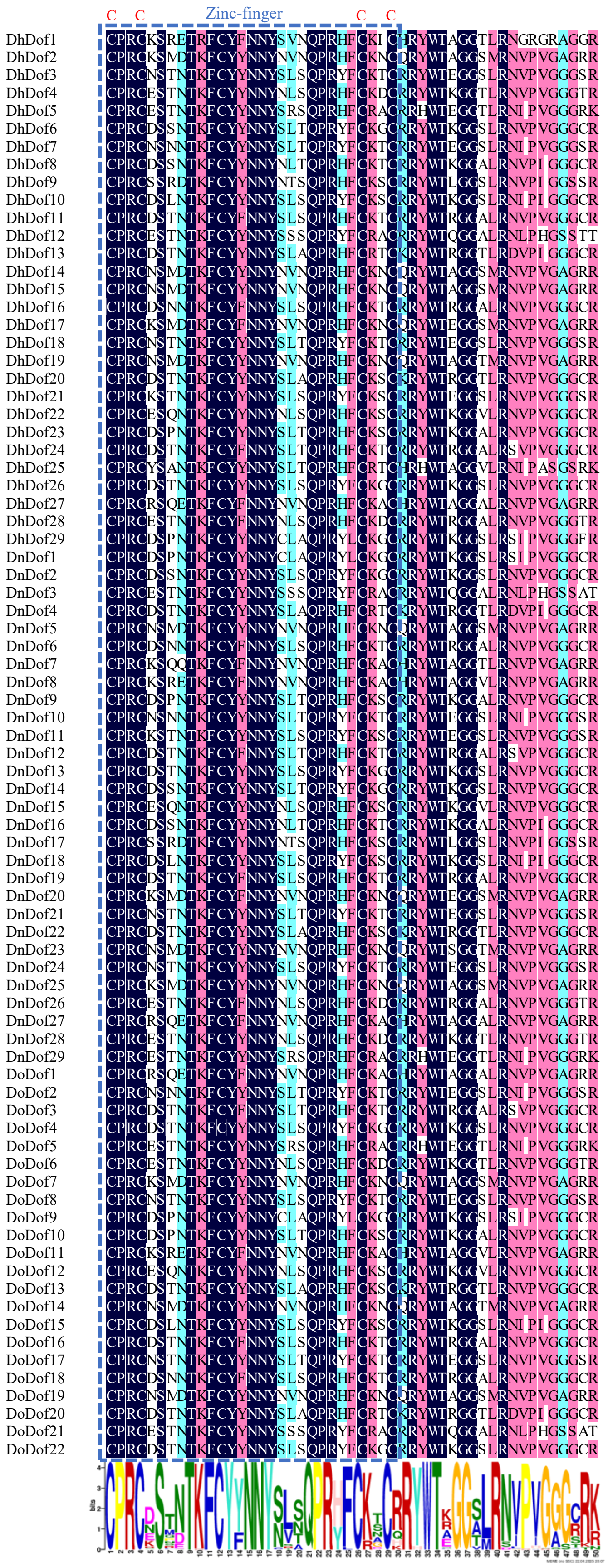

Figure S2 Multiple sequence alignment Dof proteins.

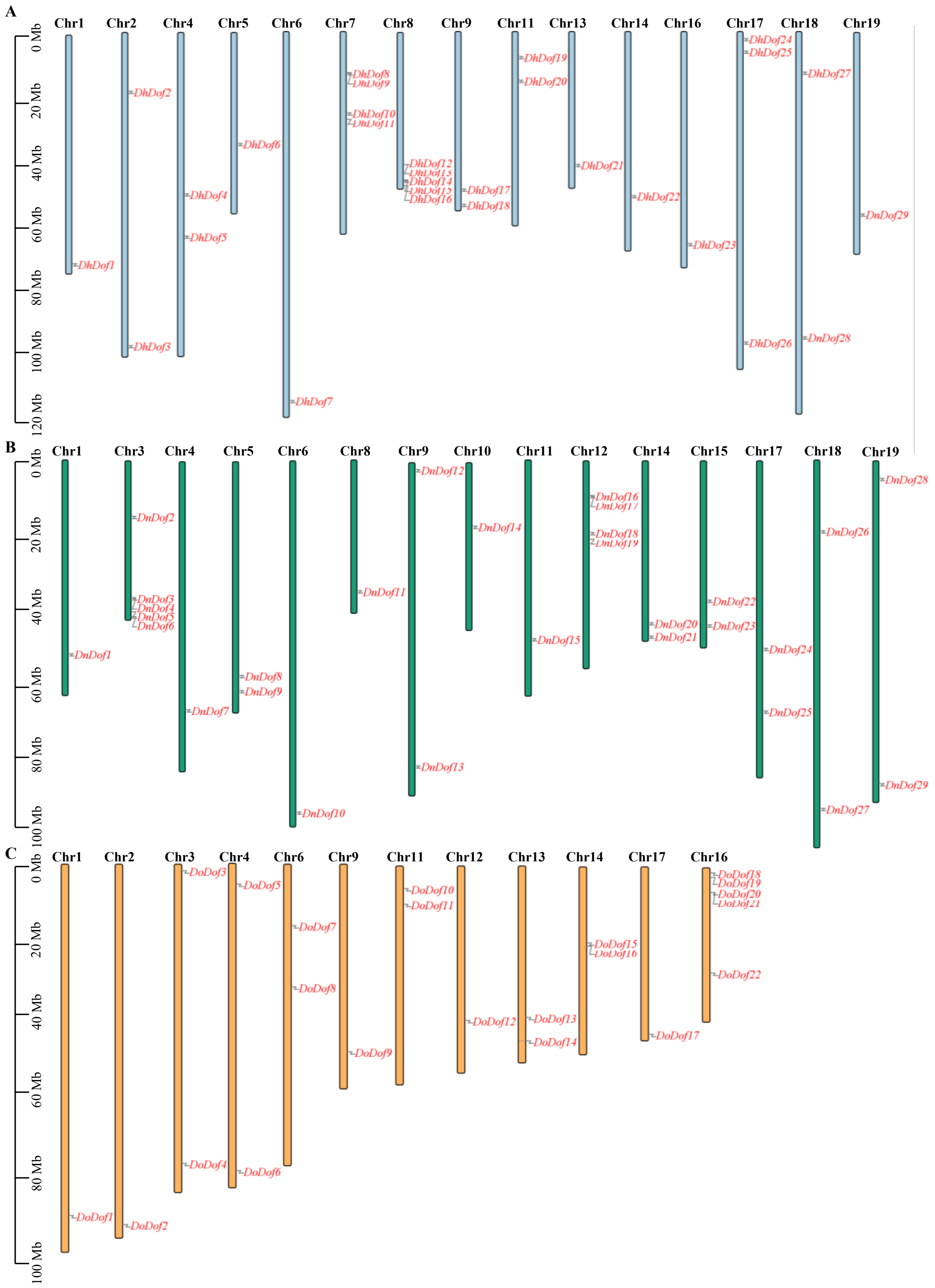

Figure S3 Chromosome localization of *DhDof*, *DnDof*, *DoDof* genes.

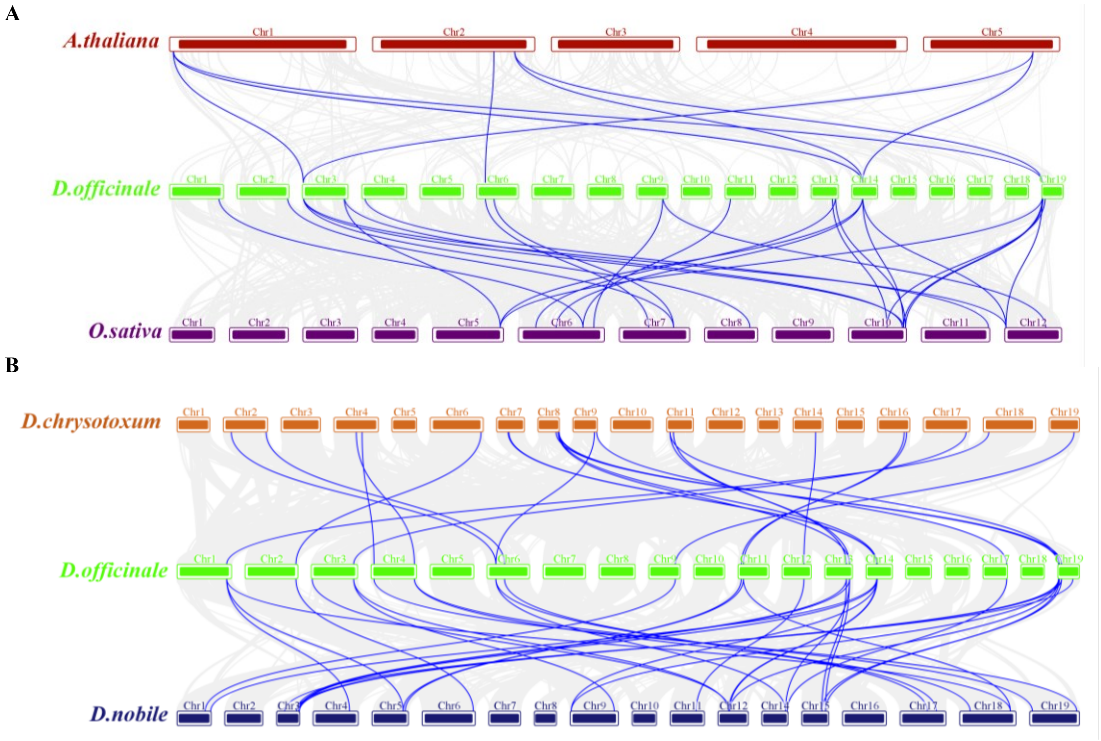

Figure S4 Collinearity analysis of *DhDof*, *DnDof*, *DoDof* genes.

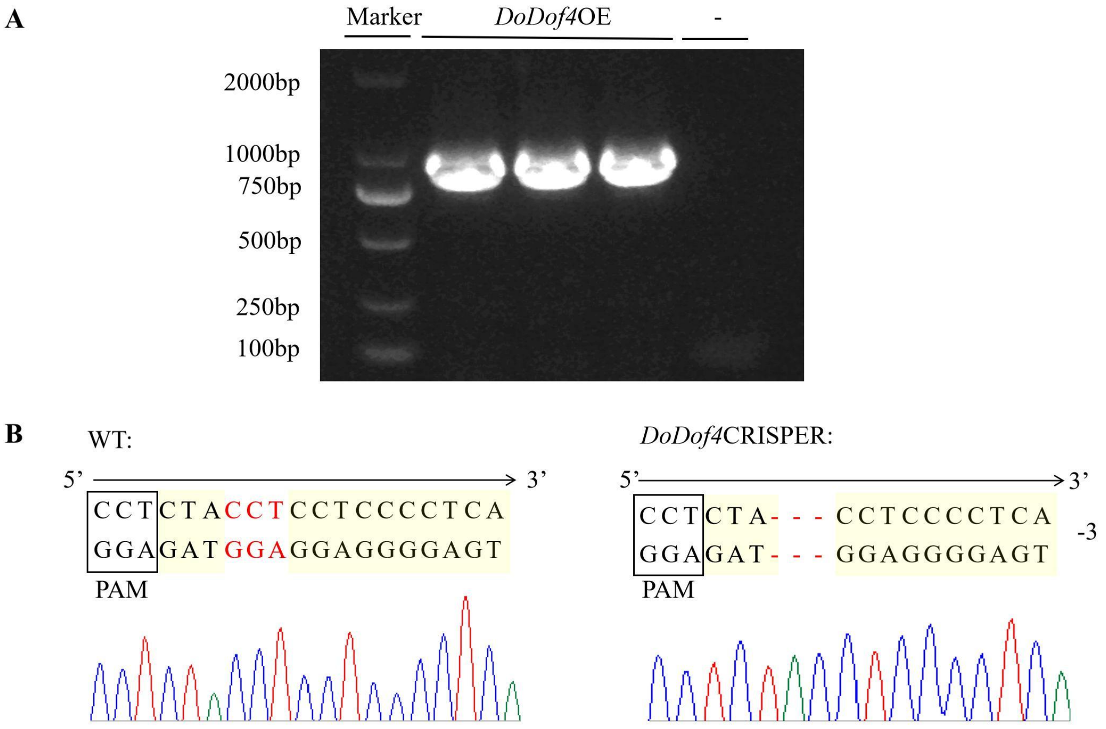

Figure S5 Identification of *DoDof4* positive transgenic PLBs. (A) To positive identification of *DoDof4*OE by PCR. "-" means negative control. (B) Sequencing results of *DoDof4*CRISPR PLBs. PLBs, protocorm-like bodies. WT, wild-type *DoDof4*PLB.

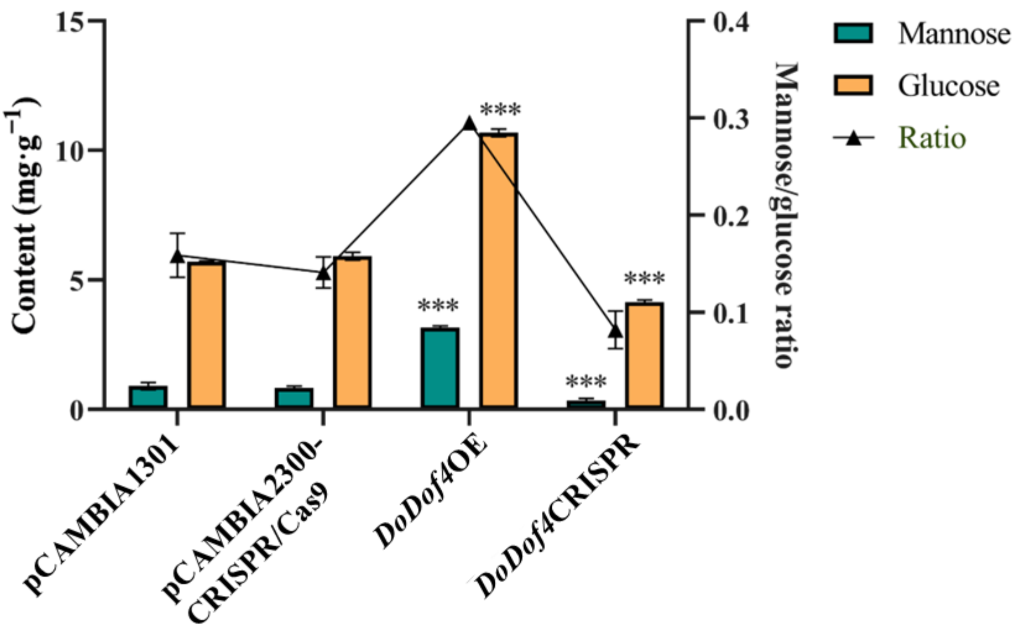

Figure S6 Quantitative analysis of mannose and glucose content in transgenic PLBs. PLBs, protocorm-like bodies.

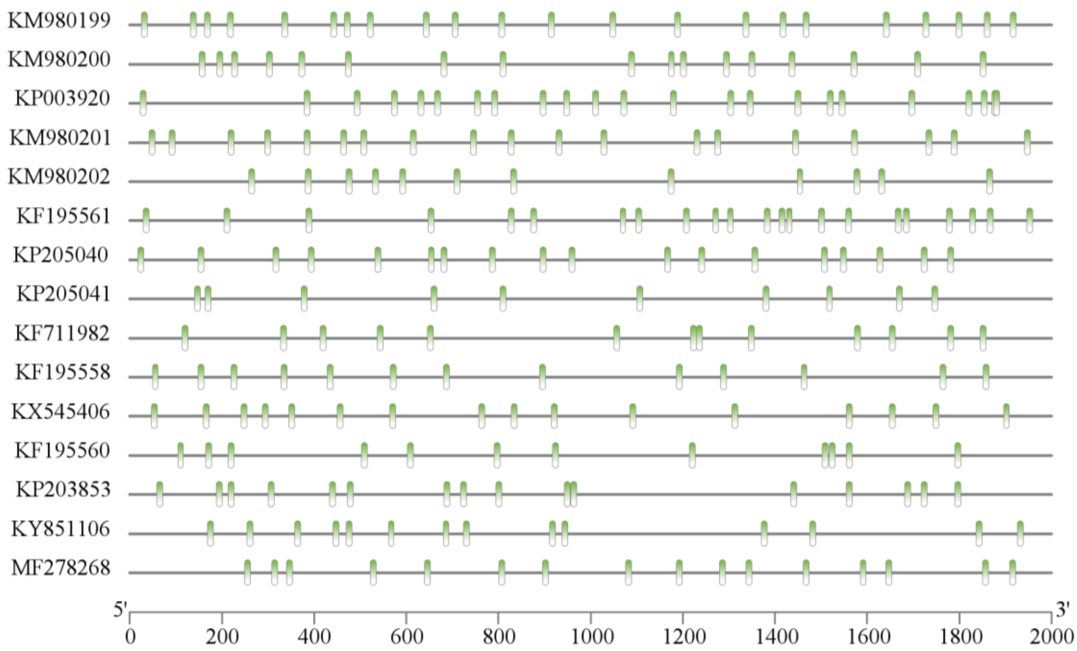

Figure S7 Schematic diagram of the promoters of key enzymes related to the biosynthesis of WSPs. Green boxes indicate potential DNA-binding sites of Dof TFs.
